# Suggesting disease associations for overlooked metabolites using literature from metabolic neighbors

**DOI:** 10.1093/gigascience/giad065

**Published:** 2023-09-15

**Authors:** Maxime Delmas, Olivier Filangi, Christophe Duperier, Nils Paulhe, Florence Vinson, Pablo Rodriguez-Mier, Franck Giacomoni, Fabien Jourdan, Clément Frainay

**Affiliations:** Toxalim (Research Center in Food Toxicology), Université de Toulouse, INRAE, ENVT, INP-Purpan, UPS, 31300 Toulouse, France; IGEPP, INRAE, Institut Agro, Université de Rennes, Domaine de la Motte, 35653 Le Rheu, France; Université Clermont Auvergne, INRAE, UNH, Plateforme d’Exploration du Métabolisme, MetaboHUB Clermont, F-63000 Clermont-Ferrand, France; Université Clermont Auvergne, INRAE, UNH, Plateforme d’Exploration du Métabolisme, MetaboHUB Clermont, F-63000 Clermont-Ferrand, France; Toxalim (Research Center in Food Toxicology), Université de Toulouse, INRAE, ENVT, INP-Purpan, UPS, 31300 Toulouse, France; MetaboHUB-Metatoul, National Infrastructure of Metabolomics and Fluxomics, Toulouse, 31300, France; Toxalim (Research Center in Food Toxicology), Université de Toulouse, INRAE, ENVT, INP-Purpan, UPS, 31300 Toulouse, France; Université Clermont Auvergne, INRAE, UNH, Plateforme d’Exploration du Métabolisme, MetaboHUB Clermont, F-63000 Clermont-Ferrand, France; Toxalim (Research Center in Food Toxicology), Université de Toulouse, INRAE, ENVT, INP-Purpan, UPS, 31300 Toulouse, France; MetaboHUB-Metatoul, National Infrastructure of Metabolomics and Fluxomics, Toulouse, 31300, France; Toxalim (Research Center in Food Toxicology), Université de Toulouse, INRAE, ENVT, INP-Purpan, UPS, 31300 Toulouse, France

**Keywords:** literature mining, Bayesian statistics, metabolic network

## Abstract

In human health research, metabolic signatures extracted from metabolomics data have a strong added value for stratifying patients and identifying biomarkers. Nevertheless, one of the main challenges is to interpret and relate these lists of discriminant metabolites to pathological mechanisms. This task requires experts to combine their knowledge with information extracted from databases and the scientific literature. However, we show that most compounds (>99%) in the PubChem database lack annotated literature. This dearth of available information can have a direct impact on the interpretation of metabolic signatures, which is often restricted to a subset of significant metabolites. To suggest potential pathological phenotypes related to overlooked metabolites that lack annotated literature, we extend the “guilt-by-association” principle to literature information by using a Bayesian framework. The underlying assumption is that the literature associated with the metabolic neighbors of a compound can provide valuable insights, or an *a priori*, into its biomedical context. The metabolic neighborhood of a compound can be defined from a metabolic network and correspond to metabolites to which it is connected through biochemical reactions. With the proposed approach, we suggest more than 35,000 associations between 1,047 overlooked metabolites and 3,288 diseases (or disease families). All these newly inferred associations are freely available on the FORUM ftp server (see information at https://github.com/eMetaboHUB/Forum-LiteraturePropagation).

**Key Points:**

- Most metabolites have little or no information available in the literature.
- We propose an original method leveraging information contained in the literature from metabolic neighbors.
- We provide more than 35000 suggested relations between overlooked metabolites and disease-related concepts.

## Background

Omics experiments have become widespread in biomedical research and are frequently used to study pathologies at the genome, transcriptome, proteome, and metabolome levels. The subsequent discriminant analysis leads to a set (a signature) of genes, proteins, or metabolites, reflecting alterations of the phenotype at different levels of postgenomic processes. The interpretation of these signatures requires gathering knowledge about each of its elements from the scientific literature and dedicated databases (DisGeNET [1], Uniprot [2], HMDB [3], CTD [4], MarkerDB [5], FORUM [6]). However, the scientific literature suffers from an imbalanced knowledge distribution. This topic has received much attention for genes and proteins [7–11], showing a highly skewed distribution of the number of articles mentioning each entity. Indeed, what is known as *the Matthew effect* [12], which refers to the saying “the rich get richer,” is particularly valid in scientific communications. For instance, as reported in [8], “more than 75% of protein research still focuses on the 10% of proteins that were known before the genome was mapped,” and as reported in [11], “all genes that had been reported upon by 1991 (corresponding to 16% of all genes) account for 49% of the literature of the year 2015.”

While we are getting closer to a complete reconstruction of the human genome [13], our knowledge of the metabolome (i.e., the set of metabolites present in a biological system [14]) is still limited. This is also reflected in the distribution of the number of articles mentioning each compound present in the PubChem database. While only a small fraction of them are mentioned in thousands of articles, the majority remains rarely or never mentioned [15]. This imbalance has consequences for the interpretation of the signatures, which can rely solely on a subset of its members that are sufficiently covered to provide insights. In human health research, it is therefore critical to bring knowledge to these overlooked compounds by suggesting diseases that could be linked to them.

A metabolite is suspected to be impacted or involved in a particular disease through metabolism when an imbalance in its abundance has been observed in comparison to control cases. Moreover, metabolites are linked to each other by biochemical reactions, and therefore their abundances are also interdependent. Among other factors, the abundance of a compound can depend on the concentration of its precursors and, in turn, can also influence the rate of production of other compounds. Following the well-known “guilt-by-association” principle, we assume that if a metabolite has been linked to a particular disease due to an imbalance in its abundance, metabolites that are connected to it by biochemical reactions (i.e., its metabolic neighborhood) can also be suspected of being linked to this disease. Metabolic networks [16], built originally for modeling purposes, describe those substrate–product relations between compounds and thus provide a suitable support to extend these suspicions to metabolic neighbors. For humans, the reconstruction of the metabolic network (Human1 v1.7 [17]) contains 13,082 reactions and 8,378 metabolites. In other omics fields, network-based strategies following the “guilt-by-association” principle have been applied to build several recommendation systems proposing new genes or proteins that could be related to a given disease from a list of known genes/proteins [18–20]. We also developed a similar approach for metabolic signatures using random walks in metabolic networks [21].

If a compound is rarely or never mentioned, we hypothesize that the literature in its surrounding neighborhood may provide *a priori* knowledge on its biomedical context. To combine both this *a priori* and the available literature of the compound (if any) in the suggestions, we propose a method based on the Bayesian framework. The method returns several predictors to evaluate whether a metabolite could be related to a disease. In addition, several indicators can be used to highlight the most influential metabolic neighbors in the suggestions.

Metabolic neighborhoods were defined from the Human1 metabolic network [17], and co-mention data between metabolites and diseases were extracted from the FORUM Knowledge Graph (KG) [6]. The detailed workflow is presented in Supplementary Fig. S2. FORUM contains significant associations between PubChem chemical compounds and MeSH biomedical descriptors based on their co-mention frequency in PubMed articles. We evaluated our hypothesis by testing whether significant associations between metabolites and diseases could be retrieved solely on the basis of the literature of their neighbors. We illustrate the behavior of the method in 2 scenarios: a metabolite for which the prior is the only source of information (hydroxytyrosol) and a rarely mentioned metabolite (5α-androstane-3,17-dione with 82 articles). Using this approach on human metabolic network, we suggested more than 35,000 new relations between overlooked metabolites and diseases (and disease families). The code and the data needed to reproduce the results are available at [22].

## Method and Data Description

The core of the method is the construction of a prior distribution on the probability that an article mentioning a metabolite would also mention a particular disease. This distribution is estimated from the literature of its metabolic neighborhood. The metabolic neighborhood of a compound consists of the metabolites that can be reached through a sequence of biochemical reactions. It is defined from the Human1 metabolic network [17], which was pruned from spurious connections using an atom-mapping procedure [21] (see Supplementary S1.1). In this study, we define a set of overlooked compounds as compounds with fewer than 100 retrieved articles mentioning the compound, which correspond to orders of magnitude below 4,799, the mean number of retrieved articles per compound (when any), and is close to the median number of articles, 172. It is worth mentioning that such a threshold serves solely as a prioritization criterion, since the method applicability is not restricted to a given range of mentioning corpus sizes (although its relevance is less obvious when a sufficient corpus is already available). In the following description of the method and subsequent analyses, a distinction is also made between metabolites without any retrieved articles (1) and metabolites with fewer than 100 annotated articles (2).

**Figure 1:**
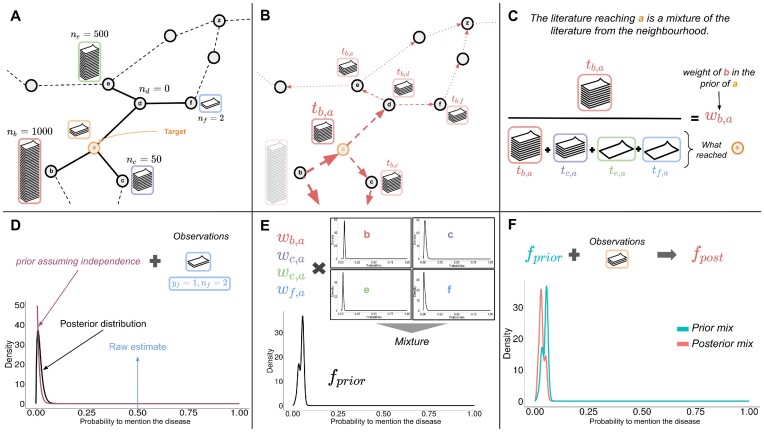
A step-by-step description of the proposed method. Compound $\boldsymbol {a}$ has $0 < n_{\boldsymbol {a}} \le 100$ articles, with some co-occurrence with the disease of interest ($0 \le y_{\boldsymbol {a}} \le n_{\boldsymbol {a}}$). In the blocks **A** and **B**, the nodes represent metabolites and the edges substrate–product relationships in the metabolic network. Dashed lines indicate more distant connections. (A) Imbalance of mentioning literatures within a metabolic network. Compound *a* has $0 < n_{\boldsymbol {a}} \le 100$ articles, with some co-occurrence with the disease of interest ($0 \le y_{\boldsymbol {a}} \le n_{\boldsymbol {a}}$). Nodes represent metabolites and the edges substrate–product relationships in the metabolic network. Dashed lines indicate more distant connections. (B) Propagation of literature through a metabolic neighborhood. (C) Weight of a metabolic neighbor in an overlooked metabolite’s corpus used for prior construction. (D) Contribution of a neighbor, from assumed independence, mitigated by a neighbor’s literature (observations). (E) Construction of the metabolite’s prior from contributors. (F) Computation of the metabolite’s posterior from observations and the prior.

Figure 1 summarizes all the steps in the proposed method. Figure 1A introduces the example of a relation between an overlooked metabolite $\boldsymbol {a}$ and a disease. The prior distribution on the probability that an article mentioning $\boldsymbol {a}$ would also mention the disease is built from a mixture of the literature of its close neighborhood in the metabolic network. The weight of the component of these metabolites in the mixture depends on both their distance to $\boldsymbol {a}$ and their number of annotated articles (see details in section *Estimating the contributions of metabolic neighbors* in Methods). We also impose that a metabolite cannot influence its own prior. As an illustration, $\boldsymbol {b}$ shares a quantity $t_{\boldsymbol {b},\boldsymbol {a}}$ of its literature to build the prior of $\boldsymbol {a}$ but does not influence its own prior (cf. Fig. 1B). The weight of $\boldsymbol {b}$ in the prior of $\boldsymbol {a}$ is then estimated as the number of articles it had shared with $\boldsymbol {a}$ relative to the other neighbors $\boldsymbol {c}$, $\boldsymbol {e}$, and $\boldsymbol {f}$ (see Fig. 1C). We refer to $\boldsymbol {b}$, $\boldsymbol {c}$, $\boldsymbol {e}$, and $\boldsymbol {f}$ as the *contributors* to the prior of $\boldsymbol {a}$. Each contributor has a weight *w* in the prior of $\boldsymbol {a}$ (e.g., $w_{\boldsymbol {b},\boldsymbol {a}}$) proportional to its contribution. By analogy, it is as if each metabolite spreads its literature in the metabolic network, and the prior of $\boldsymbol {a}$ was built from the articles it had received from its contributors.

In Fig. 1D, the contributor $\boldsymbol {f}$ is also an overlooked metabolite with only 2 annotated articles, including one mentioning the disease. This results in a small sample size available to estimate the probability that an article mentioning $\boldsymbol {f}$ also mentions the disease, which may lead to unreliable and spurious contributions. To address this, a shrinkage procedure is applied to all contributors, assuming that *a priori*, mentioning a metabolite in an article does not affect the probability of mentioning a particular disease. In Bayesian settings, a shrinkage estimator integrates information from the prior to readjusted raw estimates, reducing the effect of sampling variations (further details in section *Mixing neighboring literature to build a prior* in Methods).

Then the prior distribution of $\boldsymbol {a}$ is built as a mixture of the probability distributions of individual contributors ($\boldsymbol {b}$, $\boldsymbol {c}$, $\boldsymbol {e}$, and $\boldsymbol {f}$), as illustrated in Fig. 1E. Recall that the weight of each contributor in the mixture is $w_{{\bf .},\boldsymbol {a}}$, as estimated in the previous step (see Fig. 1C). The prior mixture distribution is denoted by *f_prior_*. The constructed prior distribution for $\boldsymbol {a}$ represents the probability distribution that an article from one of its contributors would mention the disease. In the scenario where $\boldsymbol {a}$ has no literature (1), the predictions will be based solely on *f_prior_*.

However if $\boldsymbol {a}$ is mentioned in few articles (2), we compute the posterior distribution, thus updating the weights and distributions of each contributor in the mixture (Fig. 1E). The posterior mixture distribution is denoted by *f_post_*.

From the mixture distribution, 2 predictors are estimated: *LogOdds* and *Log*_2_*FC. LogOdds* expresses the ratio between the probability of the disease being mentioned more frequently than expected in the literature of the compound, rather than less frequently. *Log*_2_*FC* expresses the change between the average probability of mentioning the disease in the mixture distribution, compared to the expected probability in the whole literature. In summary, both should be considered jointly in the predictions: *LogOdds* as a measure of significance and *Log*_2_*FC* as a measure of effect size. In (2), to get an intuition about the belief of the neighborhood only, we also return similar indicators estimated from *f_prior_*: *priorLogOdds* and *priorLog*_2_*FC* (see sections *Updating prior and selecting novel associations* and *Different scenarios* in Methods). Finally, given its primary role in driving predictions, assessing the composition of the constructed prior is crucial. Essentially, the more contributors to the prior, close to the target compound, with balanced weights, the better it captures the neighborhood literature and increases the confidence in predictions. To aid in this evaluation, a set of diagnostic indicators is presented in Supplementary S1.3.

## Analyses

### Unbalanced distribution of the literature related to chemical compounds

**Figure 2:**
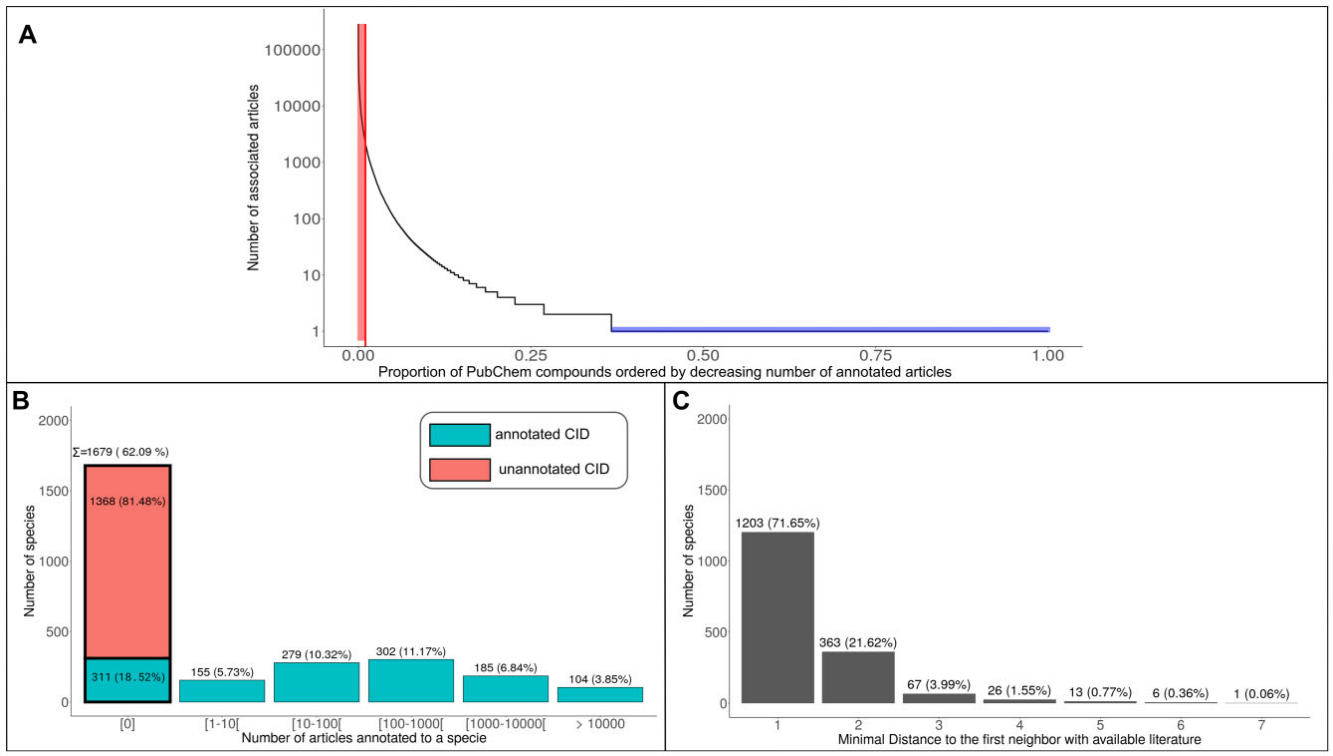
(A) Distribution of the number of annotated articles (expressed in log-scale) for PubChem compounds that have at least 1 article in FORUM, in descending order. The red area represents the proportion of the most mentioned compounds required to attain 80% of the total number of annotations, while the blue area represents the fraction of compounds with only one annotated article. (B) Distribution of the number of annotated articles per metabolites, organized by bins, in the carbon skeleton graph of Human1. The first bar represents the metabolites without literature. Among them, 81.5% do not have annotated PubChem identifiers, making it impossible to link them to PubMed articles with FORUM. The remaining 18.5% have annotated PubChem identifiers, but no articles were found mentioning them. In total, there are 1,336 compounds with an available PubChem identifier. (C) Distribution of the shortest distance to the first neighbor in the metabolic network with at least 1 annotated article, for the metabolites without literature in the network (bold bar of B). The distances were computed with the Dijkstra algorithm.

The FORUM KG links PubChem compounds to the PubMed articles that mention them. Among the 103 million PubChem compounds in FORUM, only 376,508 are mentioned in PubMed articles, representing a coverage lower than 0.4%. For these mentioned compounds, the distribution of the literature is highly skewed (Fig. 2A). The top 1% of the most mentioned compounds (red area) concentrates 80% of the links between PubChem compounds and PubMed articles. Similarly, the blue area indicates that 63% of compounds (218,291) have only 1 article mentioning them, which, to give a point of comparison, is cumulatively less than the literature associated with glucose: 278,277 distinct articles.

Considering only metabolites, Fig. 2B presents the distribution of the number of articles mentioning the 2,704 metabolites, conserved in the pruned version of the Human1 metabolic network. Because of the skewed distribution of the literature and the lack of external identifiers, 62.09% of the metabolites in the metabolic network have no annotated articles. Nevertheless, almost 72% of them have at least 1 direct neighbor in the metabolic network with available literature (see Fig. 2C). Moreover, by considering the close neighborhood (paths up to 3 reactions), almost all the metabolites ($\approx 97.26 \%$) without initial literature can reach a described neighbor, showing the availability of nearby literature to build a prior.

### Evaluation of the prior computation

The critical step in the proposed method is the construction of a relevant prior. While its influence on the results will decrease as the size of the literature of the targeted compound increases, it will mainly drive the predictions for the rarely mentioned compounds we are interested in [23].

The relevance of the prior was evaluated by testing whether significant associations with diseases could be retrieved using only the literature from the metabolic neighborhood of the metabolite.

The validation dataset includes 10,000 significant relations between metabolites and disease-related MeSH extracted from the FORUM KG and 10,000 random metabolite–MeSH pairs to serve as negative examples. The method is evaluated by considering either the direct or a larger neighborhood (metabolites that can be reached through a path of 2 or more reactions). We therefore focused on 2 specific settings: α = 0, where solely the direct neighbors contribute to the prior, and α = 0.4, where contributions between direct or indirect neighbors are relatively balanced. The impact of the parameter α on the construction of the prior and the precision–recall trade-off is extensively evaluated in Supplementary Material S4.3.

**Figure 3:**
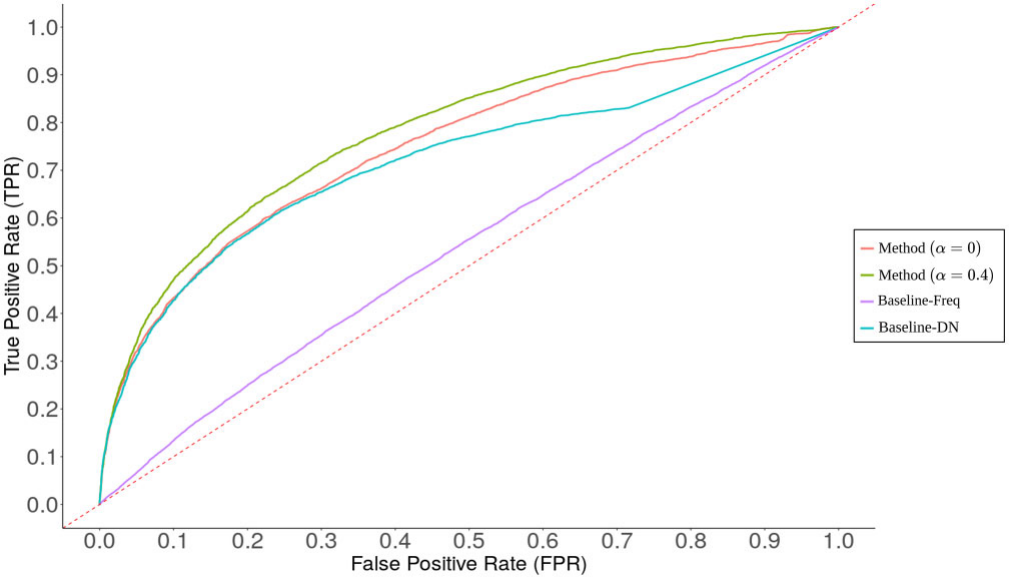
Receiver operating characteristic (ROC) of the method considering only the direct neighborhood (α = 0) or a larger neighborhood (α = 0.4) and 2 different baselines. For Baseline-Freq, the predictions are only based on the overall probability of mentioning the disease in the literature. For Baseline-DN, the predictions are based on the ratio between the average probability of mentioning the disease in the direct neighborhood and its overall probability. Respective areas under the curve (AUCs) for Method(α = 0), Method(α = 0.4), Baseline-DN, and Baseline-Freq are 0.75, 0.78, 0.72, and 0.54. A true positive represents an association between a compound and a MeSH term that is retrieved from the compound’s mentioning corpus using the Fisher exact test and from methods in which no knowledge of such a corpus is available. A false positive is only retrieved from the latter.

We decided to compare the proposed method against 2 different baselines (more details in Supplementary S4.2). Baseline-Freq is the most naive approach in which the predictions are solely based on the overall probability of mentioning the disease, such that a metabolite is more likely to be related to frequently mentioned diseases in the literature. Hence, Baseline-Freq ignores the network information (metabolic neighborhood). On the contrary, the predictions with Baseline-DN are based on the average probability of mentioning the disease in the direct neighborhood and thus closer to the proposed approach. It is worth noting that, if all direct neighbors have relatively the same number of annotated articles and are well covered (negligible shrinkage), the method parameterized with α = 0 behaves like the simple Baseline-DN for metabolites without literature. We used *Log*_2_*FC* as a predictor for the proposed method in Fig. 3.

The evaluation results on the validation dataset for all described approaches are presented in Fig. 3. All tested approaches outperform Baseline-Freq, showing the benefit of examining the neighboring literature. When considering the direct neighborhood (method with α = 0), the method is more efficient than Baseline-DN. However, as previously shown in Fig. 2C, the direct neighborhood cannot bring information for more than 28% of metabolites without literature. Therefore, considering a larger neighborhood can be essential for some overlooked metabolites, and the approach achieves solid performances (area under the curve [AUC] = 0.78) on the validation dataset with α = 0.4. Applying a threshold on *Log*_2_*FC* > 1 results in a true-positive rate (TPR) = 0.35 and a false-positive rate (FPR) = 0.05. Using *LogOdds* as predictor, the method achieved slightly lower performances (AUC = 0.76), with TPR = 0.22 and FPR = 0.04 when applying a threshold on *LogOdds* > 2. Beyond the validation, *LogOdds* is more robust to outlier contributions than *Log*_2_*FC*, and when examining predictions, they should be considered together as complementary indicators of significance and effect size. These results suggest that the prior built from the neighboring literature alone holds relevant information about the biomedical context of metabolites and could be efficient to drive predictions for rarely mentioned compounds. To evaluate the performances of predictions based on the posterior distribution and the behavior of the method on challenging cases, a supplementary analysis was conducted using simulated overlooked metabolites in Supplementary S4.4. Finally, as mentioned in the Method summary, the metabolic network was pruned from spurious connections using an atom-mapping procedure (see Supplementary S1.1). This results in a compound graph, built by linking 2 compounds when they share at least 1 carbon and have a substrate–product relationship in at least 1 reaction. The impact of the carbon skeleton graph on the predictions is evaluated in Supplementary S4.5.

### Suggesting relations with diseases for overlooked metabolites

In the FORUM KG, 80% of the significant associations with biomedical concepts are observed for the 20% of compounds with more than 100 annotated articles. This manifestation of the Pareto principle [24] reflects the need for additional knowledge for compounds that are less frequently mentioned. Therefore, in this analysis, we applied the proposed method on all metabolites in the human metabolic network with fewer than 100 annotated articles (see Table 1). According to the experiments on the validation dataset (see previous section *Evaluation of the prior computation*), we applied a threshold on *LogOdds* > 2 and *Log*_2_*FC* > 1. Predictions for which the prior was biased toward 1 dominant contributor and thus failed to capture the neighborhood literature were excluded by filtering the diagnostic indicator *Entropy* > 1. *Entropy* is the Shannon entropy computed on the contributors’ weights in the prior: the more contributors with balanced weights, the higher the entropy. (See details in Method and Supplementary S1.3.)

**Table 1:** Summary table of the number of disease-related MeSH predicted for metabolites in the network with fewer than 100 annotated articles. The results are separated between the 2 major scenarios: (1) metabolites without literature and (2) metabolites poorly described in the literature (<100 articles). In the second case, results are also arranged according to whether the metabolite already co-mentions the MeSH (co-mention column). Only predictions with *LogOdds* > 2, *Log*_2_*FC* > 1, and *Entropy* > 1 are considered. For the 1,863 predictions where the metabolite co-mentions the MeSH, 938 ($\approx 50\%$) are also retrieved using a right-tailed Fisher exact test (BH correction and *q* < 0.05). Only 793 metabolites among the 1,679 without literature and 254 among those with literature have significant results according to the used thresholds.

	No. metabolites	Co-mention	No. predictions
**Metabolites without literature**	793	No	26,436
**Metabolites with few articles (<100 articles)**	254	No	7,286
		Yes	1,863

In total, 1,863 predictions correspond to relations that are not novel, since they are already supported by 1 or several publications in the literature (co-mention:yes in Table 1). However, by reevaluating them using the same workflow as in FORUM [6] (a standard overrepresentation analysis [ORA] using a right-tailed Fisher exact test, Benjamini–Hochberg (BH) correction, and threshold on *q* ≤ 0.05), we found that $\approx 50\%$ of these associations (925) would not have been highlighted. While only a few articles support these relationships and half of them were discarded by a standard ORA, the method showed their consistency with the literature of metabolic neighbors. A total of 7,286 novel relations have also been suggested with disease-related MeSH, without having been mentioned in their literature already (co-mention:no). Finally, for 793 metabolites without literature, 26,436 relations have been suggested only by exploiting the neighborhood literature. All the results are available on the FORUM ftp server (see [22]), filling a gap when it comes to the interpretation of signatures with these overlooked metabolites.

### Case study

In this section, we will describe the behavior and benefits of the method through 2 test cases. As mentioned in the previous section *Method and Data Description*, hydroxytyrosol is an example of a metabolite without literature (1) and 5α-androstane-3,17-dione of a metabolite with only a few annotated articles (2) and with a weakly supported association.

#### Hydroxytyrosol and its potential link with Parkinson’s disease

**Figure 4:**
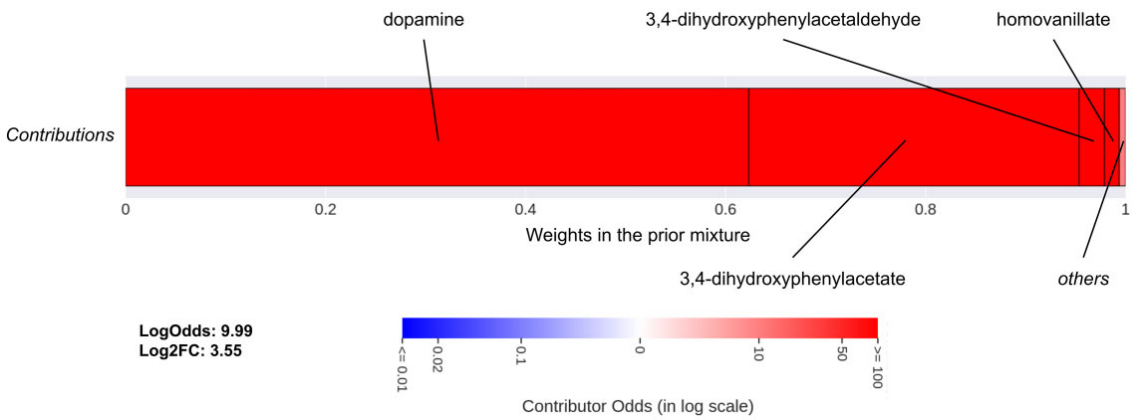
Profile of the contributors for the association between hydroxytyrosol and Parkinson’s disease. This shows the repartition of the literature received by hydroxytyrosol from its neighborhood to build its prior. Contributors are organized in blocks by increasing weights in the prior mixture (*w_i, k_*), from left to right. The weights also give the width of the block. The color of each block associated with a contributor depends on its individual *LogOdds*, from blue to red, for *negative* (less likely) to *positive* (more likely) contributions, respectively. Weights and *LogOdds* are also detailed in Supplementary Table S2.

Hydroxytyrosol is a metabolite that is known for its antioxidant properties [25] and mentioned by 856 publications in FORUM. However, its literature will only serve as ground truth, and hydroxytyrosol will be considered a metabolite without literature in this analysis. Consequently, the predictions are solely derived from the neighboring literature (*f_prior_*). The top 10 predictions ranked by *LogOdds* are presented in Supplementary Table S1. Parkinson’s disease is the most suggested disease, followed by broader descriptors also related to neurodegenerative disorders. This suggestion is mainly driven by the literature of close metabolic neighbors: dopamine and 3,4-dihydroxyphenylacetate (Fig. 4). Both compounds’ literature frequently mention Parkinson’s disease (Supplementary Table S2), suggesting that hydroxytyrosol may also be related to this disease. Other contributors such as 3.4-dihydroxyphenylacetaldehyde or homovanillate also seem to be related to the pathology but only contribute $\approx 5\%$ to the prior as they are more distant neighbors or have less literature. In the actual literature of hydroxytyrosol, 2 articles [26, 27] explicitly discuss its therapeutic properties on Parkinson’s disease.

#### Highlighting the role of 5α-androstane-3,17-dione in polycystic ovary syndrome

**Figure 5:**
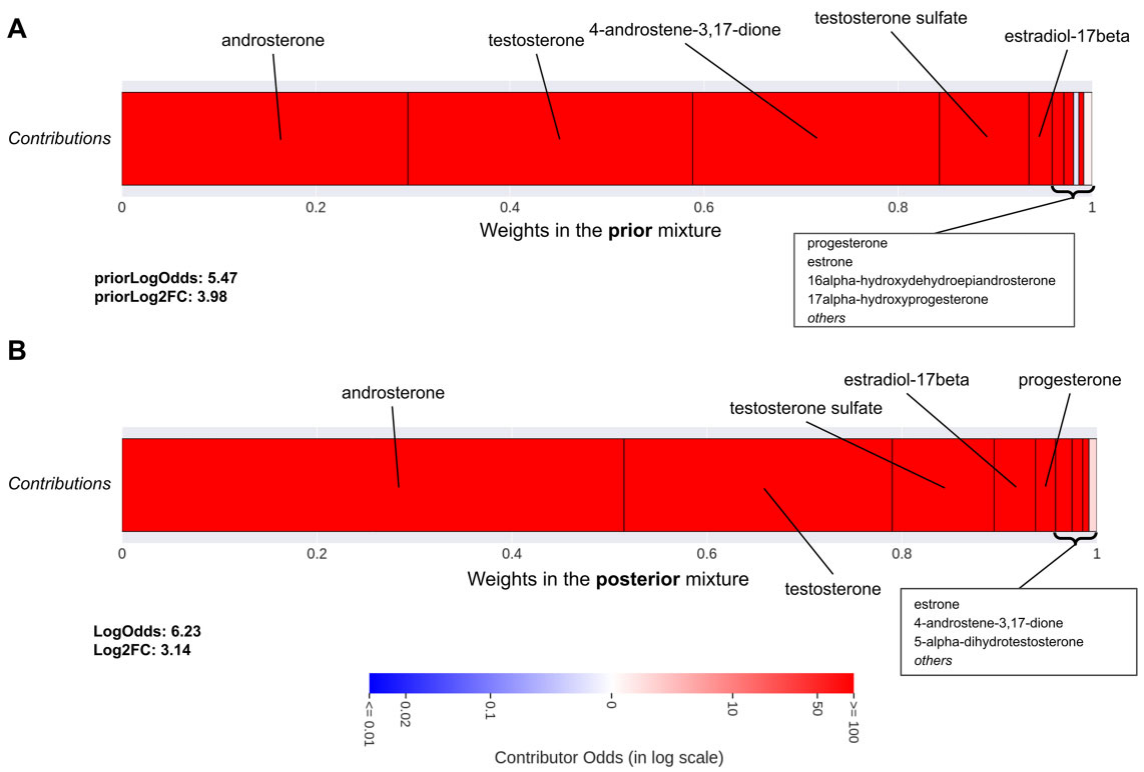
Profile of the contributors for the association between 5α-androstane-3,17-dione and polycystic ovary syndrome in the prior mixture (A) and in the posterior mixture (B). Contributors are organized in blocks by increasing weights in the mixture from left to right, and the weights also give the width of the block. The color of each block associated with a contributor depends on its individual *LogOdds*, from blue to red, for *negative* (less likely) to *positive* (more likely) contributions, respectively. Details in Supplementary Table S4.

Since 82 articles are available for 5α-androstane-3,17-dione (5-αA), the predictions are derived from both its literature and that of its metabolic neighborhood. The top 25 predictions ranked by *LogOdds* are presented in Supplementary Table S3, along with the *P* value from a right-tailed Fisher exact test using the same data for comparison. The highest-ranked associations are supported by several mentions of the compound and by the neighborhood (high *priorLogOdds*). They correspond to mildly interesting predictions as the literature of the compound alone would have been sufficient (significant Fisher *P* value): the neighborhood only strengthens the relation. Instead, we choose to focus on the relation with polycystic ovary syndrome (PCOS), which has a nonsignificant Fisher *P* value and only 1 article supporting the relation [28]. The *priorLogOdds* (5.47) indicates that the literature gathered from the metabolic neighborhood seems highly related to the disease (Fig. 5). While the literature of the compound alone is insufficient to highlight an association with PCOS, the posterior distribution, combining information available from the compound and its neighbors, strongly suggests one (*LogOdds* = 6.23 and *Log*_2_*FC* = 3.14). Androsterone, a direct neighbor of 5-αA through the reaction *3(or 17)-α-hydroxysteroid dehydrogenase*, is the main contributor supporting the prediction (Fig. 5). Additional contributors such as testosterone, testosterone-sulfate, estradiol-17β, and progesterone are more distant metabolically (2–3 reactions) but are also frequently mentioned in this context [29–35]. Also, PCOS is much more frequently mentioned in the literature of 4-androstene-3,17-dione compared to the other metabolites in the neighborhood, making it an outlier among the contributors. Interestingly, its contribution significantly drops in the posterior distribution (see details in Supplementary S4.6 and Supplementary Table S4). A view of the metabolic neighborhood of 5-αA is also presented in Supplementary Fig. S4.

To illustrate the influence of the observations on the posterior distribution, we reevaluated the relation by removing the single co-occurrence between the 5-αA and PCOS. By suppressing this mention, the *LogOdds* drops to 3.67, *Log*_2_*FC* to 2.80, and the weights in the posterior mixture change according to the new observations (see Supplementary Fig. S3). For instance, the weight of androsterone, for which the literature mentions PCOS less frequently than the other top contributors (testosterone, estradiol, etc.), increased while those of the others decreased. More significantly, the weight of 16α-hydroxydehydroepiandrosterone, which is never mentioned with the disease, increases from 0.38% to 3%. By removing this mention, the likelihood of the evidence for each contributor changed, favoring those for whom the disease is less likely to be mentioned in an article. Although the relation is still suggested by the neighborhood, this result shows the impact of the available literature on the predictions.

## Discussion

The interpretation of experimental results in metabolomics requires an intensive dive in the scientific literature. In a biomedical context, researchers often seek studies that mention metabolites from an observed signature, as well as report variations in their concentration in similar phenotypes. However, we have shown that there is a strong imbalance in the distribution of the literature among metabolites, suggesting that this research could be restricted to a subset of the initial metabolic signature. Even if this imbalance is accentuated by technical limitations, it also reflects biological facts: some metabolites are more central and sensitive to phenotypic alterations and would therefore be more frequently reported. Nonetheless, they do not necessarily provide key information when interpreting results, because they do not point to dysregulations on specific pathways. To extend the available data to help interpret results, we propose a method to suggest relations between overlooked metabolites and diseases. Most metabolites (62%) in the network have no literature available, and many cannot be mapped to their corresponding PubChem identifier. It is a common issue when dealing with metabolic networks, as they are initially built for modeling purposes [36]. The absence of annotations also indicates that a compound is not widely described and studied, which may suggest that little literature has actually been lost.

The predictions for metabolites without literature are solely based on their prior distribution, which is built from a mixture of the neighboring literature. We first evaluated the prior alone on a validation dataset (AUC ≈ 0.78) and showed that it holds relevant information about the biomedical context of metabolites. Since the contributors, their weights, and influences in the mixture distribution (more or less likely to mention the disease in an article) are known, the prior is transparent by design. In the example of hydroxytyrosol, the prediction was mainly derived from the literature of dopamine, 3,4-dihydroxyphenylacetaldehyde (DOPAL), and 3,4-dihydroxyphenylacetate (DOPAC), and these studies all frequently mention Parkinson’s disease in their literature. Hydroxytyrosol and its contributors belong to the dopamine degradation pathway [37]. The literature supporting the relation with Parkinson’s disease mainly discusses the production of hydrogen peroxide during dopamine degradation to DOPAL by monoamine oxidase (MAO) enzymes. Since DOPAL is then inactivated into either DOPAC or hydroxytyrosol, the literature that has been propagated by the contributors is metabolically relevant for hydroxytyrosol. Indeed, [38] shows that hydroxytyrosol can induce a negative feedback inhibition on dopamine synthesis, resulting in a decrease of the oxidation rate of dopamine. By indicating which and how neighbors contributed to the predictions, the contribution profile thus adds explainability to the predictions, which we believe is an important quality of the method. It can be quickly established if there was a clear consensus in the neighborhood or if the association was only carried by 1 dominant contributor. In the case of *positive* suggestions, the associated literature of each contributor could be examined to understand the nature of their relation with the disease and assess the consistency of the prediction. Typically, we want to evaluate whether the relationship between the contributors and the disease can indeed be transferred to the target compound, whether it may suggest another, or whether it is irrelevant.

While a consensus is of course preferred (no matter the outcome of the prediction), some contributors may also have divergent literature for a particular disease. To complete the example of hydroxytyrosol, we show the profile of the contributors for the relation between 5-S-cysteinyldopamine (CysDA) and Parkinson’s disease (see Supplementary Fig. S5A). CysDA is the S-conjugate of dopamine and cysteine, and its prior is mainly influenced by the literature of both of these precursors, at 51% and 45%, respectively. While dopamine is strongly related to the disease, cysteine is mentioned much less in this context, and the prior is consequently indecisive (*priorLogOdds* ≈ 0.1). In this case, only the observed literature of CysDA can reduce the uncertainty by updating the prior distribution. In FORUM, 11 articles out of 33 mention CysDA and Parkinson’s disease, which has an important impact on the weights in the posterior mixture in favor of dopamine, which then becomes the dominant contributor (see Supplementary Fig. S5B). Indeed, the posterior weights are proportional to the likelihood of the data according to the prior defined by each contributor. For CysDA, observations clearly suggest that it should be frequently mentioned with Parkinson’s disease, like dopamine, contrary to what is suggested by cysteine. The prediction is highly significant (*LogOdds* = 50.7, *Log*_2_*FC* = 3.87) as the literature for CysDA is very indicative. It is noteworthy that even fewer co-mentions would have already shifted the balance of contributors in favor of dopamine and highlighted this relationship. Supplementary Fig. S5C shows the contributor profiles in the case where only 2 articles had mentioned the disease, which would have been sufficient to highlight the relationship. This emphasizes the sensibility of the method, which may suggest still poorly supported relations but are consistent with the metabolic neighborhood’s literature.

Likewise, the literature linking 5-αA to PCOS is not sufficient in quantity to statistically show a relation. From an expert’s perspective, only 1 qualitative article could be sufficient to justify a relation between a metabolite and a disease. But since the literature and the topics related with metabolomics are broad, highlighting these weakly supported relations could point to relevant paths of interpretation that may have been missed. The relation between 5-αA and PCOS is supported by only 1 article but is highly coherent in the metabolic neighborhood, as androgen metabolism dysfunctions are central in this pathology [39]. As the contributors are widely studied metabolites (androsterone, testosterone, etc.) that also frequently mention the disease in their literature, the prior regarding the relationship is strong and strengthens the observations. We also show that after removing the only supporting article and computing the posterior distribution accordingly, the relation is still suggested, but the *LogOdds* and *Log*_2_*FC* significantly drop. This illustrates the behavior of the method, where the posterior distribution proposes a compromise between the compound’s literature and that of its contributors, giving more weight to those that are the most mentioned and for whom the observations are the most consistent. The neighborhood literature can also help to discard suggestions that are supported by secondary or negligible mentions (see Supplementary S4.7).

With FORUM’s data, relations are evaluated for both disease-specific MeSH and broader descriptors, representative of disease families such as *Neurodegenerative Diseases* (D019636). When there is no consensus among contributors at the level of specific diseases but they all belong to the same category of disorders, more coarse-grained relations could be suggested. Although this increases the redundancy of the results, it makes it easier to grasp the overall biomedical context of some overlooked metabolites.

### Limitations

The most evident limitation of the proposed approach is that the assumption that the literature in the metabolic neighborhood of a metabolite provides relevant prior knowledge on its biomedical context is not always accurate. A short path of reactions can indeed have a major impact on the metabolic activity of compounds, resulting in separate biological pathways and invalidating the hypothesis. For instance, while dopamine is a derivative of tyrosine, the former is a neurotransmitter and the latter a fundamental amino acid. Their biomedical literature therefore covers very different topics, and one would not provide a good *a priori*on the other. Nonetheless, thanks to the transparency of the contributors’ profile, such irrelevant contributions can be identified and the corresponding predictions reevaluated or discarded.

Based solely on the metabolic network, we ignore the regulatory mechanisms of biological pathways and only focus on biochemistry. We therefore assume that all paths of reactions are active and valid when propagating the literature, which is not true and may vary depending on physiological conditions. The predictions could potentially be improved by integrating a regulation layer, but this would add major complexity to the method, and we choose to ignore these constraints by proposing a more general approach. Although reconstructions of the human metabolism like Human1 are constantly improving, they remain incomplete, and some pathways (e.g., lipids [40]) are simplified with missing or artificially created links, mainly for modeling purposes.

With their overflowing literature, overstudied metabolites (amino acids, cholesterol, etc.) can erase the contributions of other neighbors in the construction of a prior. This results in a strong prior that is only fueled by the literature of 1 dominant contributor, and in the case of a metabolite without literature, predictions will therefore be solely based on it. We therefore provide diagnostic indicators like *Entropy, CtbAvgDistance*, and *CtbAvgCorporaSize* (see Supplementary S1.3) to identify these unbalanced priors and flag these predictions. Finally, a part of the biomedical literature of some influential compounds may not be related to their metabolic activity. For instance, ethanol is strongly related to bacterial infections, not as a metabolite but because of its antiseptic properties, which may suggest out-of-context relations by spreading its literature to neighbors. To avoid arbitrary filtering, we allow the user the choice to keep associations with such compounds after review.

## Potential implications

Based on the literature extracted from the FORUM KG, we showed the imbalance in the distribution of the literature related to metabolites. To overcome this bias, we proposed an approach in which we extend the *guilt-by-association* principle in the Bayesian framework. Basically, we use a mixture of the literature of the metabolic neighborhood of a compound to build a prior distribution on the probability that one of its articles would mention a particular disease. The transparency of the contributor’s profile is essential and helps diagnose and explain the predictions by indicating which and how metabolic neighbors have contributed. More than 35,000 relations between metabolites and disease-related MeSH descriptors have been extracted and are available on the FORUM ftp. These relations may help interpret metabolic signatures when no or little information can be found in the literature or databases. In the upcoming release of the FORUM KG, these relations will be integrated as a peripheral graph to supplement the existing metabolite–disease associations and create new paths of hypotheses. In this analysis, we restricted our predictions to a disease-related concept because the metabolic network, although suitable for propagating this type of relationship, would be less reliable for propagating functional relations, for instance. The process is also network dependent, which means that using a different metabolic network (human or other organisms) could result in different suggestions. Nonetheless, the approach could be extended to other entities (genes, proteins) and relations, as long as the related literature is available and the neighborhood of an individual can provide a meaningful prior. Finally, as the literature grows rapidly and metabolic networks become more comprehensive, we hope that this will also improve both the quantity and the quality of the suggestions in the future.

## Methods

### Settings

The approach is metabolite-centric, considering all the available literature for each metabolite and its co-mentions with disease-related MeSH descriptors as input data. Note that each article frequently mentions numerous metabolites, and therefore the literature related to each metabolite, in terms of publications, is not exclusive to that chemical but can be shared with others. We thus call a “mention” the fact that an article mentions a metabolite.

For *M* metabolites in the metabolic network, we note *n_i_* as the total number of mentions of a metabolite *i* and then define $N = \sum _{i=1}^{M} n_i$ as the total number of mentions in the network. Given a specific disease-related MeSH descriptor, we also define *y_i_* as the number of articles co-mentioning the metabolite *i* and the disease, with $m = \sum _{i=1}^{M} y_i$ the total number of mentions involving that disease. Details on the extraction of literature data from the FORUM KG are presented in Supplementary S1.2.

For a metabolite *k* of interest, the random variable *p_k_* denotes the probability that an article mentioning the metabolite *k* also mentions the disease. The aim of the method is to estimate the posterior distribution of *p_k_*, given a prior built from the literature of its metabolic neighborhood. To assess the strength of their relation, *p_k_* is then compared to the expected probability $P = \frac{m}{N}$ that any mentions of a metabolite in the literature also involve the disease. As in the method summary, the scenario in Fig. 1 will be used to illustrate the different steps.

### Estimating the contributions of metabolic neighbors

Based on the assumption that the literature from the metabolic neighborhood of a compound could provide a useful prior on its biomedical context, the first step is to propagate the neighbors’ literature. A random walk with restart (RWR) algorithm (or Personalized PageRank) is used to model a mention, sent by a metabolite *i*, which moves randomly through the edges in the network and reaches another compound *k*. At each step, the mention has a probability α, named the *damping factor*, of continuing the walk and (1 − α) of restarting from the metabolite *i*. The result is a probability vector π_*i*._, indicating the probability that a mention sent by *i* reaches any metabolites *k* in the network, noted π_*i, k*_. The expected number of mentions sent by *i* that reach the compound *k* is then π_*i, k*_*n_i_*. However, in this model, a compound can receive its own mentions (π_*k, k*_ > 0), although only those derived from the neighborhood should be used to build the prior, as the metabolite should not influence itself. A second bias is relative to the set of neighbors for which a metabolite is *allowed* to contribute to their prior. Metabolites with very large corpora (glucose, tryptophan, etc.) can propagate their literature to distant metabolites in the network, even if their probability to reach them is low. In the case of metabolites with a rarely mentioned direct neighborhood, they can predominantly contribute to the prior, although they are not metabolically relevant. This bias is accentuated by the highly skewed distribution of the literature.

To contribute to the prior of *k*, we therefore require that a metabolite *i* should have a probability of reaching *k* (without considering the walks that land on itself) greater than the probability of choosing *k* randomly. The set of metabolites *k* to which *i* is allowed to contribute (namely, the influence neighborhood of *i*, noted *H_i_*) is therefore defined as


(1) \begin{eqnarray*}
k \in H_i \quad \forall k \ne i, \frac{\pi _{i, k}}{(1 - \pi _{i, i})} > \frac{1}{(n - 1)}
\end{eqnarray*}

According to these probabilities, the quantity of literature sent by *i* that reaches *k* is noted as *t_i, k_*, such as


(2) \begin{eqnarray*}
t_{i, k} = \left\lbrace \begin{array}{@{}l@{\quad }l@{}}\frac{\pi _{i, k}}{\sum _{k^{\prime } \in H_i} \pi _{i, k^{\prime }}} n_i\, \text{if}\ k \in H_i.\\ 0, \text{otherwise.} \end{array}\right. \end{eqnarray*}

These aspects are illustrated in Fig. 1B: $\boldsymbol {b}$ does not share any mentions with itself or with $\boldsymbol {z}$, which does not belong to its influence neighborhood in this example. However, $\boldsymbol {a}$ receives $t_{\boldsymbol {b}, \boldsymbol {a}}$ mentions from $\boldsymbol {b}$. Symmetrically, we defined *T_k_* as the set of contributors of *k*, such that *t_i, k_* > 0. Each contributor *i* has a weight *w_i, k_* in the prior of *k*, representing the proportion of literature reaching *k* that was sent by *i*:


(3) \begin{eqnarray*}
w_{i, k} = \frac{t_{i, k}}{\sum _{i^{\prime } \in T_k} t_{i^{\prime }, k}}
\end{eqnarray*}

The weight vector for compound *k* is noted $\boldsymbol {w_k}$. In Fig. 1C, $w_{\boldsymbol {b}, \boldsymbol {a}}$ is the weight of $\boldsymbol {b}$ in the prior of $\boldsymbol {a}$ and as $\boldsymbol {a}$ cannot contribute to itself, $w_{\boldsymbol {a},\boldsymbol {a}} = 0$.

### Mixing neighboring literature to build a prior

The probability *p_i_* that an article mentioning a metabolite also mentions a disease is modeled with a Beta distribution, flexible and suitable for modeling proportions [41]. We assume that *a priori*, any metabolites and diseases are independent concepts in the literature, so that mention of the former does not affect the probability of mentioning the latter and *E*[*p_i_*] = *P*. Under this assumption, for any contributor *i*, the prior distribution of *p_i_* is modeled as a Beta distribution parameterized by mean (μ = *P*) and sample size (ν):


(4a) \begin{eqnarray*}
y_i | n_i, p_i \sim Bin(n_i, p_i) \end{eqnarray*}

(4b) \begin{eqnarray*}
p_i \sim Beta(\alpha ^{(0)}, \beta ^{(0)}) \end{eqnarray*}

(4c) \begin{eqnarray*}
\alpha ^{(0)} = \mu \nu \text{, } \beta ^{(0)} = (1 - \mu )\nu \text{ with } \mu = P
\end{eqnarray*}

The sample size ν is a hyperparameter and controls the variance; the higher ν, the lower the variance: $Var[p_i] = \frac{\mu (1 - \mu )}{1 + \nu }$. More intuitively, ν can be seen as the number of pseudo-observations that support this prior belief. Since μ = *P*, a relationship would not be suggested *a priori*, and the higher ν, the more each contributor *i* would have to bring new evidence (*n_i_*) to change this prior belief [42]. As the Beta distribution is a conjugate prior of the Binomial distribution, the posterior distribution of *p_i_* can also be expressed as a Beta distribution:


(5a) \begin{eqnarray*}
p_i | y_i, n_i \sim Beta(\alpha ^{(1)}_i, \beta ^{(1)}_i) \end{eqnarray*}

(5b) \begin{eqnarray*}
\alpha ^{(1)}_{i} = \alpha ^{(0)} + y_{i} \text{ and } \beta ^{(1)}_{i} = \beta ^{(0)} + (n_i - y_{i}) \end{eqnarray*}

For overlooked neighbors that might bring unreliable contributions, the posterior distribution of *p_i_* acts as a shrinkage procedure, by adjusting the probability distribution toward the overall probability *P* of mentioning the disease. This is illustrated in Fig. 1D: the contributor $\boldsymbol {f}$ has only 2 annotated publications, with 1 mentioning the disease. While the raw estimated probability that $\boldsymbol {f}$ mentions the disease clearly seems overestimated due to its small number of annotated articles, the posterior distribution of $p_{\boldsymbol {f}}$ is more reliable.

As illustrated in Fig. 1E, the prior distribution of *p_k_*, also noted as *f_prior_*, is then defined as a mixture of the distributions $Beta(\alpha ^{(1)}_i, \beta ^{(1)}_i)$ of each contributor, weighted by *w_i, k_*:


(6a) \begin{eqnarray*}
y_k | n_k, p_k \sim Bin(n_k, p_k) \end{eqnarray*}

(6b) \begin{eqnarray*}
p_k \sim \sum _{i \in T_k} w_{i, k} Beta(\alpha ^{(1)}_i, \beta ^{(1)}_i) \end{eqnarray*}

In summary, the parameters α and ν respectively control the average distance to which a metabolite is allowed to contribute to the prior of its neighbors and the strength of the initial prior in the shrinkage procedure. The impact of these parameters on the constructed prior and predictions is discussed in Supplementary S4.3. In the analyses presented in sections *Suggesting relations with diseases for overlooked metabolites* and *Case study*, we set α = 0.4 and ν = 1,000.

### Updating prior and selecting novel associations

For the compound *k*, the final posterior mixture distribution of *p_k_*, also noted as *f_post_* (cf. Fig. 1F), is thus expressed as a mixture of the updated posterior distributions of each contributor, reweighted according to the observed data (*n_k_* and *y_k_*):


(7a) \begin{eqnarray*}
p_k | y_k, n_k \sim \sum _{i \in T_k} W_{i, k} Beta(\alpha ^{(2)}_i, \beta ^{(2)}_i) \end{eqnarray*}

(7b) \begin{eqnarray*}
W_{i, k} = \frac{ w_{i, k} C_{i, k}}{ \sum _{i^{\prime } \in T_k} w_{i^{\prime },k} C_{i^{\prime }, k} }
\end{eqnarray*}

(7c) \begin{eqnarray*}
\text{ with } C_{i, k} = \binom{n_k}{y_{k}} \frac{B(\alpha ^{(2)}_{i}, \beta ^{(2)}_{i})}{B(\alpha ^{(1)}_{i}, \beta ^{(1)}_{i})} \text{ , } \alpha ^{(2)}_{i} = \alpha ^{(1)}_{i} + y_{k}
\end{eqnarray*}

(7d) \begin{eqnarray*}
\text{ and } \beta ^{(2)}_{i} = \beta ^{(1)}_{i} + (n_k - y_{k}) \end{eqnarray*}

*C_i, k_* represents the probability of observing the data (*y_k_*, *n_k_*) of the metabolite *k*, where *p_k_* is drawn from the Beta distribution of the contributor *i* ($Beta(\alpha ^{(1)}_i, \beta ^{(1)}_i)$), as in a Beta-binomial model. Therefore, the posterior weights in the mixture (*W_i, k_*) correspond to the initial weights (*w_i, k_*), reweighted according to the likelihood of the observations from the perspective of the contributor *i*.

From the mixture distribution, we evaluate the probability that *p_k_* ≤ *P*, or the posterior error that an article mentioning the metabolite *k* would mention the disease more frequently than expected, noted *CDF*. We set *q* = 1 − *CDF* and then use the log odds of *q*, such as $LogOdds = log(\frac{q}{1-q})$. Therefore, if *LogOdds* > 0, it is more likely that the metabolite *k* is related to the MeSH than it is not and vice versa. Also, we defined $Log_2FC=log_2(\frac{E[f_{post}]}{P})$. As *LogOdds* can lead to infinite values (if CDF was not precisely computed and approximated to 0), the *Log*_2_*FC* can in turn provide a useful estimator to rank the relations. In turn, *Log*_2_*FC*, being proportional to the mean *E*[*f_post_*], is much more sensitive to outlier contributors than *LogOdds* [43]. When evaluating predictions, *LogOdds* should be considered a measure of significance and *Log*_2_*FC* as a measure of effect size. Finally, *LogOdds* and *Log*_2_*FC* can also be computed independently for each contributor *i* using their associated component in the prior ($Beta(\alpha ^{(1)}_i, \beta ^{(1)}_i)$) and posterior mixture ($Beta(\alpha ^{(2)}_i, \beta ^{(2)}_i)$).

### Different scenarios

For metabolites mentioned in few articles and with literature available in the neighborhood (2), the behavior of the method is exactly as described above. When the compound *k* has no annotated articles (1), only the distribution *f_prior_* is used to compute *LogOdds* and *Log*_2_*FC*. In summary, for metabolites without literature, *LogOdds* and *Log*_2_*FC* are derived from *f_prior_*, while for metabolites with literature, they are obtained from *f_post_*. For the latter, *priorLogOdds* and *priorLog*_2_*FC* are computed from the prior distribution *f_prior_* and aim to represent the belief of the metabolic neighborhood, without the influence of the compound’s literature.

There may be no literature available in the neighborhood of some metabolites. In this case, the prior distribution is simply defined by *Beta*(α^(0)^, β^(0)^), and then the posterior distribution is $Beta(\alpha ^{(1)}_k, \beta ^{(1)}_k)$. In the worst case, when no literature is available for the metabolite and its neighborhood, the basic distribution *Beta*(α^(0)^, β^(0)^) is used, but predictions are automatically discarded.

Since the construction of the prior from the neighborhood’s literature is critical in the proposed method, several diagnostic values are also reported to judge its consistency. Those additional indicators are detailed in Supplementary S1.3.

## Availability of Source Code and Requirements

- Project name: Forum-LiteraturePropagation
- Project homepage: https://github.com/eMetaboHUB/Forum-LiteraturePropagation
- Operating system(s): Platform independent
- Programming language: Python, bash script
- Other requirements: Python 3.7, Pip, Conda
- License: CeCILL 2.1
- RRID: SCR_023874

## Supplementary Material

giad065_GIGA-D-23-00014_Original_Submission

giad065_GIGA-D-23-00014_Revision_1

giad065_GIGA-D-23-00014_Revision_2

giad065_Response_to_Reviewer_Comments_Original_Submission

giad065_Response_to_Reviewer_Comments_Revision_1

giad065_Reviewer_1_Report_Original_Submission Biswapriya Misra -- 2/25/2023 Reviewed

giad065_Reviewer_2_Report_Original_Submission Tara Eicher, M.S. -- 3/3/2023 Reviewed

giad065_Reviewer_2_Report_Revision_1 Tara Eicher, M.S. -- 6/22/2023 Reviewed

giad065_Reviewer_3_Report_Original_Submission Brian DeFelice -- 3/15/2023 Reviewed

giad065_Reviewer_3_Report_Revision_1 Brian DeFelice -- 6/22/2023 Reviewed

giad065_Supplemental_File

## Data Availability

The dataset(s) supporting the results of this article are available on the GitHub repository [22]. Snapshots of our code and other data further supporting this work are openly available in the *GigaScience* repository, GigaDB [44].

## Additional Files

**Supplementary Fig. S1**. Example of the galactokinase reaction in the reconstruction process of the carbon skeleton graph: the galactokinase is an enzyme that catalyzes the phosphorylation of galactose into galactose-1-phosphate. Colored circles describe the carbons shared between each participant of the reaction; their number is also indicated. The blue square shows the phosphate transferred from the ATP to the galactose. There is no carbon shared between galactose and ADP or between ATP and galactose-1-phosphate.

**Supplementary Fig. S2**. Detailed workflow diagram of the presented analysis. The left part of the diagram illustrates the process of extracting co-mention data between PubChem compounds and disease-related MeSH descriptors from the FORUM KG. Additionally, the upper part outlines the construction of the carbon skeleton graph (CSG) from the Human1 metabolic network (v1.7) and its integration into the FORUM KG, facilitating the linkage of metabolic species with their co-mention data. The step labeled “Metabolites’ Influence Matrix Step” denotes the computation of probabilities *π_i,k_* using a random walk with restart algorithm on the resulting CSG (refer to the Method section for further details). Lastly, the lower part of the diagram demonstrates the combination of these intermediary data elements to compute the predictions.

**Supplementary Fig. S3**. Profile of the contributors for the association between 5-αA and PCOS without the single co-occurrence (PMID 8855823). Contributors are organized in blocks from left to right by increasing contributions. The contributions correspond to the weight of each contributor in the posterior mixture (*W_i, k_*) and give the width of the block. The color of each block associated with a contributor depends on its individual *LogOdds*, from blue to red, for negative (less likely) to positive (more likely) contributions, respectively. Weights and *LogOdds* are also detailed in Supplementary Table S5.

**Supplementary Fig. S4**. View of the metabolic neighborhood of 5-αA (in red). Main contributors of the relation with PCOS are highlighted in blue.

**Supplementary Fig. S5**. Profile of the contributors for the association between 5-S-cysteinyldopamine and Parkinson’s disease. The profile of the contributors from the prior distribution is shown in A and from the posterior distribution in B, with actual literature data: 11 supporting articles out of 33. C is the profile of the contributors with only 2 co-occurrences. It represents the minimal number of co-occurrences necessary to shift the balance of contributors and highlight the relationship.

**Supplementary Fig. S6**. Boxplot of the average distance of the contributors, weighted by *w_i, k_*, using different damping factors *a*. The red dotted line connects the median of each boxplot, and the black horizontal line is a threshold at an average distance of 2 reactions.

**Supplementary Fig. S7**. Distribution of the contributors’ weights in the prior mixtures *w_i, k_*, at a distance of n reactions, for several damping factors *a*. The red dotted line connects the medians, and the blue dots represent the maximal outliers.

**Supplementary Fig. S8**. Evaluation of the true-positive rate (TPR), false-positive rate (FPR), and precision on the validation dataset obtained with a threshold on *LogOdds* >2, using different combinations of hyperparameters α and ν.

**Supplementary Fig. S9**. Average receiver operating characteristic (ROC) curves per tested sample sizes for the method set with α = 0.4, ν = 1,000, and Baseline-DN + Cpd. In A, performances are evaluated on datasets of simulated overlooked metabolites, with increasing sample size: 10, 50, and 100. In B, only the Hard cases have been retained to evaluate the performances of the method against Baseline-DN + Cpd.

**Supplementary Fig. S10**. ROC curves using *Log_2_FC* as predictor with the carbon skeleton graph (CSG network) or the original Human1 metabolic network. The AUCs are respectively 0.78 and 0.74. The red dotted line corresponds to random strategies.

**Supplementary Fig. S11**. Detailed prior mixture of the top 10 contributors for the relation between 5-αA and PCOS. The individual distributions of each contributor in the mixture, along with the parameters of the associated Beta distribution, are indicated.

**Supplementary Fig. S12**. Prior (blue) and posterior distributions obtained with (red) and without (green) the co-mention for the relation between 5-αA and PCOS.

**Supplementary Fig. S13**. Profile of the contributors for the association between 5-αA and meningioma in the prior mixture (A) and in the posterior mixture (B). Contributors are organized in blocks by increasing weights in the mixture from left to right, and the weights also give the width of the block. The color of each block associated with a contributor depends on its individual *LogOdds*, from blue to red, for negative (less likely) to positive (more likely) contributions, respectively.

**Supplementary Table S1**. Top 10 disease-related MeSH suggested for hydroxytyrosol, ranked by *LogOdds*.

**Supplementary Table S2**. The table describes different properties of the contributors for the association between hydroxytyrosol and Parkinson’s disease: corpora corresponds to the total number of mentions associated with the compound; cooc is the number of co-occurring mentions with the disease; *LogOdds* indicates the individual *LogOdds* of the contributors in the prior mixture, same for *Log_2_FC*; weights indicates the weight of each contributor in the prior mixture. The values in others correspond to the median for the remaining contributors.

**Supplementary Table S3**. Top 25 disease-related MeSH predicted for 5-αA, ranked by *LogOdds*. The cooc column indicates the number of co-occurring mentions with the disease. *P* value Fisher refers to the *P* value obtained with an overrepresentation analysis (Fisher right-tailed exact test) using the same literature data as used for the predictions (see Supplementary S1.2).

**Supplementary Table S4**. The table describes different properties of the contributors for the association between 5-αA and PCOS: corpora corresponds to the total number of mentions associated with the compound; cooc is the number of co-occurring mentions with the disease; prior weights indicates the weight of each contributor in the prior mixture; posterior weights indicates the weight of each contributor in the posterior mixture; *LogOdds* indicates the individual *LogOdds* of the contributors in the posterior mixture, same for *Log_2_FC*. The values in others correspond to the median for the remaining contributors. As in Figure 5, contributors are ordered by posterior weights.

**Supplementary Table S5**. The table describes different properties of the contributors for the association between 5-αA and PCOS without the single co-mention: corpora corresponds to the total number of mentions associated with the compound; cooc is the number of co-occurring mentions with the disease; *LogOdds* indicates the individual *LogOdds* of the contributors in the posterior mixture, same for *Log_2_FC*; weights indicates the weight of each contributor in the posterior mixture. The values in others correspond to the median for the remaining contributors. As in Figure 5, contributors are ordered by *posterior weights*.

**Supplementary Table S6**. Average AUC obtained on the predictions with the proposed method and Baseline-DN + Cpd, by increasing sample sizes, on the full validation datasets (*Full*) and only on the *Hard* cases.

**Supplementary Table S7**. Average TPR on the predictions obtained with the proposed method and Baseline-DN + Cpd on Hard cases for an FPR fixed at 0.05 and by increasing sample sizes.

## Abbreviations

CysDA: cysteinyldopamine; DOPAL: 3,4-dihydroxyphenylace-taldehyde; DOPAC: 3,4-dihydroxyphenylacetate; FPR: false-positive rate; KG: knowledge graph; ORA: overrepresentation analysis; PCOS: polycystic ovary syndrome; ROC: receiver operating characteristic; RWR: random walk with restart; TPR: true-positive rate.
