# Supplementary material for: Suggesting disease associations for overlooked metabolites using literature from metabolic neighbors
Source: Gigascience. 2023 Sep 15;12:giad065. doi: 10.1093/gigascience/giad065 (PMC10502579; doi:10.1093/gigascience/giad065)
Supplement: giad065_GIGA-D-23-00014_Revision_1 [file giad065_giga-d-23-00014_revision_1.pdf]

GigaScience

Suggesting disease associations for overlooked metabolites using literature from metabolic neighbours

--Manuscript Draft--

**Manuscript Number:**	GIGA-D-23-00014R1
**Full Title:**	Suggesting disease associations for overlooked metabolites using literature from metabolic neighbours
**Article Type:**	Research
**Funding Information:**	H2020 Societal Challenges (825489) — Not applicable<br>INRA SDN — Mr Franck Giacomoni<br>Agence Nationale de la Recherche (11-INBS-0010) — Not applicable
**Abstract:**	In human health research, metabolic signatures extracted from metabolomics data are a strong added value for stratifying patients and identifying biomarkers. Nevertheless, one of the main challenges is to interpret and relate these lists of discriminant metabolites to pathological mechanisms. This task requires experts to combine their knowledge with information extracted from databases and the scientific literature. However, we show that the vast majority of compounds (> 99%) in the PubChem database lack annotated literature. This dearth of available information can have a direct impact on the interpretation of metabolic signatures, which is often restricted to a subset of significant metabolites. To suggest potential pathological phenotypes related to overlooked metabolites which lack of annotated literature, we extend the 'guilt by association' principle to literature information by using a Bayesian framework. The underlying assumption is that the literature associated with the metabolic neighbours of a compound can provide valuable insights, or an a priori, into its biomedical context. The metabolic neighbourhood of a compound can be defined from a metabolic network and correspond to metabolites to which it is connected through biochemical reactions. With the proposed approach, we suggest more than 35,000 associations between 1,047 overlooked metabolites and 3,288 diseases (or disease families). All these newly inferred associations are freely available on the FORUM ftp server (See information at https://github.com/eMetaboHUB/Forum-LiteraturePropagation.).
**Corresponding Author:**	Clément Frainay<br>UMR1331: Toxalim<br>Toulouse, FRANCE
**Corresponding Author Secondary Information:**	
**Corresponding Author's Institution:**	UMR1331: Toxalim
**Corresponding Author's Secondary Institution:**	
**First Author:**	Maxime Delmas
**First Author Secondary Information:**	
**Order of Authors:**	Maxime Delmas<br>Olivier Filangi<br>Christophe Duperier<br>Nils Paulhe<br>Florence Vinson<br>Pablo Rodriguez-Mier<br>Franck Giacomoni<br>Fabien Jourdan

Powered by Editorial Manager® and ProduXion Manager® from Aries Systems Corporation

	Clément Frainay
**Order of Authors Secondary Information:**	
**Response to Reviewers:**	Response to Reviewers<br><br>Reviewer #1:<br>Manuscript Number: GIGA-D-23-00014, entitled, " Suggesting disease associations for overlooked metabolites using literature from metabolic neighbours", and submitted to the journal: GigaScience, applied 'guilt by association' principle to literature information for "understudied metabolites" by using a Bayesian framework. It is an interesting manuscript, an active area of research and would have an interest in the metabolomics research community. However, this reviewer would like to help improve the manuscript and scope of the work with the following suggestions:<br><br><br>1.1 A list/ DB of all such "overlooked metabolites" and their chemical class distribution/ ChemRICH sort of enrichment would help the readers capture the correct information.<br><br>The authors thank the reviewer for bringing this suggestion. The complete list of overlooked metabolites (2113 species) have been added on the GitHub repository and in the FTP server. Since overlooked metabolites can have limited annotations in standard chemical ontologies such as Chebi or MeSH, we decided to use ClassyFire. ClassyFire provides an automatic hierarchical classification of molecules based on structural descriptors such as inchiKey identifiers. We managed to obtain an InchiKey for 1180 (approximately 56%) out of the total 2113 metabolites considered as overlooked in the metabolic network using their annotation in MetaNetX. Subsequently, we analyzed the distribution of the superclass to which these metabolites are classified by ClassyFire. SuperClasses are generic categories of compounds that we can use to get an estimation of the composition of chemical families in the set of overlooked metabolites for this metabolic network. From this sample, it can be estimated that the majority of metabolites considered as overlooked in this metabolic network are actually "Lipids and lipid-like molecules" (e.g: Fatty Acyls, Sphingolipids, etc.), a class with a strong compositional complexity. However, this observation is based on a limited sampling of metabolites within a specific metabolic network. As a result, this subset is unlikely to be representative and while it could give some insights, we argue that it could lead to misleading interpretations and decided to not add this directly in the article.<br><br>A figure of the distribution of the chemical superclass obtained with ClassyFire can be found in the attached document.<br><br><br>1.2 How did/ would the tool perform with "very well known metabolites" for example say, phenylalanine or proline or citric acid ?<br><br>We thank the reviewer for this interesting remark. The behaviour of the method for “very well known metabolites”, as opposed to overlooked metabolites, is quite straightforward in the Bayesian settings. The impact of the prior on the predictions will vanish as the literature of the targeted compound increases. The LogOdds estimator then tends to infinity, while Log2FC tends to its exact value when estimated only from the literature of the compound. For instance, among the 278,277 articles discussing the glucose in FORUM, 24.839 co-mentioned the Diabetes type 2 MeSH descriptor. The prior and posterior distributions obtained for this relationship are presented below. The posterior distribution is solely driven by the literature of the glucose which, being much larger than that of its contributors, completely erases the information brought by the prior. The distributions of the contributors in the posterior mixture are therefore centred around the co-mention frequency of the glucose and Diabetes type 2 (≈ 0.089). Thus, although the proposed approach can be applied to these well-known metabolites, the predictions are insensitive to the built prior which is nevertheless at the core of this method. In this case, the relationships would be as well evaluated with a classic over-representation analysis.<br><br>In addition to the aforementioned extreme example, a similar phenomenon can be observed through an example proposed by the reviewer: Phenylalanine (specie id

Powered by Editorial Manager® and ProduXion Manager® from Aries Systems Corporation

M_m02724c) and the MeSH descriptor Phenylketonurias or PKU (D010661). PKU represents a group of disorders caused by a deficiency in the production of phenylalanine hydroxylase, and for which the dosage of phenylalanine is the standard diagnostic method. Again, the posterior distribution eliminates any information from the prior and is centred around 0.0107, which is the expected probability that an article mentioning phenylalanine also mentions the disease. Indeed, out of the 28.507 articles mentioning Phenylalanine, 3.045 are annotated with the MeSH term PKU.

Figures of the Prior and posterior distributions of this two examples can be found in the attached document.

1.3 How does one check for "literature / reporting biases" for the highly reported vs lowly reported metabolites in the manuscripts ?

From our understanding, hoping we interpret correctly reviewer comment, this check would be related to the retrieval of metabolites' mentioning articles. We hope that the following information can answer your question:
There are several ways one can access the literature of metabolites described in this manuscript. First, all the data are publicly available in the git repository https://github.com/eMetaboHUB/Forum-LiteraturePropagation where uncompress_species_pmids_Human1_1.7.csv contains the number of annotated articles for each of the 2704 species in the pruned version of Human1 metabolic network. If one desires to recover the list of PubMed identifiers behind these frequency values, the FORUM KG (https://forum-webapp.semantic-metabolomics.fr) is the most direct way of recovering the original set of articles mentioning a metabolite. However, as this extraction requires querying the SPARQL endpoint, which we acknowledge is difficult for non-familiar users, we would recommend accessing it individually for each compound from their PubChem page or directly on PubMed.

1.4 Does this approach distinguish for targeted vs untargeted metabolomics paper based hits ?

We thank the reviewer for this interesting remark. Although we could increase the confidence of hits from targeted analyses compared to untargeted using some weighting policies (or using Metabolomics Standard Initiative classification for metabolite identification), the main challenge would lie in accurately extracting this information. In fact, articles related to metabolomics analyses are not yet indexed in PubMed with a precise MeSH term to distinguish the two types of approaches. Determining this from the title or abstract would also require building a classification model for which training data are not available. More generally, proposing a different weighting for the contribution of each article according to different factors (type of analysis, date, etc.), so that they are not all considered equivalently, is indeed an interesting perspective for future works.

1.5 "Overlooked metabolites" need to be defined well, upfront for clarity.

We are grateful to the reviewer for pointing out this lack of clarity. We propose to modify the end of the first paragraph of the Method section: "In this study we define a set of overlooked compounds as compounds with less than 100 retrieved mentioning article, which correspond to orders of magnitude below 4,799, the mean number of retrieved articles per compound (when any), and is close to the median number of articles, 172. It is worth mentioning that such threshold serves solely as a prioritization criterion, since the method applicability is not restricted to a given range of mentioning corpus sizes (although its relevance is less obvious when a sufficient corpus is already available)."

1.6 large fraction of metabolites are rarely or never mentioned in the literature: What is a good estimate from the authors? A numerical value would be informative here.

While our results from the metabolic network clearly suggest that a large fraction of

metabolites are overlooked, we argue that this information, although reflecting a reality in the field, cannot be used to propose a reliable estimate. This estimator would be biased by various factors and in the first place, the lack of external identifiers (e.g. CID) that connect to the literature. Additionally, the purpose of the metabolic network is not to provide an exhaustive map of the metabolism and some parts (e.g lipid metabolism are often reduced to generic classes). Nonetheless, our estimate based on the whole PubChem database seems more reliable and we decide to put the emphasis on it in the abstract to provide a numerical value. We therefore reworked the abstract by adding the following sentence: “However, we show that the vast majority of compounds (> 99\%) in the PubChem database lack annotated literature. This dearth of available information can have a direct impact on the interpretation of metabolic signatures, which is often restricted to a subset of significant metabolites}.”

1.7 Too many terms used does not help: overlooked metabolites vs. understudied metabolites and so on. Please use a singular term for consistency.

We thank the reviewer for helping us improve the readability of the manuscript. We replaced every mention of “understudied” with “overlooked”.

1.8 Method and data description section is too wordy, need to be shortened and need to use mathematical expressions whenever applicable.

We appreciate the feedback regarding the "Method and data description" section of our article and we acknowledge that this section may be too wordy and lacking in mathematical expressions. We made some improvements and tried as much as possible to reduce the size of this section.
We made this choice given the potential readership of the work. We anticipate that some readers wishing to use the provided associations to interpret their results, may not have a strong mathematical background. Therefore, while the use of mathematical expressions would shorten the section, it could also be a barrier to its understanding and discourage some readers. We have strived to make our methodology as accessible as possible by providing two descriptions, which we believe will complement each other. Our primary focus in the "Method and Data description" section is to provide an intuitive and concise overview of the main steps of our approach, avoiding the use of mathematical expressions.
Simple expressions have been added to this section according to the various reviewers' comments in order to remove potential ambiguities. In addition, a complete description with all the mathematical details is provided at the end of the manuscript in the method section for the interested readers.

Reviewer #2:
Overall Notes
This work is innovative and will provide an important contribution to the computational metabolomics field. The experiments and methodology are well-designed and executed, and the software is also well-documented. That being said, the structure and writing of the manuscript needs to be reworked. There are several areas of the text where descriptions are unclear, detailed below. Some of the text is also out of order, e.g. weights are shown in a figure before they are defined, and TPR and FPR are reported without describing the dataset. Finally, there are several Supplementary experiments that are never mentioned in the main text. At least a brief description of these should be given in the main text and then the Supplementary referenced.

2.1 Abstract
Some of the language used here is difficult to read or unclear. In particular:
2.1.1I believe you mean to say that signatures… "have a strong added value", not "are

a strong-added value".

We corrected this in the manuscript.

2.1.2"we extend the 'guilt by association' principle to literature information by using a Bayesian framework". This is vague. Instead, briefly explain how you use a Bayesian framework to determine guilt by association.

We reworked the abstract and specifically added the following sentence to briefly illustrate the intuition behind the prior and the Bayesian framework in the context of the guilt by association principle: “The underlying assumption is that the literature associated with the metabolic neighbours of a compound can provide valuable insights, or an a priori, into its biomedical context.”

2.1.3 "1,047 overlooked metabolites". Do you mean metabolites not in the literature?

Not exactly, we meant metabolites which are rarely mentioned in articles (< 100 annotated articles), so they almost never mentioned in the literature. As this notion of “overlooked” metabolites is key in this article, it has also been clarified in section “Method and data description” according to the Reviewer 1 comments.

2.1.4 Your method uses knowledge about metabolic interactions/reactions to generate the graph, but this is not mentioned at all in the abstract. The abstract should explain that this knowledge is being used and describe how it is complementary to the literature.

Following the previous comments and the addition of the underlying hypothesis in the abstract, we also decided to add the following sentence to emphasize the role of the metabolic network in defining the structure of the graph used to propagate information: “The metabolic neighbourhood of a compound can be defined from a metabolic network and correspond to metabolites to which it is connected through biochemical reactions.”

2.2 Background

2.2.1 it is irrelevant to mention exponential growth. This detracts from the main point, which is the imbalanced knowledge distribution.

We removed this part of the sentence from the manuscript.

2.2.2 "This topic has received much attention for genes and proteins…" Can you provide some citations?

The references related to this statement were provided in the next sentence (“Consequently, [...] gene annotations in databases”). According to this reviewer comment, we decided to move them upstream.

2.2.3 “has an impact on the quantity and quality of gene annotations in databases" - in what way? Can you be more specific?

We wanted to highlight that the skewed distribution of the number of bibliographic references across genes is also reflected in the distribution of functional annotations in databases, such as Gene Ontology. See for instance between TP53 and ANKRD52. As it doesn’t bring much more details for the rest of the article and could distract the reader, we decided to remove this sentence.

2.2.4 The first sentence of the second paragraph can be removed.

Powered by Editorial Manager® and ProduXion Manager® from Aries Systems Corporation

	This sentence has been removed according to the reviewer's suggestion.<br><br>2.2.5 You discuss the issue of inaccurate identification as being related to the number of articles mentioning a compound. I feel that these are two separate issues. The first is related to identification, and the second is related to discussion of identified metabolites in the literature. The section regarding identification should be removed.<br><br>This section has been removed according to the reviewer's suggestion.<br><br>2.2.6 "Guilt by association principle", not hypothesis.<br><br>This has been corrected.<br><br>2.2.7 "The method returns several predictors to evaluate whether a significant proportion of the articles mentioning a metabolite would also mention a disease." Do you mean to say that the predictors predict whether or not the metabolite is related to the disease?<br><br>Indeed, by indicating whether a significant proportion of the articles mentioning a metabolite would also mention a disease, these predictors are meant to highlight a potential relation. We acknowledge that this could be expressed more explicitly in the background section, leaving this interpretation for the methodology section. We reworked this sentence accordingly.<br><br>2.2.8 You mention that you used FORUM Knowledge Graph to obtain your metabolite-disease associations. What about the metabolic neighborhoods? You should explain where these were obtained.<br><br>The metabolic neighbourhoods are defined from the Human 1 (v1.7) metabolic network, which was also pruned from spurious connections using an atom-mapping procedure. While we keep the details apart from the main text, we reworked the following sentence: “Metabolic neighbourhoods were defined from the Human1 metabolic network and co-mention data between metabolites and diseases were extracted from the FORUM Knowledge Graph (KG)”.<br>The details of the pre-processing step on the metabolic network and its implication of the results are detailed in Supplementary materials (S1.1, S4.5) and referenced in Method and Data Description.<br><br>2.3 Method and Data Description<br>2.3.1 How do you define "rarely mentioned"? Is there a cutoff criteria used?<br><br>We thank the reviewer for this interesting remark, which has also been highlighted by the other reviewers. We believe that the modifications applied to the first paragraph of the method section should clarify what we meant by "overlooked" or "rarely" mentioned metabolites, both conceptually and practically.<br><br>2.3.2 Does "amount of literature" mean number of articles?<br><br>Yes, we reformulated the formulation "amount of literature" everywhere in the article to bring clarity, as suggested by this reviewer.<br><br>2.3.3 How is "far distant" defined? It seems that you mean to say that one metabolite's influence on another decreases as the number of reactions separating them increases. Is this correct?<br>We thank the reviewer for pointing out this lack of clarity. Indeed, we make the assumption that the influence of a metabolite on another decreases as the number of reactions separating them increases. In order to consider all the potential paths

Powered by Editorial Manager® and ProduXion Manager® from Aries Systems Corporation

connecting two metabolites in the network, we use the stationary probabilities from random walks starting from the former and reaching the latter as a measure of this distance.
However, when we referred to “far distant” metabolites in the sentence: “We impose that a metabolite can't influence its own prior or the prior of far distant metabolites.”, we inaccurately referred to metabolites whose probability of being reached during the random walk are below a predefined threshold. This concerns, for instance, metabolites that belong to different regions of the metabolic network. This constraint prevents influential metabolites (tryptophan, glucose, etc.) from sharing the articles mentioning them with metabolites that are unlikely to be involved in the regulation of common metabolic pathways.

Since this detail is explained thoroughly with mathematical expressions in section "Estimating the contributions of metabolic neighbours" in Method for interested readers and is not crucial for the understanding of the approach as a whole, we have chosen to exclude it from the method summary. The Figure 1 has also been updated accordingly.

2.3.4 Show Figure 1 as soon as it is mentioned. This goes for the other figures as well.

Indeed, the initial position of the figure was not ideal to follow the corresponding method in the main text, so we moved it one page closer.

2.3.5 You should explain more about the shrinkage procedure here. It isn't clear what you mean.

We are thankful to the reviewer for helping us to make this paper more clearer, particularly for the shrinkage step which is a key element in the presented approach. While the idea of shrinkage is also used for penalized regression, in this manuscript we refer to its applications in Bayesian settings. In this framework, the posterior mean distribution is shrunk towards the prior mean (μ), resulting in a more reliable estimator than the maximum likelihood estimator (MLE) for low sample sizes. This is illustrated in equations 5a and 5b when we show the posterior distribution of pi. The parameterization of the prior beta distribution involves determining μ, assuming that metabolites and diseases are independent concepts in the literature, and setting the sample size (v) as a hyperparameter to control the strength of the prior.
We decided to modify the corresponding paragraph in the method summary section according to the reviewer’s comments : “This results in a small sample size available to estimate the probability that an article mentioning f also mention the disease, which may lead to unreliable and spurious contributions. To address this, a shrinkage procedure is applied to all contributors, assuming that a priori, mentioning a metabolite in an article does not affect the probability of mentioning a particular disease. In Bayesian settings, a shrinkage estimator integrates information from the prior to readjusted raw estimates, reducing the effect of sampling variations (further details in section Mixing neighbouring literature to build a prior in Methods).“
We also redirect the interested reader to the Method section for the mathematical details.

2.3.6 In Figure 1C, there appears to be a stack of papers in a pink box in both the numerator and the denominator. What does this mean?

We thank the reviewer for pointing out this unclear illustration. This pink box represents the number of papers shared by b that reached the target compound a. This quantity is noted tb,a. In Figure 1.C, we illustrated the simple computation of the weight of b in the prior of a (noted wb,a) as the fraction of articles that reached A (tb,a + tc,a + te,a + tf,a) that was send by b (tb,a). To avoid any other ambiguities in this illustration, we explicitly annotate all the paper box with their corresponding value (e.g; tc,a or wb,a) both in 1.B and 1.C.

2.3.7 "Then, we build the prior distribution for A, by mixing the probability distributions of each contributor (see Figure 1.E) according to their weights estimated in the

previous step (Figure 1.C)". This is the first time you mention weights. You need to describe what the weights correspond to and how they are calculated first.

We apologize for adding this ambiguity. We reworked this section, both at the first mention of the weights and in the highlighted sentence. We also added a more explicit mention of the weights associated with the contributors using simple mathematical expressions: “We refer to b, c, e and f as the contributors to the prior of a. Each contributor has a weight w in the prior of a (e.g wb,a) proportional to its contribution.” We also add a reminder in the following sentence: “Then the prior distribution of a is built as a mixture of the probability distributions of individual contributors (b, c, e and f) as illustrated in Figure 1.E. Recall that the weight of each contributor in the mixture is (w.,a), as estimated in the previous step (see Figure 1.C).”

2.3.8 "Then, we build the prior distribution for A, by mixing the probability distributions of each contributor (see Figure 1.E) according to their weights estimated in the previous step (Figure 1.C)". This sentence is unclear. Please revise.

We reformulate this sentence according to the previous comment. Please, see the previous answer.

2.3.9 "Finally, several diagnostic values such as Entropy allow to assess the composition of the built prior (See Supplementary S1.3)". Either name all of the diagnostic values here or move Entropy to the supplementary and out of the main text.

As suggested by the reviewer, we removed the mention of Entropy in this sentence.

2.3.10 "Entropy evaluates the good balance of contributions in the prior. The more metabolites contribute to the mixture and the more their weights are uniformly distributed, the higher the entropy." This is not a clear explanation. Please explain mathematically what entropy is and what it represents here.

According to the last two comments of this reviewer, we reworked this paragraph. We decided to focus more on the applications and purposes of these diagnostic indicators rather than their formal definitions, which we decided to keep for the supplementary materials for the interested readers. We therefore replace the sentence relative to Entropy to a broader description of the purposes of the diagnostic indicators: “Finally, given its primary role in driving predictions, assessing the composition of the constructed prior is crucial. Essentially, the more contributors to the prior, close to the target compound, with balanced weights, the better it captures the neighbourhood literature and increases the confidence in predictions. To aid in this evaluation, a set of diagnostic indicators is presented in Supplementary S1.3“.

However, as Entropy is the only diagnostic indicator used in the main text (for filtering the predictions), we also added a more formal definition directly where it is mentioned in section “Suggesting relations with diseases for overlooked metabolites”. See comment 2.6.3.

2.4 Analyses: Unbalanced distribution of the literature related to chemical compounds
2.4.1 At the end of the first paragraph, it should be "is cumulatively less than the literature associated with glucose…"

This has been added to the manuscript.

2.4.2 Can metabolites without a CID be found in the metabolic network? If not, then you should not discuss those metabolites with an unannotated CID.

With the exception of the pruning process carried out during the construction of the carbon skeleton graph and described in S1.1, no metabolites were excluded because of a lack of annotations. All metabolites without an annotated CID are conserved in the

Powered by Editorial Manager® and ProduXion Manager® from Aries Systems Corporation

	metabolic network.<br><br>2.5 Analyses: Evaluation of the prior computation<br>2.5.1 "and is set to α = 0 for the direct neighbourhood and α = 0.4 for a larger one". Are these the only two values you consider? If so, why? How large is "larger"? This should also go in the methods section.<br><br>We thank the reviewer for pointing out this lack of clarity. An extensive analysis was actually performed on the impact of the both parameters α and v on the performances and the composition of the prior in S4.3 Damping factor α and theoretical sample size v: benchmark. We evaluated values for α in the set: [0, 0.1, 0.2, 0.3, 0.4, 0.5, 0.6, 0.7, 0.8, 0.9, 0.99] and for v in the set [1, 10, 100, 1000, 10000, 100000 , 1000000]. From the performed analyses, we found that α=0.4 and v=1000 appears to be reasonable setting, both in terms of composition of the built prior and balance between sensibility and precision. Finally, we also argued that there are no global optimal settings and recommend 0 α 0.7 and 1 v 10000. We reworked this paragraph to point to these details in the supplementary materials when referring to the chosen values of α: "We therefore focused on two specific settings: α=0, where solely the direct neighbours contribute to the prior, and α=0.4, where contributions between direct or indirect neighbours are relatively balanced. The impact of the parameter α on the construction of the prior and the Precision-Recall tradeoff was extensively evaluated in Supplementary Material S4.3."<br><br>2.5.2 "All tested approaches outperform Baseline-Freq, showing the benefit of examining the neighbouring literature." This experiment needs to be explained in the main text. How did you define TPR and FPR?<br><br>Indeed, the evaluation results with the ROC curves were not explicitly presented in the main text and we decided to rework this paragraph to add "The evaluation results on the validation dataset for all described approaches are presented in Figure 3.". We also add a description of TPR and FPR in the legend of Figure 3: "A true positive represents an association between a compound and a MeSH term which is both retrieved from the compound's mentioning corpus using Fisher Exact Test, and using methods in which no knowledge of such corpus is available. A false positive is only retrieved from the latter."<br><br>2.6 Analyses: Suggesting relations with diseases for overlooked metabolites<br>2.6.1 "However, by re-evaluating these predictions using a right-tailed Fisher exact Test (BH correction and selecting those with q.value <= 0.05), we found that ≈ 50% of them (925) would not have been found significant". Can you please explain this experiment further?<br><br>We thank the reviewer for pointing out this lack of clarity. With this analysis we wanted to emphasize the proportion of associations that would not have been highlighted by a standard approach, thus without considering the neighbourhood information. The right-tailed fisher exact test is a standard and robust approach for over-representation analysis of associations in the literature that we already applied at large scale in the first version of FORUM. We therefore used this same workflow to test all associations between metabolites and diseases based on their co-mention frequency from the literature available in the metabolic network. We reformulate this paragraph and directly refer to the analysis done in the FORUM article: "However, by re-evaluating them using the same workflow as in FORUM [FORUM 2021] (a standard over-representation analysis (ORA) using right-tailed Fisher exact Test, BH correction and threshold on q.value <= 0.05), we found that ~50% (925) of these associations would not have been highlighted."<br><br>2.6.2 "These relations are still weakly supported, nevertheless, our method showed that they are consistent with the neighbourhood." What does this mean?<br><br>We apologize for this lack of clarity. Despite these relationships being supported by

Powered by Editorial Manager® and ProduXion Manager® from Aries Systems Corporation

only a few articles, the proposed approach showed that these are consistent with the literature of metabolic neighbours. These limited mentions could therefore be significant and deserve consideration. By using a standard ORA for comparison purposes, we also wanted to emphasize that the current volume of articles supporting a relation in the literature may simply not be sufficient (quantitatively) to effectively highlight it with this type of approach. We reworked the sentence as follows: “While only a few articles support these relationships and half of them were discarded by a standard ORA, the method showed their consistency with the literature of metabolic neighbours”.

2.6.3 Why do you want high entropy in Table 1? This isn't clear if the reader doesn't know what entropy refers to here.

As we removed the definition of Entropy in the method summary (see comment 2.3.9 and 2.3.10), we reworked this paragraph and added both an introduction oriented on the application of this indicator and a definition that we hope clearer. We replaced the paragraph “We also retained predictions based on well-balanced contributions from the neighbourhood by filtering on the diagnostic indicator Entropy > 1 (See details in Method and Supplementary S1.3).”, by: “Predictions for which the prior was biased toward one dominant contributor and thus failed to capture the neighbourhood literature, were excluded by filtering on the diagnostic indicator Entropy > 1. Entropy is the Shanon entropy computed on the contributors weights in the prior: the more contributors with balanced weights, the higher the entropy. (See details in Method and Supplementary S1.3).” We again direct the reader to the Supplementary materials.

2.7 Limitations

2.7.1 "Although we kept it in our analysis for sake of exhaustively…" It is not clear what this means.

We apologize for the lack of clarity. We wanted to emphasize that despite influential compounds like ethanol could provide out-of-context relations, we did not apply a filter to try to exclude them. We reworked this sentence: “To avoid arbitrary filtering, we left to the
user the choice to keep associations with such compounds after review”.

2.7.2 Methods Your equations are not numbered correctly. Your equation (1) should be equation (2). It looks like you have 14 equations total, but only one is numbered.

All the equations have been numbered in the new version of the manuscript.

2.7.3 Settings
2.7.3.1 How did you choose the cutoff in equation (1)?

As the probability to be reached by a random walk depends on the shortest distances within a network, we define a threshold that scales with the size of the network. We set the threshold to $1/(n - 1)$, which is the probability that a metabolite would be randomly chosen among all potentially reachable metabolites. It is important to note that this threshold is a default value and can be changed when calling the script to compute the associations (option -q: The tolerance threshold).

2.7.3.2 "These aspects are illustrated in Figure 1.B: B…" This entire paragraph should be moved up to the Method and Data Description section.

The paragraph has been reworked and an equivalent description is provided in section “Methods and Data description”. Nevertheless, we believe that even in this more technical description of the method, providing a link with the illustration in Figure 1.B may help to capture the behaviour of the method.

2.8 Mixing neighbouring literature to build a prior
2.8.1 Explain what the Beta distribution parameters mean in the context of this study in detail and why you chose a Beta distribution.

Being defined in the interval [0,1], the Beta distribution is a suitable model for modelling proportion, which is precisely what we want to estimate: the proportion (or probability) of articles mentioning a metabolite, that also mention a disease. Secondly, the beta distribution is the conjugate prior of the Binomial distribution, modelling the number of observed successes in a sequence of n trials, which is also the type of data that we have: among ni articles mentioning a metabolite, yi (successes) mention a disease. From this, the Beta-binomial model appears as a suitable framework for the purpose of this work.

When building the prior, the essential assumption is that a priori a metabolite and a disease are independent concepts in the literature. For all metabolites in the network, we start by modelling their prior probability of mentioning the disease with a Beta distribution parameterized under this hypothesis: the average probability equals the overall probability P of mentioning the disease in an article. The Beta distribution is therefore parameterized by mean μ and sample size ν, which determine the values of the two shape parameters α and β. μ being fixed to P, ν is actually the only hyperparameter setting the initial prior. Also, since μ is set to P, this default prior would not suggest a relation using LogOdds or Log2FC. Additionally, ν is related to the amount of evidences in the literature needed to change this prior belief. Thus, one should not directly interpret the values of α and β, as the real fixed parameters are μ and ν. More explanations have been added to the method section regarding the Beta distribution and the implication of the parameters.

2.9 Updating prior and selecting novel associations
2.9.1 "In turn, Log2FC is much more sensitive to outlier contributors than LogOdds". Please provide a citation for this.

This is a common problem with outliers when an estimator is based on a mean, we reworded this sentence to highlight this and provided a reference for this statement in the manuscript.

2.10 References
2.10.1Check the formatting for #27. It is overlapping the text in the right-hand column.

This has been fixed.

2.10.2 If you're going to discuss the Pareto Principle (28), try to find a review article that describes this principle rather than referencing a textbook.

This reference comes from a collection of commissioned introductory review articles and is highly cited; we believe it is appropriate in this context, and sufficient to grasp the concept needed to understand this section.

2.11 Supplementary Material
2.11.1S1.3 Diagnostic Values

Here, it is clear what you mean by Entropy and why you want the value to be high rather than low. This should go in the main text. However, it is concerning that the meaning of the entropy cutoff changes with respect to the number of contributors. Consider using a weighted metric that has the same meaning regardless of the number of contributors.

We apologize for any confusion caused by the provided numerical examples. The meaning of Entropy remains the same regardless of the number of contributors. By considering a weighted metric that is normalized by the number of contributors, the

reviewer may be referring to normalized entropy, also known as Efficiency, which is the observed Entropy divided by the maximal entropy (log2(N)).
However, imposing a fixed proportion of maximum entropy regardless of the number of contributors is not reasonable. For example, reaching 50% of the maximal entropy is easier when there are 5 contributors than when there are 50. Therefore, our choice aims to maintain a balanced distribution of contributors, becoming more flexible as the number of contributors increases. To address this, we set the threshold for Entropy at 1. This means that when there are only 2 contributors, the maximum entropy is required, and as the number of contributors increases, we progressively relax this constraint on a logarithmic scale. For example, with 5 contributors 50% of the maximum entropy is required, for 10 contributors 30%, for 30 contributors 20%, for 100 contributors 15%, etc. We reformulated the corresponding paragraph in the supplementary materials. Finally, the selected threshold is a recommendation for the specific analysis we conducted where we aimed for stringency and users are free to set their own threshold according to their needs.

2.11.2S2. Supplementary Tables
Why are your LogOdds values infinite? If this is an issue with taking the log of 0, then you should set a cutoff such that the values do not go to infinity.

The posterior error that an article mentioning the metabolite k, would mention the disease more frequently than expected is noted CDF and corresponds to P(pk < P). As CDF tends to 0 for strong relationships, logOdds will logically tend to infinity. This is a float approximation issue that is also commonly encountered when dealing with p-values. In the same way, it seems inefficient to distinguish highly significant relationships based on their logOdds, and we rely instead on the Log2FC as an effect size to rank these relationships. Defining an arbitrary and precise cutoff for LogOdds values also seems difficult and would depend on the user's appreciation of "highly significant" relations. Thus, we argue that replacing infinite values for LogOdds using an arbitrary and constant cutoff would not benefit the ranking that is already performed with Log2FC in these cases.

2.11.3S3. Supplementary Figures
2.11.3.1 Figure S3 is low-resolution and difficult to read. Please include a higher-resolution version of this figure.

The figure has been updated in high-resolution.

2.11.3.2 The figures don't seem to match up with their references in the main text.
Mismatches between figures and references in the main text have been checked and corrected if needed.

2.11.3.3 All supplementary figures should be here (including S1 and all figures after S3).

We acknowledge the reviewer's suggestion to consolidate all the supplementary figures into a single section for better organization. However, almost all the supplementary figures (exception to Figures S2, S3, S4, S5) illustrate complementary analyses to evaluate the performances and behaviour of the method. Then, we believe that it would be beneficial to keep the majority of the supplementary figures within the flow of the different analyses. We recognize that this may come at the cost of better segmentation, but we feel it is necessary to facilitate the reader's reading and comprehension.

2.11.3.4 It is not clear what Figure S4C refers to in the main text. (now Supplementary Figure 5.C)

We apologize for this lack of clarity. The supplementary figure 5.C refers to the profile of the contributors when only 2 articles out of 33 would have mentioned the disease,

which would have been sufficient to suggest this relationship with the proposed approach. We reformulate with the following sentences: “It is noteworthy that even fewer co-mentions would have already shifted the balance of contributors in favour of dopamine and highlighted this relationship. The figure S5.C shows the contributor profiles is the case where only 2 articles had mentioned the disease, which would have been sufficient to highlight the relationship.” We also added more details in the legend of the Figure.

2.11.4S4.1 Damping factor α and theoretical sample size v: benchmark. The validation dataset needs to be described before the results are presented. At this point, the reader has no idea what the validation set is.

Indeed, we thank the reviewer and moved this section on top of the supplementary materials.

2.11.5S4.3 Evaluation using simulated overlooked metabolites
2.11.5.1 These results should be highlighted in the main text.

The sentence highlighting these results in the main text have been reworked: “To evaluate the performances of predictions based on the posterior distribution and the behaviour of the method on challenging cases, a supplementary analysis was conducted using simulated overlooked metabolites in Supplementary S4.4”. While the purpose of these analyses is to provide a more comprehensive evaluation of the proposed approach, we consider them to be secondary. Consequently, we prefer to redirect the interested readers to these sections for further information without elaborating on these observations in the body of the article.

2.11.5.2 "Focusing on overlooked metabolites, the most challenging scenarios are those where positive examples apparently show no co-mentions, and conversely, when co-mentions (e.g. anecdotal) wrongly support negative examples." Please highlight how you determined positive examples, negative examples, and co-mentions here.

We thank the reviewer for pointing out this lack of clarity. The purpose of this analysis is to evaluate the performances of the approach on what we call “Hard cases”, which correspond to a subset of the simulated data in S4.4. Similarly to S4.1, positive examples are pairs of metabolites and disease-related MeSH extracted from the FORUM KG (q-value ≤ 1e − 6 with BH correction and no weakness), while negative examples are created by random combinations. For the purpose of simulating overlooked metabolites' data, the number of co-mentions (number of articles mentioning the both and supporting the relation) were randomly generated from a binomial distribution. We reworked the section S4.4 to clarify these potential ambiguities.

2.11.6S4.4 Impact of the carbon skeleton graph on the predictions This should also be discussed in the main text.

We reworked the related paragraph in the main text to better highlight these results. Like the analysis on neglected metabolites, we consider this one as secondary and do not wish to detail the results in the main text.

Reviewer #3:
The authors present a tool (FORUM Literature Propagation) which is designed to help users query disease information relevant to a given metabolite by also querying a

metabolic neighbors. This is accomplished by using a predefined network of metabolism (Human1) and querying PubChem for compound details and PubMed for articles containing disease and metabolite information. The authors have created a tool that is useful in finding potential associations of disease to metabolite, even when no articles have been published related to the specific metabolite in question This appears to be a useful tool for hypothesis generation, but should be used with caution as the results are inferred associations that may be skewed by regulatory mechanisms, the presence of highly studied metabolites, and highly 'promiscuous' metabolites which interact in a number of different pathways.

This review will focus primarily on the usability of the tool and the communication of that within the text. Summarily I find this to be a well written manuscript that does a good job of outlining the problem/need and appears to offer a solution. I do have some suggestions for clarifying the manuscript:

3.1 In the first paragraph of Method and Data Description the authors define a 'metabolic neighborhood' as "compound consists of the metabolites that can be reached through a sequence of biochemical reactions." Authors go on to reference the tools used to build and constrain the model. It would be additive to add some brief description to what was done prior, in addition to the more through explanation in supplemental information.

We thank the reviewer for helping us improve the clarity of the manuscript. We decided to add the following sentence in the corresponding section to better describe what is done with the atom-mapping procedure: “This results in a compound graph, built by linking two compounds when they share at least one carbon and have a substrate-product relationship in at least one reaction.”.

3.2 Continuing the above point this manuscript would be aided by a workflow diagram clearly illustrating the order of operations including key elements such as: user input, local database searching (Human1?), and PubMed/PubChem searching, result aggregation.

We thank the reviewer for this idea. Following this suggestion we added such a diagram. However, since the workflow encompasses various components, such as the extraction of FORUM associations and the conversion of the metabolic network into RDF, which are not the primary focus of our article, we have chosen to include the workflow diagram in the supplementary materials.

3.3 Figure 1 aids the reader to visualize FORUMs literature query process. However, it is a very dense figure that is difficult to extrapolate meaning from without carefully reading the Method and Data Description section. Ideally, this figure would be able to be understood by looking at the figure and its caption (current caption only details Blocks A and B). +Having blocks A-F and metabolites named A-F is also confusing, consider changing metabolites to numbers or Greek letters

We are thankful to the reviewer for pointing this out. We changed the figure annotation in order to make it more self-explaining. We decided to keep the capital letters for Figures’ sub captions and renames the metabolites in lowercase. Some other details were also added to the figure according to different reviewers’ comments.

3.4 What database is being used to define the metabolic network (pathways) and what identifiers are used to search those pathways for metabolic neighbors? Is this the pruned Human1 metabolic network and CIDs? More clarity here, would also be addressed by adding the workflow diagram suggested previously.

The metabolic network comes from a conversion of the Human1 SBML into RDF, and its content can be accessed from its own species identifiers or any referenced external identifier (CID, Chebi…) using the closeMatch property in SPARQL query. We added the workflow diagram to make this clearer, and, in addition to the data structure schema in the on-line documentation, we plan on adding pre-built example queries on

Powered by Editorial Manager® and ProduXion Manager® from Aries Systems Corporation

	the endpoint web page in the next release to make the search process more comprehensible.<br><br>3.5 Are the total number of metabolites available to use in this tool the 2704 mentioned in the Analysis section? Can this curated library be downloaded?<br><br>2704 is the total number of metabolites in the pruned version of the Human 1 v1.7 metabolic network. Among these metabolites, those with less than 100 annotated articles were considered as overlooked (2113 metabolites) and selected for analysis. Recall that this initial selection only serves as a prioritization and the method can be applied on the complete dataset. The library can be downloaded in a tabular file and in RDF format from the FTP server. Its content can be queried using the listed endpoint, from which the list of mentioned compounds can be retrieved in tabular format.<br><br>3.6 It appears to be a major limitation of this tool that over half of the 2704 metabolites do not have annotated PubChem CIDs, limiting the effectiveness of the tool in searching disease relevance.<br><br>Indeed, despite efficient cross-reference retrieval initiatives such as MetaNetX, many metabolites do not have annotated CID and thus can't be linked to any scientific literature. It is worth noting that the proposed method purposely alleviates such shortcoming by providing plausible associations for such compounds.<br>However, while some of those non-referenced compounds might not have any mentioning articles (or too few to confidently derive associations), some could have brought useful information and improved the associations regarding the former.<br>We could not find any means to know how non-referenced compounds are distributed among those two groups. However, we believe that most compounds without CID would have yielded few or no articles, since we see a correlation between the number of mentioning articles and the prevalence of curated entries of such compounds in many databases, estimated by the number of retrieved cross-reference annotations.<br><br>A Boxplot comparing the number of annotated articles among metabolic species in the network with more of fewer annotations than the median can be found in the attached document.<br><br>3.7 In the discussion section the authors simply state "many cannot be mapped to their corresponding PubChem identifier." Why? PubChem has over 100 million compounds, surely all the metabolites in the Human1 database have PubChem entries.<br><br>Some entries in metabolic models correspond to abstract entities rather than metabolites, such as "biomass" or "lipid pool", which can explain a lack of PubChem reference. It is possible that other entries...
**Additional Information:**	
**Question**	**Response**
Are you submitting this manuscript to a special series or article collection?	No
**Experimental design and statistics**<br><br>Full details of the experimental design and statistical methods used should be given in the Methods section, as detailed in our Minimum Standards Reporting Checklist. Information essential to interpreting the data presented should be made available	Yes

Powered by Editorial Manager® and ProduXion Manager® from Aries Systems Corporation

in the figure legends.<br><br>Have you included all the information requested in your manuscript?	
**Resources**<br><br>A description of all resources used, including antibodies, cell lines, animals and software tools, with enough information to allow them to be uniquely identified, should be included in the Methods section. Authors are strongly encouraged to cite Research Resource Identifiers (RRIDs) for antibodies, model organisms and tools, where possible.<br><br>Have you included the information requested as detailed in our Minimum Standards Reporting Checklist?	Yes
**Availability of data and materials**<br><br>All datasets and code on which the conclusions of the paper rely must be either included in your submission or deposited in publicly available repositories (where available and ethically appropriate), referencing such data using a unique identifier in the references and in the "Availability of Data and Materials" section of your manuscript.<br><br>Have you have met the above requirement as detailed in our Minimum Standards Reporting Checklist?	Yes

Manuscript

Click here to access/download;Manuscript;Manuscript_revised.pdf

OXFORD (GIGA)^n SCIENCE

GigaScience, 2023, 1–12

doi: xx.xxxx/xxxx
Manuscript in Preparation
Paper

PAPER

Suggesting disease associations for overlooked metabolites using literature from metabolic neighbours

M. Delmas[1], O. Filangi[2], C. Duperier[3], N. Paulhe[3], F. Vinson[1,4], P. Rodriguez-Mier[1], F. Giacomoni[3], F. Jourdan[1,4] and C. Frainay[1,*]

[1]Toxalim (Research Center in Food Toxicology), Université de Toulouse, INRAE, ENVT, INP-Purpan, UPS, 31300 Toulouse, France and [2]IGEPP, INRAE, Institut Agro, Université de Rennes, Domaine de la Motte, 35653 Le Rheu, France and [3]Université Clermont Auvergne, INRAE, UNH, Plateforme d'Exploration du Métabolisme, MetaboHUB Clermont, F-63000 Clermont-Ferrand, France and [4]MetaboHUB-Metatoul, National Infrastructure of Metabolomics and Fluxomics, Toulouse, France

[*]clement.frainay@inrae.fr

Abstract

In human health research, metabolic signatures extracted from metabolomics data are a strong-added value for stratifying patients and identifying biomarkers. Nevertheless, one of the main challenges is to interpret and relate these lists of discriminant metabolites to pathological mechanisms. This task requires experts to combine their knowledge with information extracted from databases and the scientific literature. ~~However, we show that a large fraction of metabolites are rarely or never mentioned in the literature. Consequently, these overlooked metabolites are often set aside and the interpretation of metabolic signatures is restricted to a subset of the significant metabolites~~However, we show that the vast majority of compounds (> 99%) in the PubChem database lack annotated literature. This dearth of available information can have a direct impact on the interpretation of metabolic signatures, which is often restricted to a subset of significant metabolites. To suggest potential pathological phenotypes related to ~~these understudied~~overlooked metabolites which lack of annotated literature, we extend the 'guilt by association' principle to literature information by using a Bayesian framework. The underlying assumption is that the literature associated with the metabolic neighbours of a compound can provide valuable insights, or an *a priori*, into its biomedical context. The metabolic neighbourhood of a compound can be defined from a metabolic network and correspond to metabolites to which it is connected through biochemical reactions. With ~~this~~the proposed approach, we suggest more than 35,000 associations between 1,047 overlooked metabolites and 3,288 diseases (or disease families). All these newly inferred associations are freely available on the FORUM ftp server (See information at `https://github.com/eMetaboHUB/Forum-LiteraturePropagation`.).

Key words: Literature Mining, Bayesian statistics, Metabolic Network

Background

Omics experiments have become widespread in biomedical research, and are frequently used to study pathologies at the genome, transcriptome, proteome and metabolome levels. The subsequent discriminant analysis leads to a set (a signature) of genes, proteins or metabolites, reflecting alterations of the phenotype at different levels of post-genomic processes. The interpretation of these signatures requires gathering knowledge about each of its elements from the scientific literature and dedicated databases (DisGeNET[1], Uniprot[2], HMDB[3], CTD[4], MarkerDB[5], FORUM[6]). However~~, despite its exponential growth[7]~~, the scientific literature suffers from an imbalanced knowledge distribution. This topic has received much attention for genes and proteins[8, 9, 10, 11, 12], showing a highly skewed distribution of the number of articles mentioning each entity. ~~Consequently, this strong imbalance has an impact on the quantity and quality of gene annotations in databases.~~ Indeed, what is known as *the*

Compiled on: June 9, 2023.
Draft manuscript prepared by the author.

Key Points

- Most metabolites have little or no information available in the literature.
- We propose an original method leveraging information contained in the literature from metabolic neighbours.
- We provide more than 35000 suggested relations between overlooked metabolites and disease-related concepts.

Matthew effect[13], which refers to the saying "the rich get richer", is particularly valid in scientific communications. For instance, as reported in [9]: "*more than 75% of protein research still focuses on the 10% of proteins that were known before the genome was mapped*" and as reported in [12] "*all genes that had been reported upon by 1991 (corresponding to 16% of all genes) account for 49% of the literature of the year 2015.*".

~~Metablomics emerged later than its omics siblings, transcriptomics and proteomics, and has, like them, benefited from technological advancements, such as NMR and mass spectrometry.~~ While we are getting closer to a complete reconstruction of the human genome[14], our knowledge of the metabolome, i.e. the set of metabolites present in a biological system[15] , is still limited~~by technical constraints. Among them, the main limitations are the identification of unknown metabolites and the sometimes inaccurate identification of known ones. For instance, only a small fraction (< 20%) of metabolic spectra can be correctly annotated in an untargeted metabolic analysis. This disparity~~ . This is also reflected in the distribution of the number of articles mentioning each compound present in the PubChem Database. While only a small fraction of them are mentioned in thousands of articles, the majority remains rarely or never mentioned [16]. This imbalance has consequences for the interpretation of the signatures, which can rely solely on a subset of its members that are sufficiently covered to provide insights. In Human health research, it is therefore critical to bring knowledge to these ~~understudied~~overlooked compounds, by suggesting diseases that could be linked to them.

A metabolite is suspected to be impacted or involved in a particular disease through metabolism when an imbalance in its abundance has been observed in comparison to control cases. Moreover, metabolites are linked to each other by biochemical reactions, and therefore their abundances are also interdependent. Among other factors, the abundance of a compound can depend on the concentration of its precursors and, in turn, can also influence the rate of production of other compounds. Following the well known 'guilt by association' ~~hypothesis~~principle, we assume that: if a metabolite has been linked to a particular disease due to an imbalance in its abundance, metabolites that are connected to it by biochemical reactions, i.e. its metabolic neighbourhood, can also be suspected of being linked to this disease. Metabolic networks[17], built originally for modelling purposes, describe those substrate-product relations between compounds and thus provide a suitable support to extend these suspicions to metabolic neighbours. For Human, the reconstruction of the metabolic network (Human1 v1.7 [18]) contains 13,082 reactions and 8,378 metabolites. In other omics fields, network-based strategies following "guilt-by-association" principle have been applied to build several recommendation systems proposing new genes or proteins that could be related to a given disease from a list of known genes/proteins [19, 20, 21]. We also developed a similar approach for metabolic signatures using random walks in metabolic networks [22].

If a compound is rarely or never mentioned, we hypothetize that the literature in its surrounding neighbourhood may provide *a priori* knowledge on its biomedical context. To combine both this *a priori* and the available literature of the compound (if any) in the suggestions, we propose a method based on the Bayesian framework. The method returns several predictors to evaluate whether ~~a significant proportion of the articles mentioning a metabolite would also mention a disease~~a metabolite could be related to a disease. In addition, several indicators can be used to highlight the most influential metabolic neighbours in the suggestions.
~~All the required data were extracted from the FORUM Knowledge Graph (KG)[6].~~ Metabolic neighbourhoods were defined from the Human1 metabolic network[18] and co-mention data between metabolites and diseases were extracted from the FORUM Knowledge Graph (KG)[6]. The detailed workflow is presented in Supplementary Figure S2 FORUM contains significant associations between PubChem chemical compounds and MeSH biomedical descriptors based on their co-mention frequency in PubMed articles. We evaluated our hypothesis by testing whether significant associations between metabolites and diseases could be retrieved solely on the basis of the literature of their neighbours. We illustrate the behaviour of the method in two scenarios: a metabolite without available literature for which the prior is the only source of information (Hydroxytyrosol) and a rarely mentioned metabolite (5α-androstane-3,17-dione with 82 articles). Using this approach on human metabolic network, we suggested more than 35,000 new relations between overlooked metabolites and diseases (and disease families). The code and the data needed to reproduce the results are available at https://github.com/eMetaboHUB/Forum-LiteraturePropagation.

Method and Data Description

The core of the method is the construction of a prior distribution on the probability that an article mentioning a metabolite would also mention a particular disease. This distribution is estimated from the literature of its metabolic neighbourhood. The metabolic neighbourhood of a compound consists of the metabolites that can be reached through a sequence of biochemical reactions. It is defined from the Human1 metabolic network[18], which was pruned from spurious connections using an atom-mapping procedure[22] (see Supplementary S1.1). ~~In the following description of the method and subsequent analyses, overlooked metabolites will be divided into two categories: those without literature (1) and those that are rarely mentioned (2).~~ In this study we define a set of overlooked compounds as compounds with less than 100 retrieved mentioning article, which correspond to orders of magnitude below 4,799, the mean number of retrieved articles per compound (when any), and is close to the median number of articles, 172. It is worth mentioning that such threshold serves solely as a prioritization criterion, since the method applicability is not restricted to a given range of mentioning corpus sizes (although its relevance is less obvious when a sufficient corpus is already available). In the following description of the method and subsequent analyses, a distinction is also made between metabolites without any retrieved article (1) and metabolites with fewer than 100 annotated articles (2).

The Figure 1 summarizes all the steps in the proposed method. Figure 1.A introduces the example of a relation between an overlooked metabolite ***a*** and a disease. The prior distribution on the

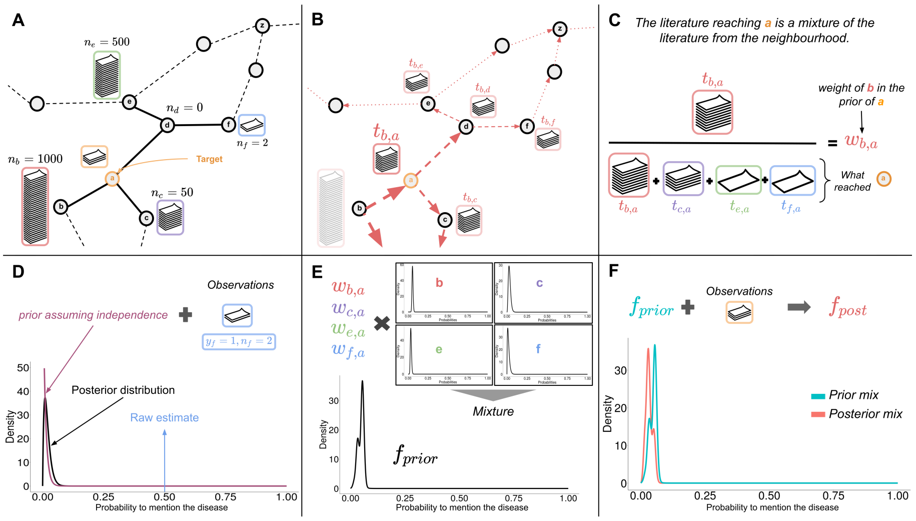

Figure 1. A step by step description of the proposed method. Compound a has $0 < n_a \leq 100$ articles, with some co-occurrence with the disease of interest ($0 \leq y_a \leq n_a$). In the blocks **A** and **B**, the nodes represent metabolites and the edges substrate/product relationships in the metabolic network. Dashed lines indicate more distant connections.A. Imbalance of mentioning literatures within a metabolic network. Compound a has $0 < n_a \leq 100$ articles, with some co-occurrence with the disease of interest ($0 \leq y_a \leq n_a$). Nodes represent metabolites and the edges substrate/product relationships in the metabolic network. Dashed lines indicate more distant connections. B. Propagation of literature through a metabolic neighbourhood. C. Weight of a metabolic neighbour in an overlooked metabolite's corpus used for prior construction. D. Contribution of a neighbour, from assumed independence, mitigated by neighbour's literature (observations). E. Construction of metabolite's prior from contributors. F. Computation of metabolite's posterior from observations and prior.

probability that an article mentioning a, would also mention the disease, is built from a mixture of the literature of its close neighbourhood in the metabolic network. The weight of the component of these metabolites in the mixture, depends both on their distance to a and their ~~amount of literature~~number of annotated articles (see details in section *Estimating the contributions of metabolic neighbours* in Methods). We also impose that a metabolite can't influence its own prior ~~or the prior of far distant metabolites~~. As an illustration, b shares a quantity $t_{b,a}$ of its literature to build the prior of a but doesn't influence its own prior~~, as well as the prior of Z~~ (Cf. Figure 1.B). The weight of b in the prior of a is then estimated as the ~~amount of literature~~number of articles it had shared with a, relative to the other neighbours c, e and f (See Figure 1.C). We refer to b, c, e and f as the *contributors* to the prior of a. Each contributor has a weight w in the prior of a (e.g $w_{b,a}$) proportional to its contribution. By analogy, it is as if each metabolite spreads its literature in the metabolic network, and the prior of a was built from the articles it had received from its contributors.

In Figure 1.D, the contributor f is also an overlooked metabolite with only 2 annotated articles, including one mentioning the disease. ~~This lack of literature may lead to a less reliable contribution. To avoid this issue, an initial shrinkage procedure is applied to all contributors. The probability distribution that one of its articles mentions the disease is readjusted toward the overall probability of mentioning the disease (see details in section *Mixing neighbouring literature to build a prior* in Methods).~~This results in a small sample size available to estimate the probability that an article mentioning f also mention the disease, which may lead to unreliable and spurious contributions. To address this, a shrinkage procedure is applied to all contributors, assuming that *a priori*, mentioning a metabolite in an article does not affect the probability of mentioning a particular disease. In Bayesian settings, a shrinkage estimator integrates information from the prior to readjusted raw estimates, reducing the effect of sampling variations (further details in section *Mixing neighbouring literature to build a prior* in Methods).

~~Then, we build the prior distribution for a, by mixing the probability distributions of each contributor (see Figure 1.E) according to their weights ($w_{.,a}$) estimated in the previous step (Figure 1.C).~~Then the prior distribution of a is built as a mixture of the probability distributions of individual contributors (b, c, e and f) as illustrated in Figure 1.E. Recall that the weight of each contributor in the mixture is $w_{.,a}$, as estimated in the previous step (see Figure 1.C). The prior mixture distribution is denoted by f_{prior}. The constructed prior distribution for a represents the probability distribution that an article from one of its contributors would mention the disease. In the scenario where a has no literature (1), the predictions will be based solely on f_{prior}.

However if a is mentioned in few articles (2), we compute the posterior distribution, thus updating the weights and distributions of each contributor in the mixture (Figure 1.E). The posterior mixture distribution is denoted by f_{post}.

From the mixture distribution, two predictors are estimated: *LogOdds* and *Log*$_2$*FC*. *LogOdds* expresses the ratio between the probability of the disease being mentioned more frequently than expected in the literature of the compound, rather than less frequently. *Log*$_2$*FC* expresses the change between the average probability of mentioning the disease in the mixture distribution, compared to the expected probability in the whole literature. In summary, both should be considered jointly in the predictions: *LogOdds* as a measure of significance and *Log*$_2$*FC* as a measure of effect size. In (2), to get an intuition about the belief of the neighbourhood only, we also return similar indicators estimated from f_{prior}: *priorLogOdds* and *priorLog*$_2$*FC* (see sections *Updating prior and selecting novel associations* and *Different scenarios* in Methods). ~~Finally, several diagnostic values such as *Entropy* allow assessing the composition of the built prior (See Supplementary S1.3). *Entropy* evaluates the good balance of contributions in the prior. The more metabolites contribute to the mixture and the more their weights are uniformly distributed, the higher the entropy.~~Finally, given its primary role in driving predictions, assessing the composition of the constructed prior is crucial. Essentially, the more contributors to the prior, close to the target compound, with balanced weights, the better it captures the neighbourhood literature and increases the confidence in predictions. To aid in this evaluation, a set of diagnostic indicators is presented in Supplementary S1.3.

Analyses

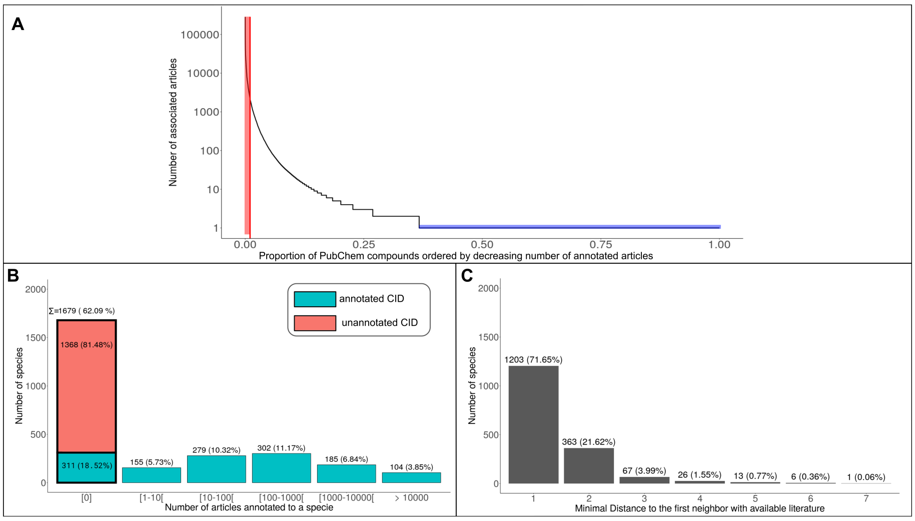

Figure 2. A: Distribution of the number of annotated articles (expressed in log-scale) for PubChem compounds that have at least one article in FORUM, in descending order. The red area represents the proportion of the most mentioned compounds required to attain 80% of the total number of annotations, while the blue area represents the fraction of compounds with only one annotated article. **B**: Distribution of the number of annotated articles per metabolites, organised by bins, in the carbon skeleton graph of Human1. The first bar represents the metabolites without literature. Among them, 81.5% don't have annotated PubChem identifiers, making it impossible to link them to PubMed articles with FORUM. The remaining 18.5% have annotated PubChem identifiers, but no articles were found mentioning them. In total, there are 1336 compounds with an available PubChem identifier. **C**: Distribution of the shortest distance to the first neighbour in the metabolic network with at least one annotated article, for the metabolites without literature in the network (bold bar of **B**). The distances were computed with the Dijkstra algorithm.

Unbalanced distribution of the literature related to chemical compounds

The FORUM KG links PubChem compounds to the PubMed articles that mention them. Among the 103 million PubChem compounds in FORUM, only 376,508 are mentioned in PubMed articles, representing a coverage lower than 0.4%. For these mentioned compounds, the distribution of the literature is highly skewed (Figure 2.A). The top 1% of the most mentioned compounds (red area) concentrates 80% of the links between PubChem compounds and PubMed articles. Similarly, the blue area indicates that 63% of compounds (218,291) have only one article mentioning them, which, to give a point of comparison, is cumulatively less than the literature associated with glucose: 278,277 distinct articles.

Considering only metabolites, Figure 2.B presents the distribution of the number of articles mentioning the 2704 metabolites, conserved in the pruned version of the Human1 metabolic network. Because of the skewed distribution of the literature and the lack of external identifiers, 62.09% of the metabolites in the metabolic network have no annotated articles. Nevertheless, almost 72% of them have at least one direct neighbour in the metabolic network with available literature (See Figure 2.C). Moreover, by considering the close neighbourhood (paths up to three reactions), almost all the metabolites ($\approx$ 97.26%) without initial literature can reach a described neighbour, showing the availability of nearby literature to build a prior.

Evaluation of the prior computation

The critical step in the proposed method is the construction of a relevant prior. While its influence on the results will decrease as the size of the literature of the targeted compound increases, it will mainly drive the predictions for the rarely mentioned compounds we are interested in [23].

The relevance of the prior was evaluated by testing whether significant associations with diseases, could be retrieved using only the literature from the metabolic neighbourhood of the metabolite. The validation dataset includes 10,000 significant relations between metabolites and disease-related MeSH extracted from the FORUM KG, and 10,000 random metabolite-MeSH pairs to serve as negative examples. The method is evaluated by considering either the direct or a larger neighbourhood (metabolites that can be reached through a path of two or more reactions). ~~In the method, the considered neighbourhood is controlled by the parameter α (see details in section *Estimating the contributions of metabolic neighbours* in Methods and Supplementary S4.1) and is set to α = 0 for the direct neighbourhood and α = 0.4 for a larger one.~~ We therefore focused on two specific settings: α = 0, where solely the direct neighbours contribute to the prior, and α = 0.4, where contributions between direct or indirect neighbours are relatively balanced. The impact of the parameter α on the construction of the prior and the Precision-Recall tradeoff was extensively evaluated in Supplementary Material S4.3.

We decided to compare the proposed method against two different baselines (more details in Supplementary S4.2). Baseline-Freq is the most naive approach in which the predictions are solely based on the overall probability of mentioning the disease, such that a metabolite is more likely to be related to frequently mentioned diseases in the literature. Hence, Baseline-Freq ignores the network information (metabolic neighbourhood). On the contrary, the predictions with Baseline-DN are based on the average probability of mentioning the disease in the direct neighbourhood, thus closer to the proposed approach. It is worth noting that, if all direct neighbours have relatively the same ~~amount~~number of annotated articles and are well covered (negligible shrinkage), the method parameterized with α = 0 behaves like the simple Baseline-DN for metabolites without literature. We used Log_2FC as predictor for the proposed method ~~in Figure 3~~.

The evaluation results on the validation dataset for all described approaches are presented in Figure 3. All tested approaches outperform Baseline-Freq, showing the benefit of examining the neighbouring literature. When considering the direct neighbourhood (method with α = 0), the method is more efficient than Baseline-DN. However, as previously shown in Figure 2.C, the direct neighbourhood cannot bring information for more than 28% of metabolites without literature. Therefore, considering a larger neighbourhood can be essential for some overlooked metabolites, and the approach achieves solid performances (AUC=0.78) on the validation dataset with α = 0.4. Applying a threshold on $Log_2FC > 1$ results in a TPR=0.35 and a FPR=0.05. Using *LogOdds* as predictor, the method achieved slightly lower performances (AUC=0.76),

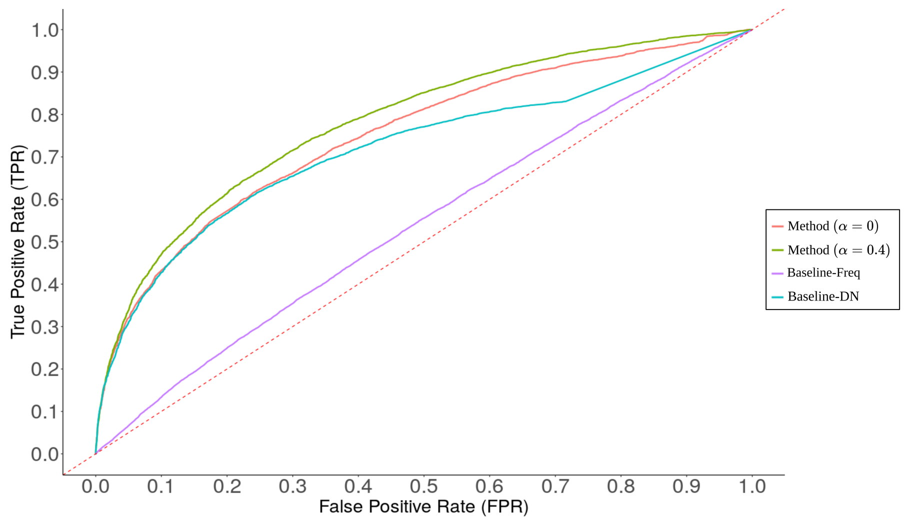

Figure 3. Receiver operating characteristic (ROC) of the method considering only the direct neighbourhood ($\alpha = 0$) or a larger ($\alpha = 0.4$) and two different baselines. For Baseline-Freq the predictions are only based on the overall probability of mentioning the disease in the literature. For Baseline-DN the predictions are based on the ratio between the average probability of mentioning the disease in the direct neighbourhood and its overall probability. Respective AUC (Area Under the Curve) for Method ($\alpha = 0$), Method ($\alpha = 0.4$), Baseline-DN and Baseline-Freq are: 0.75, 0.78, 0.72 and 0.54. A true positive represents an association between a compound and a MeSH term which is both retrieved from the compound's mentioning corpus using Fisher Exact Test, and from methods in which no knowledge of such corpus is available. A false positive is only retrieved from the latter.

with a TPR=0.22 and a FPR=0.04 when applying a threshold on *LogOdds* > 2. Beyond the validation, *LogOdds* is more robust to outlier contributions than Log_2FC and when examining predictions, they should be considered together as complementary indicators of significance and effect size. These results suggest that the prior built from the neighbouring literature alone, holds relevant information about the biomedical context of metabolites and could be efficient to drive predictions for rarely mentioned compounds. ~~To evaluate the performance of predictions based on the posterior distribution, a complementary analysis is provided in Supplementary S4.3.~~ To evaluate the performances of predictions based on the posterior distribution and the behaviour of the method on challenging cases, a supplementary analysis was conducted using simulated overlooked metabolites in Supplementary S4.4. Finally, as mentioned in the Method summary, the metabolic network was pruned from spurious connections using an atom-mapping procedure (see Supplementary S1.1). This results in a compound graph, built by linking two compounds when they share at least one carbon and have a substrat-product relationship in at least one reaction. The ~~benefit of this procedure on the predictions~~impact of the carbon skeleton graph on the predictions is evaluated in Supplementary S4.5.

Suggesting relations with diseases for overlooked metabolites

In the FORUM KG, 80% of the significant associations with biomedical concepts are observed for the 20% of compounds with more than 100 annotated articles. This manifestation of the Pareto principle[24] reflects the need for additional knowledge for compounds that are less frequently mentioned. Therefore in this analysis, we applied the proposed method on all metabolites in the human metabolic network with less than 100 annotated articles (see Table 1). According to the experiments on the validation dataset (See previous section *Evaluation of the prior computation*), we applied a threshold on *LogOdds* > 2 and Log_2FC > 1. ~~We also retained predictions based on well-balanced contributions from the neighbourhood by filtering on the diagnostic indicator *Entropy* > 1 (See details in Method and Supplementary S1.3).~~Predictions for which the prior was biased toward one dominant contributor and thus failed to capture the neighbourhood literature, were excluded by filtering on the diagnostic indicator *Entropy* > 1 *Entropy* is the Shannon entropy computed on the contributors weights in the prior: the more contributors with balanced weights, the higher the entropy. (See details in Method and Supplementary S1.3).

1863 predictions correspond to relations that are not novel, since they are already supported by one or several publications in the literature (`co-mention:yes` in Table 1). ~~However, by re-evaluating these predictions using a right-tailed Fisher exact Test (BH correction and selecting those with *q.value* <= 0.05), we found that ≈ 50% of them (925) would not have been found significant.~~ However, by re-evaluating them using the same workflow as in FORUM[6] (a standard over-representation analysis (ORA) using right-tailed Fisher exact Test, BH correction and threshold on q.value $\leq$ 0.05), we found that ≈ 50% of these associations (925) would not have been highlighted. ~~These relations are still weakly supported, nevertheless, our method showed that they are consistent with the neighbourhood~~ While only a few articles support these relationships and half of them were discarded by a standard ORA, the method showed their consistency with the literature of metabolic neighbours. 7,286 novel relations have also been suggested with disease-related MeSH, without having already been mentioned in their literature (`co-mention:no`). Finally, for 793 metabolites without literature, 26,436 relations have been suggested only by exploiting the neighbourhood literature. All the results are available on the FORUM ftp server (See `https://github.com/eMetaboHUB/Forum-LiteraturePropagation`.), filling a gap when it comes to the interpretation of signatures with these ~~understudied~~overlooked metabolites.

Case study

In this section, we will describe the behaviour and benefits of the method through two test cases. As mentioned in the previous section *Method and Data Description*, Hydroxytyrosol is an example of a metabolite without literature (1) and 5alpha-androstane-3,17-dione of a metabolite with only a few annotated articles (2) and with a weakly supported association.

	Nb. metabolites	co-mention	Nb. predictions
**Metabolites without literature**	793	no	26,436
**Metabolites with few articles (< 100 articles)**	254	no<br>yes	7,286<br>1863

Table 1. Summary table of the number of disease-related MeSH predicted for metabolites in the network with less than 100 annotated articles. The results are separated between the two major scenarios: (1) Metabolites without literature and (2) metabolites poorly described in the literature (< 100 articles). In the second case, results are also arranged according to whether the metabolite already co-mentions the MeSH (co-mention column). Only predictions with *LogOdds* > 2, Log_2FC > 1 and *Entropy* > 1 are considered. For the 1863 predictions where the metabolite co-mentions the MeSH, 938 (≈ 50%) are also retrieved using a right-tailed Fisher exact test (BH correction and *q.value* < 0.05). Only 793 metabolites among the 1679 without literature and 254 among those with literature have significant results according to the used thresholds.

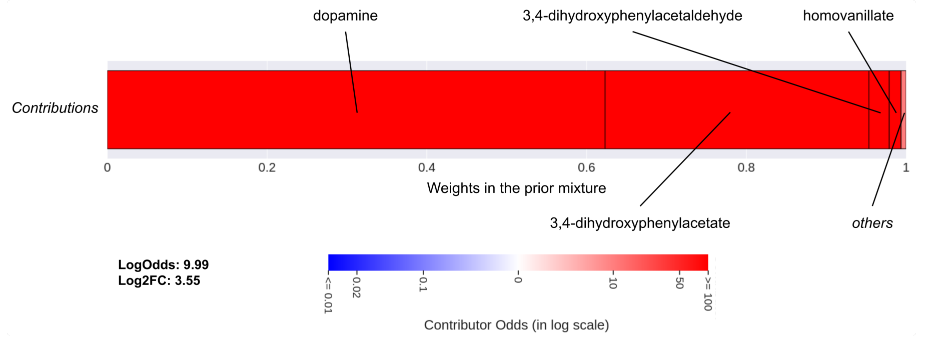

Figure 4. Profile of the contributors for the association between Hydroxytyrosol and Parkinson's Disease. This shows the repartition of the literature received by Hydroxytyrosol from its neighbourhood to build its *prior*. Contributors are organised in blocks by increasing weights in the prior mixture ($w_{i,k}$), from left to right. The weights also give the width of the block. The colour of each block associated with a contributor depends on its individual *LogOdds*, from blue to red, for *negative* (less likely) to *positive* (more likely) contributions respectively. Weights and *LogOdds* are also detailed in Supplementary Table S2.

Hydroxytyrosol and its potential link with Parkinson's disease

Hydroxytyrosol is a metabolite which is known for its antioxidant properties [25] and mentioned by 856 publications in FORUM. However, its literature will only serve as ground truth, and Hydroxytyrosol will be considered as a metabolite without literature in this analysis. Consequently, the predictions are solely derived from the neighbouring literature (f_{prior}). The top 10 predictions ranked by *LogOdds* are presented in Supplementary Table S1. Parkinson's disease is the most suggested disease, followed by broader descriptors also related to neurodegenerative disorders. This suggestion is mainly driven by the literature of close metabolic neighbours: dopamine and 3,4-dihydroxyphenylacetate (Figure 4). Both compounds' literature frequently mention Parkinson's Disease (Supplementary Table S2) suggesting that Hydroxytyrosol may also be related to this disease. Other contributors such as 3,4-dihydroxyphenylacetaldehyde or homovanillate also seem to be related to the pathology but only contribute ≈ 5% to the prior as they are more distant neighbours or have less literature. In the actual literature of Hydroxytyrosol, 2 articles[26, 27] explicitly discuss its therapeutic properties on Parkinson's disease.

Highlighting the role of 5alpha-androstane-3,17-dione in Polycystic Ovary Syndrome

Since 82 articles are available for 5-α-androstane-3,17-dione (5-αA), the predictions are derived from both its literature and that of its metabolic neighbourhood. The top 25 predictions ranked by *LogOdds* are presented in Supplementary Table S3, along with the p-value from a right-tailed Fisher exact test using the same data for comparison. The highest ranked associations are both supported by several mentions of the compound and by the neighbourhood (high *priorLogOdds*). They correspond to mildly-interesting predictions as the literature of the compound alone would have been sufficient (significant Fisher p-value): the neighbourhood only strengthens the relation. Instead, we choose to focus on the relation with Polycystic Ovary Syndrome (PCOS) which has a non-significant Fisher p-value and only one article supporting the relation [28]. The *priorLogOdds* (5.47) indicates that the literature gathered from the metabolic neighbourhood seems highly related to the disease (Figure 5). While the literature of the compound alone is insufficient to highlight an association with PCOS, the posterior distribution, combining information available from the compound and its neighbours, strongly suggests one (*LogOdds* = 6.23 and Log_2FC = 3.14). Androsterone, a direct neighbour of 5-αA through the reaction *3(or 17)-alpha-hydroxysteroid dehydrogenase*, is the main contributor supporting the prediction (Figure 5). Additional contributors such as testosterone, testosterone-sulfate, estradiol-17β and progesterone are more distant metabolically (2-3 reactions) but are also frequently mentioned in this context [29, 30, 31, 32, 33, 34, 35]. Also, PCOS is much more frequently mentioned in the literature of 4-androstene-3,17-dione compared to the other metabolites in the neighbourhood, making it an outlier among the contributors. Interestingly, its contribution significantly drops in the posterior distribution (See details in Supplementary materials ~~S4.5~~S4.6 and Supplementary Table S4). A view of the metabolic neighbourhood of 5-αA is also presented in Supplementary Figure S4.
To illustrate the influence of the observations on the posterior distribution, we re-evaluated the relation by removing the single co-occurrence between the 5-αA and PCOS. By suppressing this mention, the *LogOdds* drops to 3.67, Log_2FC to 2.80, and the weights in the posterior mixture change according to the new observations (See Supplementary Figure S~~4~~3). For instance the weight of androsterone, which literature mentions PCOS less frequently than the other top contributors (testosterone, estradiol, etc.), increased while those of the others decreased. More significantly, the weight of 16alpha-hydroxydehydroepiandrosterone, which is never mentioned with the disease, increases from 0.38% to 3%. By removing this mention, the likelihood of the evidences for each contributor changed, favouring those for whom the disease is less likely to be mentioned in an article. Although the relation is still suggested by the neighbourhood, this result shows the impact of the available literature on the predictions.

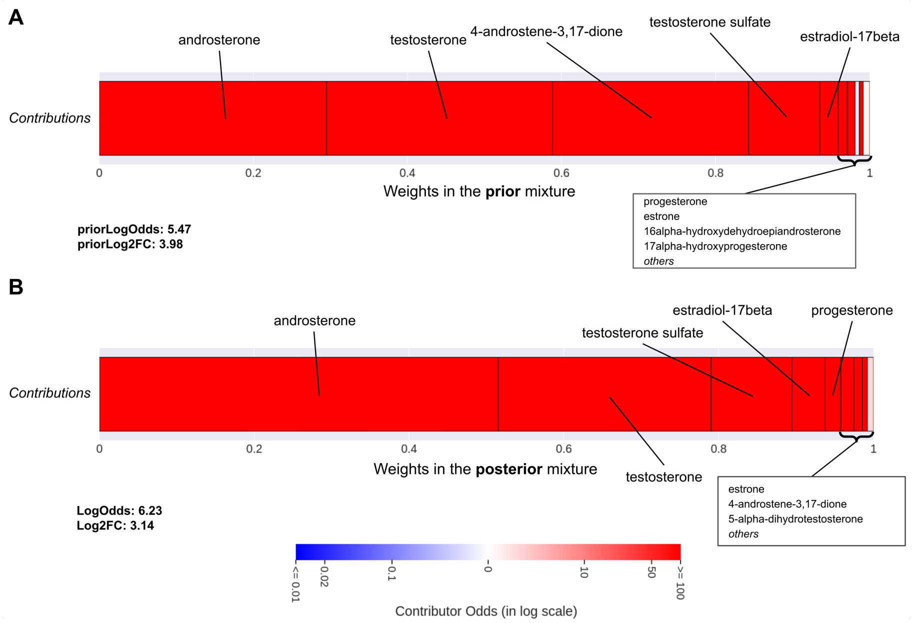

Figure 5. Profile of the contributors for the association between 5alpha-androstane-3,17-dione and Polycystic Ovary Syndrome in the **prior** mixture (**A**) and in the **posterior** mixture (**B**). Contributors are organised in blocks by increasing weights in the mixture from left to right, and the weights also give the width of the block. The colour of each block associated with a contributor depends on its individual *LogOdds*, from blue to red, for *negative* (less likely) to *positive* (more likely) contributions respectively. Details in Supplementary Table S4.

Discussion

The interpretation of experimental results in metabolomics requires an intensive dive in the scientific literature. In a biomedical context, researchers often seek studies that mention metabolites from an observed signature, as well as report variations in their concentration in similar phenotypes. However, we have shown that there is a strong imbalance in the distribution of the literature among metabolites, suggesting that this research could be restricted to a subset of the initial metabolic signature. Even if this imbalance is accentuated by technical limitations, it also reflects biological facts: some metabolites are more central and sensitive to phenotypic alterations and would therefore be more frequently reported. Nonetheless, they do not necessarily provide key information when interpreting results, because they do not point to dysregulations on specific pathways. To extend the available data to help interpret results, we propose a method to suggest relations between ~~understudied~~overlooked metabolites and diseases. Most metabolites (62%) in the network have no literature available, and many cannot be mapped to their corresponding PubChem identifier. It is a common issue when dealing with metabolic networks, as they are initially built for modelling purposes [36]. The absence of annotations also indicates that a compound is not widely described and studied, which may suggest that little literature has actually been lost.

The predictions for metabolites without literature are solely based on their prior distribution which is built from a mixture of the neighbouring literature. We first evaluated the prior alone on a validation dataset (AUC $\approx$ 0.78) and showed that it holds relevant information about the biomedical context of metabolites. Since the contributors, their weights, and influences in the mixture distribution (more or less likely to mention the disease in an article) are known, the prior is transparent by design. In the example of hydroxytyrosol, the prediction was mainly derived from the literature of dopamine, 3-4-dihydroxyphenylacetaldehyde (DOPAL), 3,4-dihydroxyphenylacetate (DOPAC) who all frequently mention Parkinson's disease in their literature. Hydroxytyrosol and its contributors belong to the dopamine degradation pathway [37]. The literature supporting the relation with Parkinson's disease mainly discusses the production of hydrogen peroxide during dopamine degradation to DOPAL by MAO enzymes. Since DOPAL is then inactivated into either DOPAC or Hydroxytyrosol, the literature that has been propagated by the contributors is metabolically relevant for hydroxytyrosol. Indeed, [38] shows that Hydroxytyrosol can induce a negative feedback inhibition on dopamine synthesis resulting in a decrease of the oxidation rate of dopamine. By indicating which and how neighbours contributed to the predictions, the contribution profile thus adds explainability to the predictions, which we believe is an important quality of the method. It can be quickly established if there was a clear consensus in the neighbourhood or if the association was only carried by one dominant contributor. In the case of *positive* suggestions, the associated literature of each contributor could be examined to understand the nature of their relation with the disease and assess the consistency of the prediction. Typically, we want to evaluate whether the relationship between the contributors and the disease can indeed be transferred to the target compound, whether it may suggest another, or whether it is irrelevant.

While a consensus is of course preferred (not matter the outcome of the prediction), some contributors may also have divergent literature for a particular disease. To complete the example of hydroxytyrosol, we show the profile of the contributors for the relation between 5-S-Cysteinyldopamine (CysDA) and Parkinson's disease (See Supplementary Figure S65.A). CysDA is the S-conjugate of dopamine and cysteine and its prior is mainly influenced by the literature of both of these precursors, at 51% and 45% respectively. While dopamine is strongly related to the disease, cysteine is much less mentioned in this context and the prior is consequently indecisive (*priorLogOdds* $\approx$ 0.1). In this case, only the observed literature of CysDA can reduce the uncertainty by updating the prior distribution. In FORUM, 11 articles out of 33 mention CysDA and Parkinson's disease, which has an important impact on the weights in the posterior mixture in favour of dopamine, which then becomes the dominant contributor (See Supplementary Figure S65.B). Indeed, the posterior weights are proportional to the likelihood of the data according to the prior defined by each contributor. For CysDA, observations clearly suggest that it should be frequently mentioned with Parkinson's disease, like dopamine, contrary to what is suggested by cysteine. The prediction is highly signifi-

cant (*LogOdds* = 50.7, Log_2FC = 3.87) as the literature of CysDA is very indicative. However, we would have already suggested the relation if only 2 articles out of 33 had mentioned the disease (see Supplementary Figure S6.C).It is noteworthy that even fewer co-mentions would have already shifted the balance of contributors in favour of dopamine and highlighted this relationship. The figure S5.C shows the contributor profiles is the case where only 2 articles had mentioned the disease, which would have been sufficient to highlight the relationship. This emphasize the sensibility of the method which may suggest still poorly supported relations, but which are consistent with the metabolic neighbourhood's literature.

Likewise, the literature linking 5-αA to PCOS is not sufficient in quantity to statistically show a relation. From an expert's perspective, only one qualitative article could be sufficient to justify a relation between a metabolite and a disease. But since the literature and the topics related with metabolomics are broad, highlighting these weakly supported relations could point to relevant paths of interpretation that may have been missed. The relation between 5-αA and PCOS is supported by only one article but is highly coherent in the metabolic neighbourhood, as androgen metabolism dysfunctions are central in this pathology [39]. As the contributors are widely studied metabolites (androsterone, testosterone, ...) that also frequently mention the disease in their literature, the prior regarding the relationship is strong and strengthens the observations. We also show that after removing the only supporting article and computing the posterior distribution accordingly, the relation is still suggested but the *LogOdds* and Log_2FC significantly drops. This illustrates the behaviour of the method, where the posterior distribution proposes a compromise between the compound's literature and that of its contributors, giving more weight to those that are the most mentioned and for whom the observations are the most consistent. The neighbourhood literature can also help to discard suggestions that are supported by secondary or negligible mentions (See Supplementary S4.6S4.7).

With FORUM's data, relations are evaluated for both disease-specific MeSH and broader descriptors, representative of disease families such as *Neurodegenerative Diseases* (D019636). When there is no consensus among contributors at the level of specific diseases but they all belong to the same category of disorders, it could allow to suggest more coarse-grained relations. Although this increases the redundancy of the results, it makes it easier to grasp the overall biomedical context of some understudiedoverlooked metabolites.

Limitations

The most evident limitation of the proposed approach is that the assumption that the literature in the metabolic neighbourhood of a metabolite provides relevant prior knowledge on its biomedical context, is not always accurate. A short path of reactions can indeed have a major impact on the metabolic activity of compounds, resulting in separate biological pathways and invalidating the hypothesis. For instance, while dopamine is a derivative of tyrosine, the former is a neurotransmitter and the latter a fundamental amino acid. Their biomedical literature therefore covers very different topics, and one would not provide a good *a priori* on the other. Nonetheless, thanks to the transparency of the contributors' profile, such irrelevant contributions can be identified and the corresponding predictions re-evaluated or discarded.

Based solely on the metabolic network, we ignore the regulatory mechanisms of biological pathways and only focus on biochemistry. We therefore assume that all paths of reactions are active and valid when propagating the literature, which is not true and may vary depending on physiological conditions. The predictions could potentially be improved by integrating a regulation layer, but this would add major complexity to the method and we choose to ignore these constraints by proposing a more general approach. Although reconstructions of the human metabolism like Human1 are constantly improving, they remain incomplete and some pathways (eg. lipids[40]) are simplified with missing or artificially created links, mainly for modelling purposes.

With their overflowing literature, overstudied metabolites (amino acids, cholesterol, etc.) can erase the contributions of other neighbours in the construction of a prior. This results in a strong prior which is only fuelled by the literature of one dominant contributor, and in the case of a metabolite without literature, predictions will therefore be solely based on it. We therefore provide diagnostic indicators like *Entropy*, *CtbAvgDistance* and *CtbAvgCorporaSize* (See Supplementary S1.3) to identify these unbalanced priors and flag these predictions. Finally, a part of the biomedical literature of some influential compounds may not be related to their metabolic activity. For instance, ethanol is strongly related to bacterial infections, not as a metabolite but because of its antiseptic properties, which may suggest out-of-context relations by spreading its literature to neighbours. Although we kept it in our analysis for sake of exhaustively Then, it could be beneficial to remove its literature and that of metabolites with similar behaviours, for predictions on their close neighbours.To avoid arbitrary filtering, we left to the user the choice to keep associations with such compounds after review.

Potential implications

Based on the literature extracted from the FORUM KG, we showed the imbalance in the distribution of the literature related to metabolites. To overcome this bias, we proposed an approach in which we extend the *guilt by association* principle in the Bayesian framework. Basically, we use a mixture of the literature of the metabolic neighbourhood of a compound to build a prior distribution on the probability that one of its articles would mention a particular disease. The transparency of the contributor's profile is essential and helps diagnose and explain the predictions by indicating which and how metabolic neighbours have contributed. More than 35,000 relations between metabolites and disease-related MeSH descriptors have been extracted and are available on the FORUM ftp. These relations may help interpret metabolic signatures when no or little information can be found in the literature or databases. In the upcoming release of the FORUM KG, these relations will be integrated as a peripheral graph to supplement the existing metabolite-disease associations and create new paths of hypotheses. In this analysis we restricted our predictions to diseases-related concept because the metabolic network, although suitable for propagating this type of relationship, would be less reliable for propagating functional relations for instance. The process is also network dependent, which means that using a different metabolic network (human or other organisms) could result in different suggestions. Nonetheless, the approach could be extended to other entities (genes, proteins) and relations, as long as the related literature is available and the neighbourhood of an individual can provide a meaningful prior. Finally, as the literature grows rapidly and metabolic networks become more comprehensive, we hope that this will also improve both the quantity and quality of the suggestions in the future.

Methods

Settings

The approach is metabolite-centric, considering all the available literature for each metabolite and its co-mentions with disease-related MeSH descriptors as input data. Note that each article frequently mentions numerous metabolites and therefore the literature related to each metabolite, in terms of publications, is not exclusive to that chemical, but can be shared with others. We thus

call a 'mention' the fact that an article mentions a metabolite.
For M metabolites in the metabolic network, we note n_i the total number of mentions of a metabolite i and then define $N = \sum_{i=1}^{M} n_i$ as the total number of mentions in the network. Given a specific disease-related MeSH descriptor, we also define y_i as the number of articles co-mentioning the metabolite i and the disease, with $m = \sum_{i=1}^{M} y_i$ the total number of mentions involving that disease. Details on the extraction of literature data from the FORUM KG are presented in Supplementary S1.2.
For a metabolite k of interest, the random variable p_k denotes the probability that an article mentioning the metabolite k, also mentions the disease. The aim of the method is to estimate the posterior distribution of p_k, given a prior built from the literature of its metabolic neighbourhood. To assess the strength of their relation, p_k is then compared to the expected probability $P = \frac{m}{N}$ that any mentions of a metabolite in the literature also involves the disease. As in the method summary, the scenario in Figure 1 will be used to illustrate the different steps.

Estimating the contributions of metabolic neighbours

Based on the assumption that the literature from the metabolic neighbourhood of a compound could provide a useful prior on its biomedical context, the first step is to propagate the neighbours' literature. A random walk with restart (RWR) algorithm (or Personalized PageRank) is used to model a mention, sent by a metabolite i, which moves randomly through the edges in the network and reaches another compound k. At each step, the mention has a probability α, named the *damping factor*, of continuing the walk and $(1 - \alpha)$ of restarting from the metabolite i. The result is a probability vector $\pi_{i,.}$, indicating the probability that a mention sent by i reaches any metabolites k in the network, noted $\pi_{i,k}$. The expected number of mentions sent by i that reach the compound k are then $\pi_{i,k} n_i$. However, in this model, a compound can receive its own mentions ($\pi_{k,k} > 0$) although only those derived from the neighbourhood should be used to build the prior, as the metabolite should not influence itself. A second bias is relative to the set of neighbours for which a metabolite is *allowed* to contribute to their prior. Metabolites with very large corpora (Glucose, Tryptophan, etc.) can propagate their literature to distant metabolites in the network, even if their probability to reach them is low. In the case of metabolites with a rarely mentioned direct neighbourhood, they can predominantly contribute to the prior, although they are not metabolically relevant. This bias is accentuated by the highly skewed distribution of the literature.
To contribute to the prior of k, we therefore require that a metabolite i should have a probability of reaching k (without considering the walks that land on itself) greater than the probability of choosing k randomly. The set of metabolites k to which i is allowed to contribute, namely the influence neighbourhood of i, noted H_i, is therefore defined as :

$$k \in H_i \quad \forall k \neq i, \frac{\pi_{i,k}}{(1 - \pi_{i,i})} > \frac{1}{(n - 1)} \tag{1}$$

According to these probabilities, the quantity of literature sent by i that reaches k is noted $t_{i,k}$ such as:

$$t_{i,k} = \begin{cases} \frac{\pi_{i,k}}{\sum_{k' \in H_i} \pi_{i,k'}} n_i \text{ if } k \in H_i. \\ 0, \text{otherwise.} \end{cases} \tag{2}$$

These aspects are illustrated in Figure 1.B: ~~B propagates its literature to its neighbourhood but no mentions return to B, B is not allowed to send mentions to Z (being too far) and A receives $t_{B,A}$ mentions from B~~ **b** does not share any mentions with itself, nor with **z**, which does no bot belong to its influence neighbourhood in this example. However, **a** receives $t_{b,a}$ mentions from **b**. Symmetrically, we defined T_k as the set of contributors of k, such that $t_{i,k} > 0$. Each contributor i, has a weight $w_{i,k}$ in the prior of k, representing the proportion of literature reaching k, that was sent by i:

$$w_{i,k} = \frac{t_{i,k}}{\sum_{i' \in T_k} t_{i',k}} \tag{3}$$

The weight vector for compound k is noted $\mathbf{w_k}$. In Figure 1.C, $w_{\mathbf{b},\mathbf{a}}$ is the weight of **b** in the prior of **a** and as **a** cannot contribute to itself, $w_{\mathbf{a},\mathbf{a}} = 0$.

Mixing neighbouring literature to build a prior

The probability p_i that an article mentioning a metabolite also mention a disease is modelled with a Beta distribution, flexible and suitable for modelling proportions[41]. We assume that *a priori*, any metabolites and diseases are independent concepts in the literature, so that mention of the former does not affect the probability of mentioning the latter and $E[p_i] = P$. Under this assumption, for any contributor i, the prior distribution of p_i is modelled as a Beta distribution parameterized by mean $(\mu = P)$ and sample size (ν):

$$y_i | n_i, p_i \sim Bin(n_i, p_i) \tag{4a}$$

$$p_i \sim Beta(\alpha^{(0)}, \beta^{(0)}) \tag{4b}$$

$$\alpha^{(0)} = \mu\nu, \beta^{(0)} = (1 - \mu)\nu \text{ with } \mu = P \tag{4c}$$

The sample size ν is a hyperparameter and controls the variance, the higher ν, the lower the variance: $Var[p_i] = \frac{\mu(1-\mu)}{1+\nu}$. More intuitively, ν can be seen as the number of pseudo-observations that support this prior belief. Since $\mu = P$, a relationship would not be suggested *a priori* and the higher ν, the more each contributor i would have to bring new evidences (n_i) to change this prior belief[42]. As the Beta distribution is a conjugate prior of the Binomial distribution, the posterior distribution of p_i can also be expressed as a Beta distribution:

$$p_i | y_i, n_i \sim Beta(\alpha_i^{(1)}, \beta_i^{(1)}) \tag{5a}$$

$$\alpha_i^{(1)} = \alpha^{(0)} + y_i \text{ and } \beta_i^{(1)} = \beta^{(0)} + (n_i - y_i) \tag{5b}$$

For overlooked neighbours which might bring unreliable contributions, the posterior distribution of p_i acts as a shrinkage procedure, by adjusting the probability distribution toward the overall probability P of mentioning the disease. This is illustrated in Figure 1.D: the contributor f has only 2 annotated publications, with one mentioning the disease. While the raw estimated probability that f mentions the disease clearly seems overestimated due to its small ~~amount of available literature~~number of annotated articles, the posterior distribution of p_f is more reliable.
As illustrated in Figure 1.E, the prior distribution of p_k, also noted f_{prior}, is then defined as a mixture of the distributions $Beta(\alpha_i^{(1)}, \beta_i^{(1)})$ of each contributor, weighted by $w_{i,k}$:

$$y_k | n_k, p_k \sim Bin(n_k, p_k) \tag{6a}$$

$$p_k \sim \sum_{i \in T_k} w_{i,k} Beta(\alpha_i^{(1)}, \beta_i^{(1)}) \tag{6b}$$

In summary, the parameters α and ν respectively control the average distance to which a metabolite is allowed to contribute to the prior of its neighbours, and the strength of the initial prior in the shrinkage procedure. The impact of these parameters on the constructed prior and predictions is discussed in Supplementary ~~S4.1~~S4.3. In the analyses presented in sections *Suggesting relations with diseases for overlooked metabolites* and *Case study*, we set $\alpha = 0.4$ and $\nu = 1000$.

Updating prior and selecting novel associations

For the compound k, the final posterior mixture distribution of p_k, also noted f_{post} (Cf. Figure 1.F), is thus expressed as a mixture of the updated posterior distributions of each contributor, reweighted according to the observed data (n_k and y_k):

$$p_k | y_k, n_k \sim \sum_{i \in T_k} W_{i,k} Beta(\alpha_i^{(2)}, \beta_i^{(2)}) \tag{7a}$$

$$W_{i,k} = \frac{w_{i,k} C_{i,k}}{\sum_{i' \in T_k} w_{i',k} C_{i',k}} \tag{7b}$$

$$\text{with } C_{i,k} = \binom{n_k}{y_k} \frac{B(\alpha_i^{(2)}, \beta_i^{(2)})}{B(\alpha_i^{(1)}, \beta_i^{(1)})}, \ \alpha_i^{(2)} = \alpha_i^{(1)} + y_k \tag{7c}$$

$$\text{and } \beta_i^{(2)} = \beta_i^{(1)} + (n_k - y_k) \tag{7d}$$

$C_{i,k}$ represents the probability of observing the data (y_k, n_k) of the metabolite k, where p_k is drawn from the Beta distribution of the contributor i ($Beta(\alpha_i^{(1)}, \beta_i^{(1)})$), as in a Beta-binomial model. Therefore, the posterior weights in the mixture ($W_{i,k}$) correspond to the initial weights ($w_{i,k}$), reweighted according to the likelihood of the observations from the perspective of the contributor i.
From the mixture distribution, we evaluate the probability that $p_k \leq P$, or the posterior error that an article mentioning the metabolite k, would mention the disease more frequently than expected, noted *CDF*. We set $q = 1 - CDF$ and then use the log odds of q, such as $LogOdds = log(\frac{q}{1-q})$. Therefore, if $LogOdds > 0$, it is more likely that the metabolite k is related to the MeSH than it is not, and vice-verca. Also, we defined $Log_2FC = log_2(\frac{E[f_{post}]}{P})$. As *LogOdds* can lead to infinite values (if CDF wasn't precisely computed and approximated to 0), the Log_2FC can in turn provide a useful estimator to rank the relations. In turn, Log_2FC , being proportional to the mean $E[f_{post}]$, is much more sensitive to outlier contributors than *LogOdds*[43]. When evaluating predictions, *LogOdds* should be considered as a measure of significance and Log_2FC as a measure of effect size. Finally, *LogOdds* and Log_2FC can also be computed independently for each contributor i using their associated component in the prior ($Beta(\alpha_i^{(1)}, \beta_i^{(1)})$) and posterior mixture ($Beta(\alpha_i^{(2)}, \beta_i^{(2)})$).

Different scenarios

For metabolites mentioned in few articles and with literature available in the neighbourhood (2), the behaviour of the method is exactly as described above. When the compound k has no annotated articles (1), only the distribution f_{prior} is used to compute *LogOdds* and Log_2FC. In summary, for metabolites without literature, *LogOdds* and Log_2FC are derived from f_{prior}, while for metabolites with literature, they are obtained from f_{post}. For the latter, *priorLogOdds* and *priorLog*$_2$*FC* are computed from the prior distribution f_{prior} and aim to represent the belief of the metabolic neighbourhood, without the influence of the compound's literature.
There may be no literature available in the neighbourhood of some metabolites. In this case, the prior distribution is simply defined by $Beta(\alpha^{(0)}, \beta^{(0)})$ and then the posterior distribution is $Beta(\alpha_k^{(1)}, \beta_k^{(1)})$. In the worst-case, where no literature is available for the metabolite and its neighbourhood, the basic distribution $Beta(\alpha^{(0)}, \beta^{(0)})$ is used, but predictions are automatically discarded.
Since the construction of the prior from the neighbourhood's literature is critical in the proposed method, several diagnostic values are also reported to judge its consistency. Those additional indicators are detailed in Supplementary S1.3.

Availability of source code and requirements (optional, if code is present)

- Project name: Forum-LiteraturePropagation
- Project home page: https://github.com/eMetaboHUB/Forum-LiteraturePropagation
- Operating system(s): Platform independent
- Programming language: Python, bash script
- Other requirements: Python 3.7, Pip, Conda
- License: CeCILL 2.1

Availability of supporting data and materials

The data set(s) supporting the results of this article is(are) available in the https://github.com/eMetaboHUB/Forum-LiteraturePropagation repository.

Declarations

List of abbreviations

CysDA : Cysteinyldopamine
DOPAL : 3-4-dihydroxyphenylacetaldehyde
DOPAC : 3,4-dihydroxyphenylacetate
KG : Knowledge Graph
PCOS : Polycystic Ovary Syndrome
ROC : Receiver operating characteristic
RWR : random walk with restart

Consent for publication

Not applicable

Competing Interests

The authors declare that they have no competing interests

Funding

This project has received funding from the INRA SDN and the European Union's Horizon 2020 research and innovation program under grant agreement GOLIATH No. 825489. This work was supported by the French Ministry of Research and National Research Agency as part of the French MetaboHUB infrastructure (GrantANR-INBS-0010).

Author's Contributions

M. Delmas : Conceptualization, Formal Analysis, Investigation, Methdology, Software, Validation, Writing - original draft O. Filangi : Methodology, Software, Visualization, Writing - review & editing C. Duperier : Resources (System Administration) N. Paulhe : Software, Visualization (Web portal) F. Vinson : Software (Database and API) P. Rodriguez-Mier : Investigation, Methodology, Validation, Writing - review & editing F. Giacomoni : Funding acquisition, Projet administration, Supervision, Writing - review & editing F. Jourdan : Funding acquisition, , Investigation, Projet administration, Supervision, Writing - original draft C. Frainay : Conceptualization, Funding acquisition, Investigation, Methodology, Projet administration, Software, Supervision, Validation, Writing - original draft

Acknowledgements

We are very grateful to Juliette Cooke for proofreading the manuscript.

References

1. Piñero J, Ramírez-Anguita JM, Saüch-Pitarch J, Ronzano F, Centeno E, Sanz F, et al. The DisGeNET knowledge platform for disease genomics: 2019 update. Nucleic acids research 2020;48(D1):D845–D855. Publisher: Oxford University Press.
2. UniProt Consortium T. UniProt: the universal protein knowledgebase. Nucleic Acids Research 2018 Mar;46(5):2699–2699. https://academic.oup.com/nar/article/46/5/2699/4841658.
3. Wishart DS, Feunang YD, Marcu A, Guo AC, Liang K, Vázquez-Fresno R, et al. HMDB 4.0: the human metabolome database for 2018. Nucleic acids research 2018;46(D1):D608–D617. Publisher: Oxford University Press.
4. Mattingly CJ, Rosenstein MC, Colby GT, Forrest Jr JN, Boyer JL. The Comparative Toxicogenomics Database (CTD): a resource for comparative toxicological studies. Journal of Experimental Zoology Part A: Comparative Experimental Biology 2006 Sep;305A(9):689–692. https://onlinelibrary.wiley.com/doi/10.1002/jez.a.307.
5. Wishart DS, Bartok B, Oler E, Liang KYH, Budinski Z, Berjanskii M, et al. MarkerDB: an online database of molecular biomarkers. Nucleic Acids Research 2021 Jan;49(D1):D1259–D1267. https://academic.oup.com/nar/article/49/D1/D1259/6007662.
6. Delmas M, Filangi O, Paulhe N, Vinson F, Duperier C, Garrier W, et al. FORUM: building a Knowledge Graph from public databases and scientific literature to extract associations between chemicals and diseases. Bioinformatics 2021 Nov;37(21):3896–3904. https://academic.oup.com/bioinformatics/article/37/21/3896/6363786.
7. Bornmann L, Haunschild R, Mutz R. Growth rates of modern science: a latent piecewise growth curve approach to model publication numbers from established and new literature databases. Humanities and Social Sciences Communications 2021 Oct;8(1):1–15. https://www.nature.com/articles/s41599-021-00903-w, number: 1 Publisher: Palgrave.
8. Su AI, Hogenesch JB. Power-law-like distributions in biomedical publications and research funding. Genome Biology 2007;8(4):404. http://genomebiology.biomedcentral.com/articles/10.1186/gb-2007-8-4-404.
9. Edwards AM, Isserlin R, Bader GD, Frye SV, Willson TM, Yu FH. Too many roads not taken. Nature 2011 Feb;470(7333):163–165. http://www.nature.com/articles/470163a.
10. Wood V, Lock A, Harris MA, Rutherford K, Bähler J, Oliver SG. Hidden in plain sight: what remains to be discovered in the eukaryotic proteome? Open Biology 2019 Feb;9(2):180241. https://royalsocietypublishing.org/doi/10.1098/rsob.180241.
11. Pandey AK, Lu L, Wang X, Homayouni R, Williams RW. Functionally Enigmatic Genes: A Case Study of the Brain Ignorome. PLoS ONE 2014 Feb;9(2):e88889. https://dx.plos.org/10.1371/journal.pone.0088889.
12. Stoeger T, Gerlach M, Morimoto RI, Nunes Amaral LA. Large-scale investigation of the reasons why potentially important genes are ignored. PLOS Biology 2018 Sep;16(9):e2006643. https://dx.plos.org/10.1371/journal.pbio.2006643.
13. Perc M. The Matthew effect in empirical data. Journal of The Royal Society Interface 2014 Sep;11(98):20140378. https://royalsocietypublishing.org/doi/10.1098/rsif.2014.0378.
14. Miga KH, Koren S, Rhie A, Vollger MR, Gershman A, Bzikadze A, et al. Telomere-to-telomere assembly of a complete human X chromosome. Nature 2020 Sep;585(7823):79–84. http://www.nature.com/articles/s41586-020-2547-7.
15. Fiehn O. Metabolomics — the link between genotypes and phenotypes. In: Town C, editor. Functional Genomics Dordrecht: Springer Netherlands; 2002.p. 155–171. http://link.springer.com/10.1007/978-94-010-0448-0_11.
16. Kim S, Thiessen PA, Cheng T, Yu B, Shoemaker BA, Wang J, et al. Literature information in PubChem: associations between PubChem records and scientific articles. Journal of Cheminformatics 2016 Dec;8(1):32. https://jcheminf.biomedcentral.com/articles/10.1186/s13321-016-0142-6.
17. Lacroix V, Cottret L, Thebault P, Sagot MF. An Introduction to Metabolic Networks and Their Structural Analysis. IEEE/ACM Transactions on Computational Biology and Bioinformatics 2008 Oct;5(4):594–617. http://ieeexplore.ieee.org/document/4585358/.
18. Robinson JL, Kocabaş P, Wang H, Cholley PE, Cook D, Nilsson A, et al. An atlas of human metabolism. Science Signaling 2020 Mar;13(624):eaaz1482. https://stke.sciencemag.org/lookup/doi/10.1126/scisignal.aaz1482.
19. Hristov BH, Chazelle B, Singh M. uKIN Combines New and Prior Information with Guided Network Propagation to Accurately Identify Disease Genes. Cell Systems 2020 Jun;10(6):470–479.e3. https://linkinghub.elsevier.com/retrieve/pii/S2405471220301939.
20. Köhler S, Bauer S, Horn D, Robinson PN. Walking the Interactome for Prioritization of Candidate Disease Genes. The American Journal of Human Genetics 2008 Apr;82(4):949–958. https://linkinghub.elsevier.com/retrieve/pii/S0002929708001729.
21. Vanunu O, Magger O, Ruppin E, Shlomi T, Sharan R. Associating Genes and Protein Complexes with Disease via Network Propagation. PLoS Computational Biology 2010 Jan;6(1):e1000641. https://dx.plos.org/10.1371/journal.pcbi.1000641.
22. Frainay C, Aros S, Chazalviel M, Garcia T, Vinson F, Weiss N, et al. MetaboRank: network-based recommendation system to interpret and enrich metabolomics results. Bioinformatics 2019 Jan;35(2):274–283. https://academic.oup.com/bioinformatics/article/35/2/274/5050022.
23. Ghaderinezhad F, Ley C. On the Impact of the Choice of the Prior in Bayesian Statistics. In: Tang N, editor. Bayesian Inference on Complicated Data Rijeka: IntechOpen; 2020.
24. Newman ME. Power laws, Pareto distributions and Zipf's law. Contemporary physics 2005;46(5):323–351. Publisher: Taylor & Francis.
25. O'Dowd Y, Driss F, Dang PMC, Elbim C, Gougerot-Pocidalo MA, Pasquier C, et al. Antioxidant effect of hydroxytyrosol, a polyphenol from olive oil: scavenging of hydrogen peroxide but not superoxide anion produced by human neutrophils. Biochemical Pharmacology 2004 Nov;68(10):2003–2008. https://linkinghub.elsevier.com/retrieve/pii/S0006295204004551.
26. Monroy-Noyola A. Hydroxytyrosol inhibits MAO isoforms and prevents neurotoxicity inducible by MPP invivo. Fron-

tiers in Bioscience 2020;12(1):25–37. https://fbscience.com/Scholar/articles/10.2741/S538.
27. Brunetti G, Di Rosa G, Scuto M, Leri M, Stefani M, Schmitz-Linneweber C, et al. Healthspan Maintenance and Prevention of Parkinson's-like Phenotypes with Hydroxytyrosol and Oleuropein Aglycone in C. elegans. International Journal of Molecular Sciences 2020 Apr;21(7):2588. https://www.mdpi.com/1422-0067/21/7/2588.
28. Agarwal SK, Judd HL, Magoffin DA. A mechanism for the suppression of estrogen production in polycystic ovary syndrome. The Journal of Clinical Endocrinology & Metabolism 1996 Oct;81(10):3686–3691. https://academic.oup.com/jcem/article-lookup/doi/10.1210/jcem.81.10.8855823.
29. Xu XL, Deng SL, Lian ZX, Yu K. Estrogen Receptors in Polycystic Ovary Syndrome. Cells 2021 Feb;10(2):459. https://www.mdpi.com/2073-4409/10/2/459.
30. Matteri RK, Stanczyk FZ, Gentzschein EE, Delgado C, Lobo RA. Androgen sulfate and glucuronide conjugates in nonhirsute and hirsute women with polycystic ovarian syndrome. American Journal of Obstetrics and Gynecology 1989 Dec;161(6):1704–1709. https://linkinghub.elsevier.com/retrieve/pii/000293788990954X.
31. Song Y, Ye W, Ye H, Xie T, Shen W, Zhou L. Serum testosterone acts as a prognostic indicator in polycystic ovary syndrome-associated kidney injury. Physiological Reports 2019 Aug;7(16). https://onlinelibrary.wiley.com/doi/abs/10.14814/phy2.14219.
32. Consortium TECA, Ruth KS, Day FR, Tyrrell J, Thompson DJ, Wood AR, et al. Using human genetics to understand the disease impacts of testosterone in men and women. Nature Medicine 2020 Feb;26(2):252–258. http://www.nature.com/articles/s41591-020-0751-5.
33. Doldi N, Gessi A, Destefani A, Calzi F, Ferrari A. Polycystic ovary syndrome: anomalies in progesterone production. Human Reproduction 1998 Feb;13(2):290–293. https://academic.oup.com/humrep/article-lookup/doi/10.1093/humrep/13.2.290.
34. O'Reilly MW, Taylor AE, Crabtree NJ, Hughes BA, Capper F, Crowley RK, et al. Hyperandrogenemia Predicts Metabolic Phenotype in Polycystic Ovary Syndrome: The Utility of Serum Androstenedione. The Journal of Clinical Endocrinology & Metabolism 2014 Mar;99(3):1027–1036. https://academic.oup.com/jcem/article/99/3/1027/2537474.
35. Stener-Victorin E, Holm G, Labrie F, Nilsson L, Janson PO, Ohlsson C. Are there any sensitive and specific sex steroid markers for polycystic ovary syndrome? The Journal of Clinical Endocrinology and Metabolism 2010 Feb;95(2):810–819.
36. Haraldsdóttir HS, Thiele I, Fleming RM. Comparative evaluation of open source software for mapping between metabolite identifiers in metabolic network reconstructions: application to Recon 2. Journal of Cheminformatics 2014 Dec;6(1):2. https://jcheminf.biomedcentral.com/articles/10.1186/1758-2946-6-2.
37. Meiser J, Weindl D, Hiller K. Complexity of dopamine metabolism. Cell communication and signaling: CCS 2013 May;11(1):34.
38. Goldstein DS, Jinsmaa Y, Sullivan P, Holmes C, Kopin IJ, Sharabi Y. 3,4-Dihydroxyphenylethanol (Hydroxytyrosol) Mitigates the Increase in Spontaneous Oxidation of Dopamine During Monoamine Oxidase Inhibition in PC12 Cells. Neurochemical Research 2016 Sep;41(9):2173–2178.
39. Nisenblat V, Norman RJ. Androgens and polycystic ovary syndrome. Current Opinion in Endocrinology, Diabetes & Obesity 2009 Jun;16(3):224–231. https://journals.lww.com/01266029-200906000-00006.
40. Poupin N, Vinson F, Moreau A, Batut A, Chazalviel M, Colsch B, et al. Improving lipid mapping in Genome Scale Metabolic Networks using ontologies. Metabolomics 2020;16(4):1–11. Publisher: Springer.
41. Ferrari S, Cribari-Neto F. Beta regression for modelling rates and proportions. Journal of applied statistics 2004;31(7):799–815.
42. Kruschke J. Doing Bayesian data analysis: A tutorial with R, JAGS, and Stan. Boston: Academic Press; 2014.
43. Yang J, Rahardja S, Fränti P. Outlier detection: how to threshold outlier scores? In: Proceedings of the international conference on artificial intelligence, information processing and cloud computing; 2019. p. 1–6.

Click here to access/download
Supplementary Material
Supp_revised.pdf

Clément Frainay, Ph.D
INRAE ToxAlim Research centre in food toxicology
180 Chemin de Tournefeuille, 31300 Toulouse, France

Nicole Nogoy, Ph.D
GigaScience
www.gigasciencejournal.com

June 13, 2023

Dear Nicole Nogoy,

In response to your communication dated March 16th, we have edited our manuscript GIGA-D-23-00014 titled "Suggesting disease associations for overlooked metabolites using literature from metabolic neighbours". We believe that the quality of the proposed manuscript has improved thanks to the editors and reviewer's comments and suggestions, and hope that it will be suited for the readership of GigaScience.

Please find attached our response to reviewers' comments.

Sincerely,

Clément Frainay

Response to Reviewers

Reviewer #1:

Manuscript Number: GIGA-D-23-00014, entitled, " Suggesting disease associations for overlooked metabolites using literature from metabolic neighbours", and submitted to the journal: GigaScience, applied 'guilt by association' principle to literature information for "understudied metabolites" by using a Bayesian framework. It is an interesting manuscript, an active area of research and would have an interest in the metabolomics research community. However, this reviewer would like to help improve the manuscript and scope of the work with the following suggestions:

1.1 A list/ DB of all such "overlooked metabolites" and their chemical class distribution/ ChemRICH sort of enrichment would help the readers capture the correct information.

The authors thank the reviewer for bringing this suggestion. The complete list of overlooked metabolites (2113 species) have been added on the GitHub repository and in the FTP server. Since overlooked metabolites can have limited annotations in standard chemical ontologies such as Chebi or MeSH, we decided to use ClassyFire. ClassyFire provides an automatic hierarchical classification of molecules based on structural descriptors such as inchiKey identifiers. We managed to obtain an InchiKey for 1180 (approximately 56%) out of the total 2113 metabolites considered as overlooked in the metabolic network using their annotation in MetaNetX. Subsequently, we analyzed the distribution of the superclass to which these metabolites are classified by ClassyFire. SuperClasses are generic categories of compounds that we can use to get an estimation of the composition of chemical families in the set of overlooked metabolites for this metabolic network. From this sample, it can be estimated that the majority of metabolites considered as overlooked in this metabolic network are actually "Lipids and lipid-like molecules" (e.g: Fatty Acyls, Sphingolipids, etc.), a class with a strong compositional complexity. However, this observation is based on a limited sampling of metabolites within a specific metabolic network. As a result, this subset is unlikely to be representative and while it could give some insights, we argue that it could

lead to misleading interpretations and decided to not add this directly in the article.

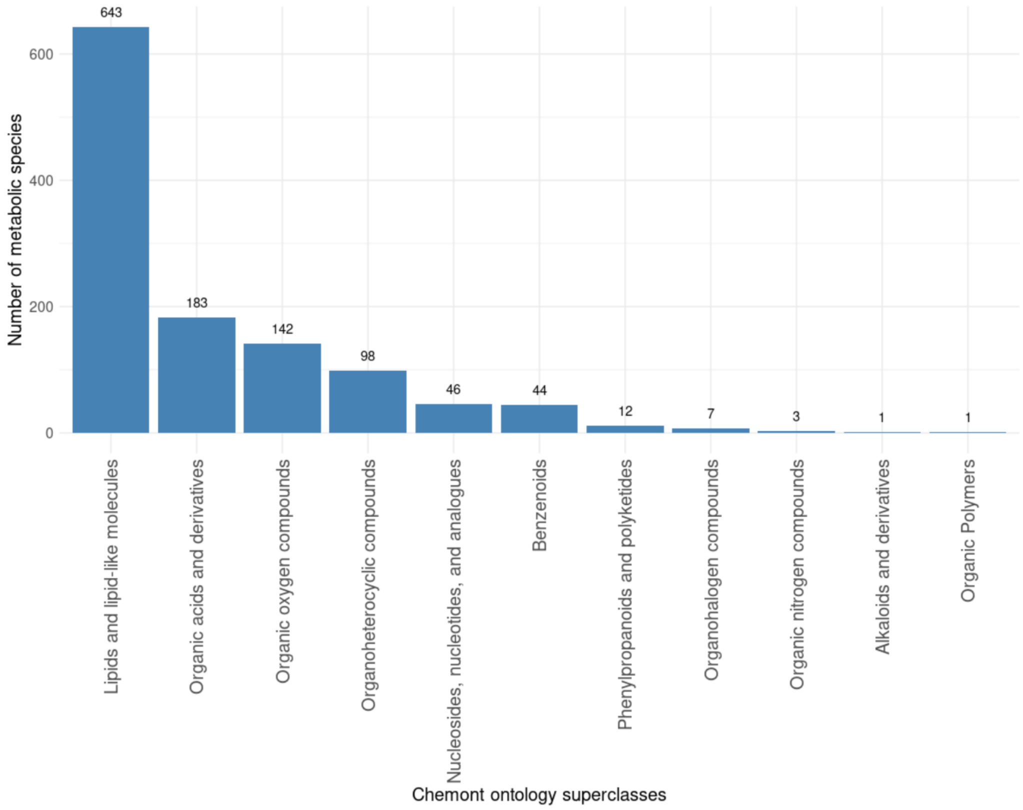

F1: Distribution of the chemical superclass obtained with ClassyFire for the 1180 overlooked metabolites with an available structural representation (InchiKey) in the metabolic network.

1.2 How did/ would the tool perform with "very well known metabolites" for example say, phenylalanine or proline or citric acid ?

We thank the reviewer for this interesting remark. The behaviour of the method for "very well known metabolites", as opposed to overlooked metabolites, is quite straightforward in the Bayesian settings. The impact of the prior on the predictions will vanish as the literature of the targeted compound increases. The *LogOdds* estimator then tends to infinity, while Log2FC tends to its exact value when estimated only from the literature of the compound. For instance, among the 278,277 articles discussing the glucose in FORUM, 24.839 co-mentioned the *Diabetes type 2* MeSH descriptor. The prior and posterior distributions obtained for this relationship are presented below. The posterior distribution is solely driven by the literature of the glucose which, being much larger than that of its contributors, completely erases the information brought by the prior. The distributions of the contributors in the posterior mixture are therefore centred around the co-mention frequency of the glucose and Diabetes type 2 (≈ 0.089). Thus, although the proposed approach can be applied to these well-known metabolites, the predictions are insensitive to the built prior which is nevertheless at the core of this method. In this case, the relationships would be as well evaluated with a classic over-representation analysis.

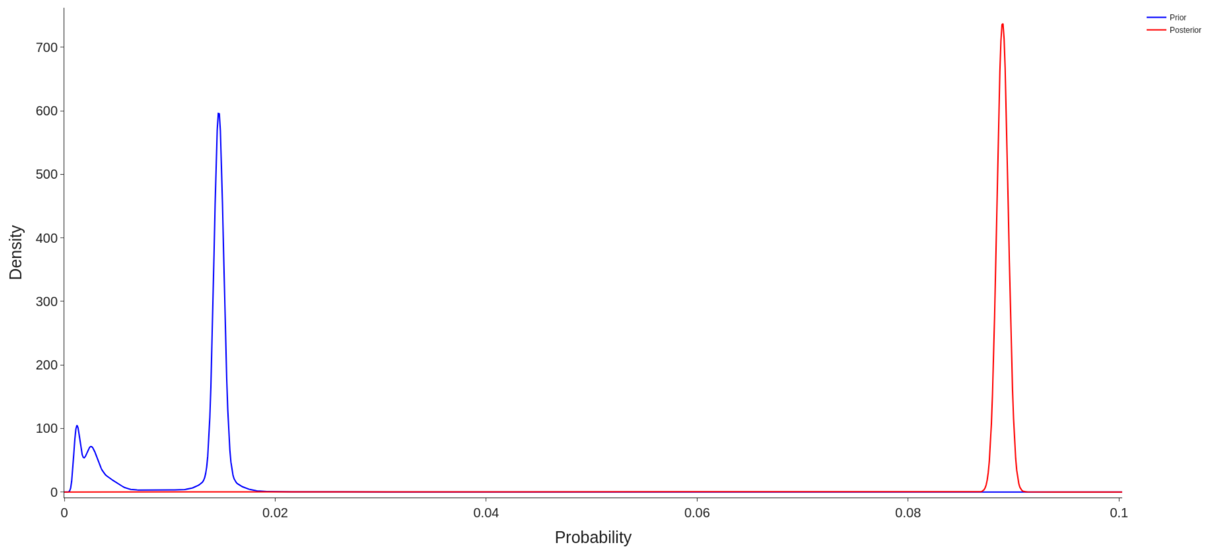

F2: Prior (blue) and posterior (red) distributions for the relation between Glucose and Diabetes type 2.

In addition to the aforementioned extreme example, a similar phenomenon can be observed through an example proposed by the reviewer: *Phenylalanine* (specie id M_m02724c) and the MeSH descriptor *Phenylketonurias* or PKU (D010661). PKU represents a group of disorders caused by a deficiency in the production of phenylalanine hydroxylase, and for which the dosage of phenylalanine is the standard diagnostic method. Again, the posterior distribution eliminates any information from the prior and is centred around 0.0107, which is the expected probability that an article mentioning phenylalanine also mentions the disease. Indeed, out of the 28.507 articles mentioning Phenylalanine, 3.045 are annotated with the MeSH term PKU.

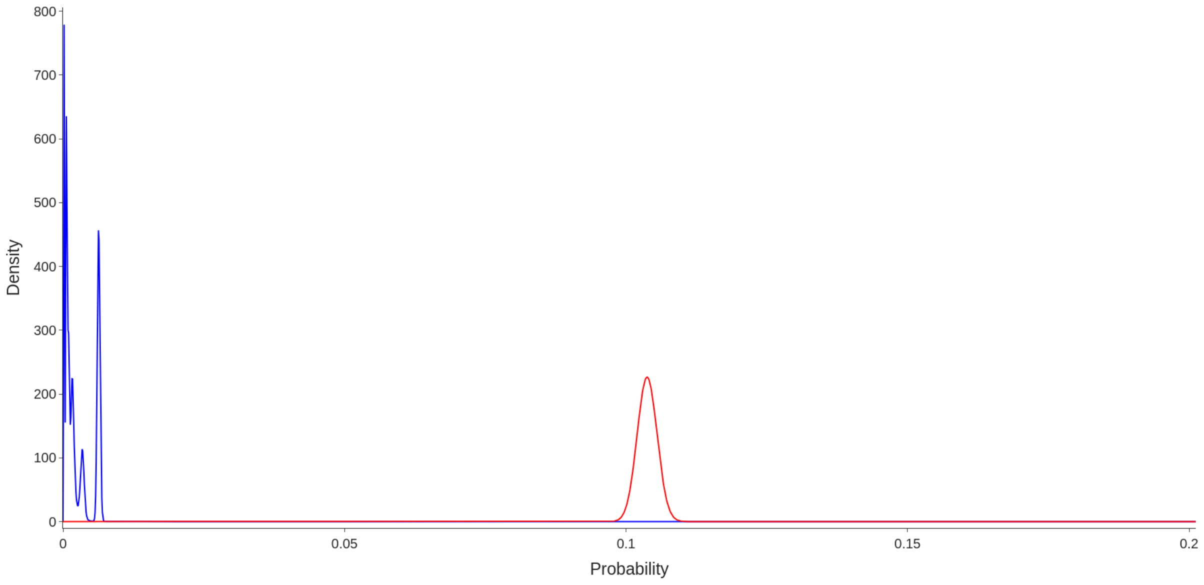

F3: Prior (blue) and posterior (red) distributions for the relation between Phenylalanine and Phenylketonurias (PKU).

1.3 How does one check for "literature / reporting biases" for the highly reported vs lowly reported metabolites in the manuscripts ?

From our understanding, hoping we interpret correctly reviewer comment, this check would be related to the retrieval of metabolites' mentioning articles. We hope that the following information can answer your question:
There are several ways one can access the literature of metabolites described in this manuscript. First, all the data are publicly available in the git repository https://github.com/eMetaboHUB/Forum-LiteraturePropagation where *uncompress_species_pmids_Human1_1.7.csv* contains the number of annotated articles for each of the 2704 species in the pruned version of Human1 metabolic network. *If one desires to recover the list of PubMed identifiers behind these frequency values,* the FORUM KG (https://forum-webapp.semantic-metabolomics.fr) is the most direct way of recovering the original set of articles mentioning a metabolite. However, as this extraction requires querying the SPARQL endpoint, which we acknowledge is difficult for non-familiar users, we would recommend accessing it individually for each compound from their PubChem page or directly on PubMed.

1.4 Does this approach distinguish for targeted vs untargeted metabolomics paper based hits ?

We thank the reviewer for this interesting remark. Although we could increase the confidence of hits from targeted analyses compared to untargeted using some weighting policies (or using Metabolomics Standard Initiative classification for metabolite identification), the main challenge would lie in accurately extracting this information. In fact, articles related to metabolomics analyses are not yet indexed in PubMed with a precise MeSH term to distinguish the two types of approaches. Determining this from the title or abstract would also require building a classification model for which training data are not available. More generally, proposing a different weighting for the contribution of each article according to different factors (type of analysis, date, etc.), so that they are not all considered equivalently, is indeed an interesting perspective for future works.

1.5 "Overlooked metabolites" need to be defined well, upfront for clarity.

We are grateful to the reviewer for pointing out this lack of clarity. We propose to modify the end of the first paragraph of the Method section: "*In this study we define a set of overlooked compounds as compounds with less than 100 retrieved mentioning article, which correspond to orders of magnitude below 4,799, the mean number of retrieved articles per compound (when any), and is close to the median number of articles, 172. It is worth mentioning that such threshold serves solely as a prioritization criterion, since the method applicability is not restricted to a given range of mentioning corpus sizes (although its relevance is less obvious when a sufficient corpus is already available).*"

1.6 large fraction of metabolites are rarely or never mentioned in the literature: What is a good estimate from the authors? A numerical value would be informative here.

While our results from the metabolic network clearly suggest that a large fraction of metabolites are overlooked, we argue that this information, although reflecting a reality in the field, cannot be used to propose a reliable estimate. This estimator would be biased by various factors and in the first place, the lack of external identifiers (e.g. CID) that connect to the literature. Additionally, the purpose of the metabolic network is not to provide an exhaustive map of the metabolism and some parts (e.g lipid metabolism are often reduced to generic classes). Nonetheless, our estimate based on the whole PubChem database seems more reliable and we decide to put the emphasis on it in the abstract to provide a numerical value. We therefore reworked the abstract by adding the following sentence: "*However, we show that the vast majority of compounds (> 99\%) in the PubChem database lack annotated literature. This dearth of available information can have a direct impact on the interpretation of metabolic signatures, which is often restricted to a subset of significant metabolites}*."

1.7 Too many terms used does not help: overlooked metabolites vs. understudied metabolites and so on. Please use a singular term for consistency.

We thank the reviewer for helping us improve the readability of the manuscript. We replaced every mention of "understudied" with "overlooked".

1.8 Method and data description section is too wordy, need to be shortened and need to use mathematical expressions whenever applicable.

We appreciate the feedback regarding the "Method and data description" section of our article and we acknowledge that this section may be too wordy and lacking in mathematical expressions. We made some improvements and tried as much as possible to reduce the size of this section.
We made this choice given the potential readership of the work. We anticipate that some readers wishing to use the provided associations to interpret their results, may not have a strong mathematical background. Therefore, while the use of mathematical expressions would shorten the section, it could also be a barrier to its understanding and discourage some readers. We have strived to make our methodology as accessible as possible by providing two descriptions, which we believe will complement each other. Our primary focus in the "Method and Data description" section is to provide an intuitive and concise overview of the main steps of our approach, avoiding the use of mathematical expressions.
Simple expressions have been added to this section according to the various reviewers' comments in order to remove potential ambiguities. In addition, a complete description with all the mathematical details is provided at the end of the manuscript in the method section for the interested readers.

Reviewer #2:

Overall Notes
This work is innovative and will provide an important contribution to the computational metabolomics field. The experiments and methodology are well-designed and executed, and the software is also well-documented. That being said, the structure and writing of the manuscript needs to be reworked. There are several areas of the text where descriptions are unclear, detailed below. Some of the text is also out of order, e.g. weights are shown in a figure before they are defined, and TPR and FPR are reported without describing the dataset. Finally, there are several Supplementary experiments that are never mentioned in the main text. At least a brief description of these should be given in the main text and then the Supplementary referenced.

2.1 Abstract

Some of the language used here is difficult to read or unclear. In particular:

2.1.1 I believe you mean to say that signatures… "have a strong added value", not "are a strong-added value".

We corrected this in the manuscript.

2.1.2 "we extend the 'guilt by association' principle to literature information by using a Bayesian framework". This is vague. Instead, briefly explain how you use a Bayesian framework to determine guilt by association.

We reworked the abstract and specifically added the following sentence to briefly illustrate the intuition behind the prior and the Bayesian framework in the context of the *guilt by association* principle: "*The underlying assumption is that the literature associated with the metabolic neighbours of a compound can provide valuable insights, or an a priori, into its biomedical context.*"

2.1.3 "1,047 overlooked metabolites". Do you mean metabolites not in the literature?

Not exactly, we meant metabolites which are rarely mentioned in articles (< 100 annotated articles), so they almost never mentioned in the literature. As this notion of "overlooked" metabolites is key in this article, it has also been clarified in section "Method and data description" according to the Reviewer 1 comments.

2.1.4 Your method uses knowledge about metabolic interactions/reactions to generate the graph, but this is not mentioned at all in the abstract. The abstract should explain that this knowledge is being used and describe how it is complementary to the literature.

Following the previous comments and the addition of the underlying hypothesis in the abstract, we also decided to add the following sentence to emphasize the role of the metabolic network in defining the structure of the graph used to propagate information: "*The metabolic neighbourhood of a compound can be defined from a metabolic network and correspond to metabolites to which it is connected through biochemical reactions.*"

2.2 Background

2.2.1 it is irrelevant to mention exponential growth. This detracts from the main point, which is the imbalanced knowledge distribution.

We removed this part of the sentence from the manuscript.

2.2.2 "This topic has received much attention for genes and proteins…" Can you provide some citations?

The references related to this statement were provided in the next sentence ("*Consequently, [...] gene annotations in databases*"). According to this reviewer comment, we decided to move them upstream.

2.2.3 "has an impact on the quantity and quality of gene annotations in databases" - in what way? Can you be more specific?

We wanted to highlight that the skewed distribution of the number of bibliographic references across genes is also reflected in the distribution of functional annotations in databases, such as Gene Ontology. See for instance between TP53 and ANKRD52. As it doesn't bring much more details for the rest of the article and could distract the reader, we decided to remove this sentence.

2.2.4 The first sentence of the second paragraph can be removed.

This sentence has been removed according to the reviewer's suggestion.

2.2.5 You discuss the issue of inaccurate identification as being related to the number of articles mentioning a compound. I feel that these are two separate issues. The first is related to identification, and the second is related to discussion of identified metabolites in the literature. The section regarding identification should be removed.

This section has been removed according to the reviewer's suggestion.

2.2.6 "Guilt by association principle", not hypothesis.

This has been corrected.

2.2.7 "The method returns several predictors to evaluate whether a significant proportion of the articles mentioning a metabolite would also mention a disease." Do you mean to say that the predictors predict whether or not the metabolite is related to the disease?

Indeed, by indicating whether a significant proportion of the articles mentioning a metabolite would also mention a disease, these predictors are meant to highlight a potential relation. We acknowledge that this could be expressed more explicitly in the background section, leaving this interpretation for the methodology section. We reworked this sentence accordingly.

2.2.8 You mention that you used FORUM Knowledge Graph to obtain your metabolite-disease associations. What about the metabolic neighborhoods? You should explain where these were obtained.

The metabolic neighbourhoods are defined from the Human 1 (v1.7) metabolic network, which was also pruned from spurious connections using an atom-mapping procedure. While we keep the details apart from the main text, we reworked the following sentence: “*Metabolic neighbourhoods were defined from the Human1 metabolic network and co-mention data between metabolites and diseases were extracted from the FORUM Knowledge Graph (KG)*”.
The details of the pre-processing step on the metabolic network and its implication of the results are detailed in Supplementary materials (S1.1, S4.5) and referenced in *Method and Data Description.*

2.3 Method and Data Description

2.3.1 How do you define "rarely mentioned"? Is there a cutoff criteria used?

We thank the reviewer for this interesting remark, which has also been highlighted by the other reviewers. We believe that the modifications applied to the first paragraph of the method section should clarify what we meant by "overlooked" or "rarely" mentioned metabolites, both conceptually and practically.

2.3.2 Does "amount of literature" mean number of articles?

Yes, we reformulated the formulation "amount of literature" everywhere in the article to bring clarity, as suggested by this reviewer.

2.3.3 How is "far distant" defined? It seems that you mean to say that one metabolite's influence on another decreases as the number of reactions separating them increases. Is this correct?

We thank the reviewer for pointing out this lack of clarity. Indeed, we make the assumption that the influence of a metabolite on another decreases as the number of reactions separating them increases. In order to consider all the potential paths connecting two metabolites in the network, we use the stationary probabilities from random walks starting from the former and reaching the latter as a measure of this distance.

However, when we referred to “far distant” metabolites in the sentence: “We impose that a metabolite can't influence its own prior or the prior of *far distant* metabolites.”, we inaccurately referred to metabolites whose probability of being reached during the random walk are below a predefined threshold. This concerns, for instance, metabolites that belong to different regions of the metabolic network. This constraint prevents influential metabolites (tryptophan, glucose, etc.) from sharing the articles mentioning them with metabolites that are unlikely to be involved in the regulation of common metabolic pathways.

Since this detail is explained thoroughly with mathematical expressions in section "*Estimating the contributions of metabolic neighbours*" in Method for interested readers and is not crucial for the understanding of the approach as a whole, we have chosen to exclude it from the method summary. The Figure 1 has also been updated accordingly.

2.3.4 Show Figure 1 as soon as it is mentioned. This goes for the other figures as well.

Indeed, the initial position of the figure was not ideal to follow the corresponding method in the main text, so we moved it one page closer.

2.3.5 You should explain more about the shrinkage procedure here. It isn't clear what you mean.

We are thankful to the reviewer for helping us to make this paper more clearer, particularly for the shrinkage step which is a key element in the presented approach. While the idea of shrinkage is also used for penalized regression, in this manuscript we refer to its applications in Bayesian settings. In this framework, the posterior mean distribution is shrunk towards the prior mean (μ), resulting in a more reliable estimator than the maximum likelihood estimator (MLE) for low sample sizes. This is illustrated in equations *5a* and *5b* when we show the posterior distribution of p_i. The parameterization of the prior beta distribution involves determining μ, assuming that metabolites and diseases are independent concepts in the literature, and setting the sample size (v) as a hyperparameter to control the strength of the prior.

We decided to modify the corresponding paragraph in the method summary section according to the reviewer’s comments : “*This results in a small sample size available to estimate the probability that an article mentioning* ***f*** *also mention the disease, which may lead to unreliable and spurious contributions. To address this, a shrinkage procedure is applied to all contributors, assuming that a priori, mentioning a metabolite in an article does not affect the probability of mentioning a particular disease. In Bayesian settings, a shrinkage estimator integrates information from the prior to readjusted raw estimates,*

reducing the effect of sampling variations (further details in section Mixing neighbouring literature to build a prior in Methods)."
We also redirect the interested reader to the Method section for the mathematical details.

2.3.6 In Figure 1C, there appears to be a stack of papers in a pink box in both the numerator and the denominator. What does this mean?

We thank the reviewer for pointing out this unclear illustration. This pink box represents the number of papers shared by **b** that reached the target compound **a**. This quantity is noted $t_{b,a}$. In Figure 1.C, we illustrated the simple computation of the weight of **b** in the prior of **a** (noted $w_{b,a}$) as the fraction of articles that reached A ($t_{b,a} + t_{c,a} + t_{e,a} + t_{f,a}$) that was send by **b** ($t_{b,a}$). To avoid any other ambiguities in this illustration, we explicitly annotate all the paper box with their corresponding value (e.g; $t_{c,a}$ or $w_{b,a}$) both in 1.B and 1.C.

2.3.7 "Then, we build the prior distribution for A, by mixing the probability distributions of each contributor (see Figure 1.E) according to their weights estimated in the previous step (Figure 1.C)". This is the first time you mention weights. You need to describe what the weights correspond to and how they are calculated first.

We apologize for adding this ambiguity. We reworked this section, both at the first mention of the weights and in the highlighted sentence. We also added a more explicit mention of the weights associated with the contributors using simple mathematical expressions: "*We refer to **b**, **c**, **e** and **f** as the contributors to the prior of **a**. Each contributor has a weight w in the prior of **a** (e.g $w_{b,a}$) proportional to its contribution.*" We also add a reminder in the following sentence: "*Then the prior distribution of **a** is built as a mixture of the probability distributions of individual contributors (**b**, **c**, **e** and **f**) as illustrated in Figure 1.E. Recall that the weight of each contributor in the mixture is ($w_{.,a}$), as estimated in the previous step (see Figure 1.C).*"

2.3.8 "Then, we build the prior distribution for A, by mixing the probability distributions of each contributor (see Figure 1.E) according to their weights estimated in the previous step (Figure 1.C)". This sentence is unclear. Please revise.

We reformulate this sentence according to the previous comment. Please, see the previous answer.

2.3.9 "Finally, several diagnostic values such as Entropy allow to assess the composition of the built prior (See Supplementary S1.3)". Either name all of the diagnostic values here or move Entropy to the supplementary and out of the main text.

As suggested by the reviewer, we removed the mention of Entropy in this sentence.

2.3.10 "Entropy evaluates the good balance of contributions in the prior. The more metabolites contribute to the mixture and the more their weights are uniformly distributed, the higher the entropy." This is not a clear explanation. Please explain mathematically what entropy is and what it represents here.

According to the last two comments of this reviewer, we reworked this paragraph. We decided to focus more on the applications and purposes of these diagnostic indicators rather than their formal definitions, which we decided to keep for the supplementary materials for the interested readers. We therefore replace the sentence relative to *Entropy* to a broader description of the purposes of the diagnostic indicators: "Finally, given its primary role in driving predictions, assessing the composition of the constructed prior is crucial. Essentially, the more contributors to the prior, close to the target compound, with balanced weights, the better it captures the neighbourhood literature and increases the confidence in predictions. To aid in this evaluation, a set of diagnostic indicators is presented in Supplementary S1.3".

However, as *Entropy* is the only diagnostic indicator used in the main text (for filtering the predictions), we also added a more formal definition directly where it is mentioned in section "Suggesting relations with diseases for overlooked metabolites". See comment 2.6.3.

2.4 Analyses: Unbalanced distribution of the literature related to chemical compounds

2.4.1 At the end of the first paragraph, it should be "is cumulatively less than the literature associated with glucose…"

This has been added to the manuscript.

2.4.2 Can metabolites without a CID be found in the metabolic network? If not, then you should not discuss those metabolites with an unannotated CID.

With the exception of the pruning process carried out during the construction of the carbon skeleton graph and described in S1.1, no metabolites were excluded because of a lack of annotations. All metabolites without an annotated CID are conserved in the metabolic network.

2.5 Analyses: Evaluation of the prior computation

2.5.1 "and is set to α = 0 for the direct neighbourhood and α = 0.4 for a larger one". Are these the only two values you consider? If so, why? How large is "larger"? This should also go in the methods section.

We thank the reviewer for pointing out this lack of clarity. An extensive analysis was actually performed on the impact of the both parameters α and ν on the performances and the composition of the prior in S4.3 Damping factor α and theoretical sample size ν: benchmark. We evaluated values for α in the set: [0, 0.1, 0.2, 0.3, 0.4, 0.5, 0.6, 0.7, 0.8, 0.9, 0.99] and for ν in the set [1, 10, 100, 1000, 10000, 100000 , 1000000]. From the performed analyses, we found that α=0.4 and ν=1000 appears to be reasonable setting, both in terms of composition of the built prior and balance between sensibility and precision. Finally, we also argued that there are no global optimal settings and recommend $0 \leq \alpha \leq 0.7$ and $1 \leq \nu \leq 10000$. We reworked this paragraph to point to these details in the supplementary materials when referring to the chosen values of α: "We therefore focused on two specific settings: α=0, where solely the direct neighbours contribute to the prior, and α=0.4, where contributions between direct or indirect neighbours are relatively balanced. The impact of the parameter α on the construction of the prior and the Precision-Recall tradeoff was extensively evaluated in Supplementary Material S4.3."

2.5.2 "All tested approaches outperform Baseline-Freq, showing the benefit of examining the neighbouring literature." This experiment needs to be explained in the main text. How did you define TPR and FPR?

Indeed, the evaluation results with the ROC curves were not explicitly presented in the main text and we decided to rework this paragraph to add "*The evaluation results on the validation dataset for all described approaches are presented in Figure 3.*". We also add a description of TPR and FPR in the legend of Figure 3: "*A true positive represents an association between a compound and a MeSH term which is both retrieved from the compound's mentioning corpus using Fisher Exact Test, and using methods in which no knowledge of such corpus is available. A false positive is only retrieved from the latter.*"

2.6 Analyses: Suggesting relations with diseases for overlooked metabolites

2.6.1 "However, by re-evaluating these predictions using a right-tailed Fisher exact Test (BH correction and selecting those with q.value <= 0.05), we found that ≈ 50% of them (925) would not have been found significant". Can you please explain this experiment further?

We thank the reviewer for pointing out this lack of clarity. With this analysis we wanted to emphasize the proportion of associations that would not have been highlighted by a standard approach, thus without considering the neighbourhood information. The right-tailed fisher exact test is a standard and robust approach for over-representation analysis of associations in the literature that we already applied at large scale in the first version of FORUM. We therefore used this same workflow to test all associations between metabolites

and diseases based on their co-mention frequency from the literature available in the metabolic network. We reformulate this paragraph and directly refer to the analysis done in the FORUM article: "*However, by re-evaluating them using the same workflow as in FORUM [FORUM 2021] (a standard over-representation analysis (ORA) using right-tailed Fisher exact Test, BH correction and threshold on q.value <= 0.05), we found that ~50% (925) of these associations would not have been highlighted.*"

2.6.2 "These relations are still weakly supported, nevertheless, our method showed that they are consistent with the neighbourhood." What does this mean?

We apologize for this lack of clarity. Despite these relationships being supported by only a few articles, the proposed approach showed that these are consistent with the literature of metabolic neighbours. These limited mentions could therefore be significant and deserve consideration. By using a standard ORA for comparison purposes, we also wanted to emphasize that the current volume of articles supporting a relation in the literature may simply not be sufficient (quantitatively) to effectively highlight it with this type of approach. We reworked the sentence as follows: "While only a few articles support these relationships and half of them were discarded by a standard ORA, the method showed their consistency with the literature of metabolic neighbours".

2.6.3 Why do you want high entropy in Table 1? This isn't clear if the reader doesn't know what entropy refers to here.

As we removed the definition of *Entropy* in the method summary (see comment 2.3.9 and 2.3.10), we reworked this paragraph and added both an introduction oriented on the application of this indicator and a definition that we hope clearer. We replaced the paragraph "*We also retained predictions based on well-balanced contributions from the neighbourhood by filtering on the diagnostic indicator Entropy > 1 (See details in Method and Supplementary S1.3).*", by: "*Predictions for which the prior was biased toward one dominant contributor and thus failed to capture the neighbourhood literature, were excluded by filtering on the diagnostic indicator Entropy > 1. Entropy is the Shanon entropy computed on the contributors weights in the prior: the more contributors with balanced weights, the higher the entropy. (See details in Method and Supplementary S1.3).*" We again direct the reader to the Supplementary materials.

2.7 Limitations

2.7.1 "Although we kept it in our analysis for sake of exhaustively…" It is not clear what this means.

We apologize for the lack of clarity. We wanted to emphasize that despite influential compounds like ethanol could provide out-of-context relations, we did not apply a filter to try to exclude them. We reworked this sentence: "*To avoid arbitrary filtering, we left to the user the choice to keep associations with such compounds after review*".

2.7.2 Methods Your equations are not numbered correctly. Your equation (1) should be equation (2). It looks like you have 14 equations total, but only one is numbered.

All the equations have been numbered in the new version of the manuscript.

2.7.3 Settings

2.7.3.1 How did you choose the cutoff in equation (1)?

As the probability to be reached by a random walk depends on the shortest distances within a network, we define a threshold that scales with the size of the network. We set the threshold to $1/(n - 1)$, which is the probability that a metabolite would be randomly chosen among all potentially reachable metabolites. It is important to note that this threshold is a default value and can be changed when calling the script to compute the associations (option `-q: The tolerance threshold).`

2.7.3.2 "These aspects are illustrated in Figure 1.B: B…" This entire paragraph should be moved up to the Method and Data Description section.

The paragraph has been reworked and an equivalent description is provided in section “Methods and Data description”. Nevertheless, we believe that even in this more technical description of the method, providing a link with the illustration in Figure 1.B may help to capture the behaviour of the method.

2.8 Mixing neighbouring literature to build a prior

2.8.1 Explain what the Beta distribution parameters mean in the context of this study in detail and why you chose a Beta distribution.

Being defined in the interval [0,1], the Beta distribution is a suitable model for modelling proportion, which is precisely what we want to estimate: the proportion (or probability) of articles mentioning a metabolite, that also mention a disease. Secondly, the beta distribution is the conjugate prior of the Binomial distribution, modelling the number of observed successes in a sequence of n trials, which is also the type of data that we have: among n_i articles mentioning a metabolite, y_i (successes) mention a disease. From this, the Beta-binomial model appears as a suitable framework for the purpose of this work.

When building the prior, the essential assumption is that *a priori* a metabolite and a disease are independent concepts in the literature. For all metabolites in the network, we start by modelling their prior probability of mentioning the disease with a Beta distribution parameterized under this hypothesis: the average probability equals the overall probability P of mentioning the disease in an article. The Beta distribution is therefore parameterized by mean μ and sample size ν, which determine the values of the two shape parameters α and β. μ being fixed to P, ν is actually the only hyperparameter setting the initial prior. Also, since μ is set to P, this default prior would not suggest a relation using LogOdds or Log2FC. Additionally, ν is related to the amount of evidences in the literature needed to change this prior belief. Thus, one should not directly interpret the values of α and β, as the real fixed

parameters are μ and ν. More explanations have been added to the method section regarding the Beta distribution and the implication of the parameters.

2.9 Updating prior and selecting novel associations

2.9.1 "In turn, Log2FC is much more sensitive to outlier contributors than LogOdds". Please provide a citation for this.

This is a common problem with outliers when an estimator is based on a mean, we reworded this sentence to highlight this and provided a reference for this statement in the manuscript.

2.10 References

2.10.1 Check the formatting for #27. It is overlapping the text in the right-hand column.

This has been fixed.

2.10.2 If you're going to discuss the Pareto Principle (28), try to find a review article that describes this principle rather than referencing a textbook.

This reference comes from a collection of commissioned introductory review articles and is highly cited; we believe it is appropriate in this context, and sufficient to grasp the concept needed to understand this section.

2.11 Supplementary Material

2.11.1 S1.3 Diagnostic Values

Here, it is clear what you mean by Entropy and why you want the value to be high rather than low. This should go in the main text. However, it is concerning that the meaning of the entropy cutoff changes with respect to the number of contributors. Consider using a weighted metric that has the same meaning regardless of the number of contributors.

We apologize for any confusion caused by the provided numerical examples. The meaning of Entropy remains the same regardless of the number of contributors. By considering a weighted metric that is normalized by the number of contributors, the reviewer may be referring to normalized entropy, also known as Efficiency, which is the observed Entropy divided by the maximal entropy (log2(N)).
However, imposing a fixed proportion of maximum entropy regardless of the number of contributors is not reasonable. For example, reaching 50% of the maximal entropy is easier when there are 5 contributors than when there are 50. Therefore, our choice aims to maintain a balanced distribution of contributors, becoming more flexible as the number of contributors increases. To address this, we set the threshold for Entropy at 1. This means that when there are only 2 contributors, the maximum entropy is required, and as the number of contributors increases, we progressively relax this constraint on a logarithmic

scale. For example, with 5 contributors 50% of the maximum entropy is required, for 10 contributors 30%, for 30 contributors 20%, for 100 contributors 15%, etc. We reformulated the corresponding paragraph in the supplementary materials. Finally, the selected threshold is a recommendation for the specific analysis we conducted where we aimed for stringency and users are free to set their own threshold according to their needs.

2.11.2 S2. Supplementary Tables

Why are your LogOdds values infinite? If this is an issue with taking the log of 0, then you should set a cutoff such that the values do not go to infinity.

The posterior error that an article mentioning the metabolite *k*, would mention the disease more frequently than expected is noted CDF and corresponds to $P(p_k < P)$. As CDF tends to 0 for strong relationships, *logOdds* will logically tend to infinity. This is a float approximation issue that is also commonly encountered when dealing with p-values. In the same way, it seems inefficient to distinguish highly significant relationships based on their logOdds, and we rely instead on the Log2FC as an effect size to rank these relationships. Defining an arbitrary and precise cutoff for *LogOdds* values also seems difficult and would depend on the user's appreciation of "highly significant" relations. Thus, we argue that replacing infinite values for *LogOdds* using an arbitrary and constant cutoff would not benefit the ranking that is already performed with Log2FC in these cases.

2.11.3 S3. Supplementary Figures

2.11.3.1 Figure S3 is low-resolution and difficult to read. Please include a higher-resolution version of this figure.

The figure has been updated in high-resolution.

2.11.3.2 The figures don't seem to match up with their references in the main text.

Mismatches between figures and references in the main text have been checked and corrected if needed.

2.11.3.3 All supplementary figures should be here (including S1 and all figures after S3).

We acknowledge the reviewer's suggestion to consolidate all the supplementary figures into a single section for better organization. However, almost all the supplementary figures (exception to Figures S2, S3, S4, S5) illustrate complementary analyses to evaluate the performances and behaviour of the method. Then, we believe that it would be beneficial to keep the majority of the supplementary figures within the flow of the different analyses. We recognize that this may come at the cost of better segmentation, but we feel it is necessary to facilitate the reader's reading and comprehension.

2.11.3.4 It is not clear what Figure S4C refers to in the main text. (now Supplementary Figure 5.C)

We apologize for this lack of clarity. The supplementary figure 5.C refers to the profile of the contributors when only 2 articles out of 33 would have mentioned the disease, which would have been sufficient to suggest this relationship with the proposed approach. We reformulate with the following sentences: “It is noteworthy that even fewer co-mentions would have already shifted the balance of contributors in favour of dopamine and highlighted this relationship. The figure S5.C shows the contributor profiles is the case where only 2 articles had mentioned the disease, which would have been sufficient to highlight the relationship.” We also added more details in the legend of the Figure.

2.11.4 S4.1 Damping factor α and theoretical sample size ν: benchmark. The validation dataset needs to be described before the results are presented. At this point, the reader has no idea what the validation set is.

Indeed, we thank the reviewer and moved this section on top of the supplementary materials.

2.11.5 S4.3 Evaluation using simulated overlooked metabolites

2.11.5.1 These results should be highlighted in the main text.

The sentence highlighting these results in the main text have been reworked: “To evaluate the performances of predictions based on the posterior distribution and the behaviour of the method on challenging cases, a supplementary analysis was conducted using simulated overlooked metabolites in Supplementary S4.4”. While the purpose of these analyses is to provide a more comprehensive evaluation of the proposed approach, we consider them to be secondary. Consequently, we prefer to redirect the interested readers to these sections for further information without elaborating on these observations in the body of the article.

☐ **2.11.5.2 "Focusing on overlooked metabolites, the most challenging scenarios are those where positive examples apparently show no co-mentions, and conversely, when co-mentions (e.g. anecdotal) wrongly support negative examples." Please highlight how you determined positive examples, negative examples, and co-mentions here.**

We thank the reviewer for pointing out this lack of clarity. The purpose of this analysis is to evaluate the performances of the approach on what we call “*Hard cases*”, which correspond to a subset of the simulated data in S4.4. Similarly to S4.1, positive examples are pairs of metabolites and disease-related MeSH extracted from the FORUM KG (q-value $\leq 1e-6$ with BH correction and no weakness), while negative examples are created by random combinations. For the purpose of simulating overlooked metabolites' data, the number of co-mentions (number of articles mentioning the both and supporting the relation) were randomly generated from a binomial distribution. We reworked the section S4.4 to clarify these potential ambiguities.

2.11.6 S4.4 Impact of the carbon skeleton graph on the predictions This should also be discussed in the main text.

We reworked the related paragraph in the main text to better highlight these results. Like the analysis on neglected metabolites, we consider this one as secondary and do not wish to detail the results in the main text.

Reviewer #3:

The authors present a tool (FORUM Literature Propagation) which is designed to help users query disease information relevant to a given metabolite by also querying a metabolic neighbors. This is accomplished by using a predefined network of metabolism (Human1) and querying PubChem for compound details and PubMed for articles containing disease and metabolite information. The authors have created a tool that is useful in finding potential associations of disease to metabolite, even when no articles have been published related to the specific metabolite in question This appears to be a useful tool for hypothesis generation, but should be used with caution as the results are inferred associations that may be skewed by regulatory mechanisms, the presence of highly studied metabolites, and highly 'promiscuous' metabolites which interact in a number of different pathways.

This review will focus primarily on the usability of the tool and the communication of that within the text. Summarily I find this to be a well written manuscript that does a good job of outlining the problem/need and appears to offer a solution. I do have some suggestions for clarifying the manuscript:

3.1 In the first paragraph of Method and Data Description the authors define a 'metabolic neighborhood' as "compound consists of the metabolites that can be reached through a sequence of biochemical reactions." Authors go on to reference the tools used to build and constrain the model. It would be additive to add some brief description to what was done prior, in addition to the more through explanation in supplemental information.

We thank the reviewer for helping us improve the clarity of the manuscript. We decided to add the following sentence in the corresponding section to better describe what is done with the atom-mapping procedure: “This results in a compound graph, built by linking two compounds when they share at least one carbon and have a substrate-product relationship in at least one reaction.”.

3.2 Continuing the above point this manuscript would be aided by a workflow diagram clearly illustrating the order of operations including key elements such as: user input, local database searching (Human1?), and PubMed/PubChem searching, result aggregation.

We thank the reviewer for this idea. Following this suggestion we added such a diagram. However, since the workflow encompasses various components, such as the extraction of FORUM associations and the conversion of the metabolic network into RDF, which are not the primary focus of our article, we have chosen to include the workflow diagram in the supplementary materials.

3.3 Figure 1 aids the reader to visualize FORUMs literature query process. However, it is a very dense figure that is difficult to extrapolate meaning from without carefully reading the Method and Data Description section. Ideally, this figure would be able to be understood by looking at the figure and its caption (current caption only details Blocks A and B). + Having blocks A-F and metabolites named A-F is also confusing, consider changing metabolites to numbers or Greek letters

We are thankful to the reviewer for pointing this out. We changed the figure annotation in order to make it more self-explaining. We decided to keep the capital letters for Figures' sub captions and renames the metabolites in lowercase. Some other details were also added to the figure according to different reviewers' comments.

3.4 What database is being used to define the metabolic network (pathways) and what identifiers are used to search those pathways for metabolic neighbors? Is this the pruned Human1 metabolic network and CIDs? More clarity here, would also be addressed by adding the workflow diagram suggested previously.

The metabolic network comes from a conversion of the Human1 SBML into RDF, and its content can be accessed from its own species identifiers or any referenced external identifier (CID, Chebi…) using the closeMatch property in SPARQL query. We added the workflow diagram to make this clearer, and, in addition to the data structure schema in the on-line documentation, we plan on adding pre-built example queries on the endpoint web page in the next release to make the search process more comprehensible.

3.5 Are the total number of metabolites available to use in this tool the 2704 mentioned in the Analysis section? Can this curated library be downloaded?

2704 is the total number of metabolites in the pruned version of the Human 1 v1.7 metabolic network. Among these metabolites, those with less than 100 annotated articles were considered as overlooked (2113 metabolites) and selected for analysis. Recall that this initial selection only serves as a prioritization and the method can be applied on the complete dataset. The library can be downloaded in a tabular file and in RDF format from the FTP server. Its content can be queried using the listed endpoint, from which the list of mentioned compounds can be retrieved in tabular format.

3.6 It appears to be a major limitation of this tool that over half of the 2704 metabolites do not have annotated PubChem CIDs, limiting the effectiveness of the tool in searching disease relevance.

Indeed, despite efficient cross-reference retrieval initiatives such as MetaNetX, many metabolites do not have annotated CID and thus can't be linked to any scientific literature. It is worth noting that the proposed method purposely alleviates such shortcoming by providing plausible associations for such compounds.
However, while some of those non-referenced compounds might not have any mentioning articles (or too few to confidently derive associations), some could have brought useful information and improved the associations regarding the former.
We could not find any means to know how non-referenced compounds are distributed among those two groups. However, we believe that most compounds without CID would

have yielded few or no articles, since we see a correlation between the number of mentioning articles and the prevalence of curated entries of such compounds in many databases, estimated by the number of retrieved cross-reference annotations.

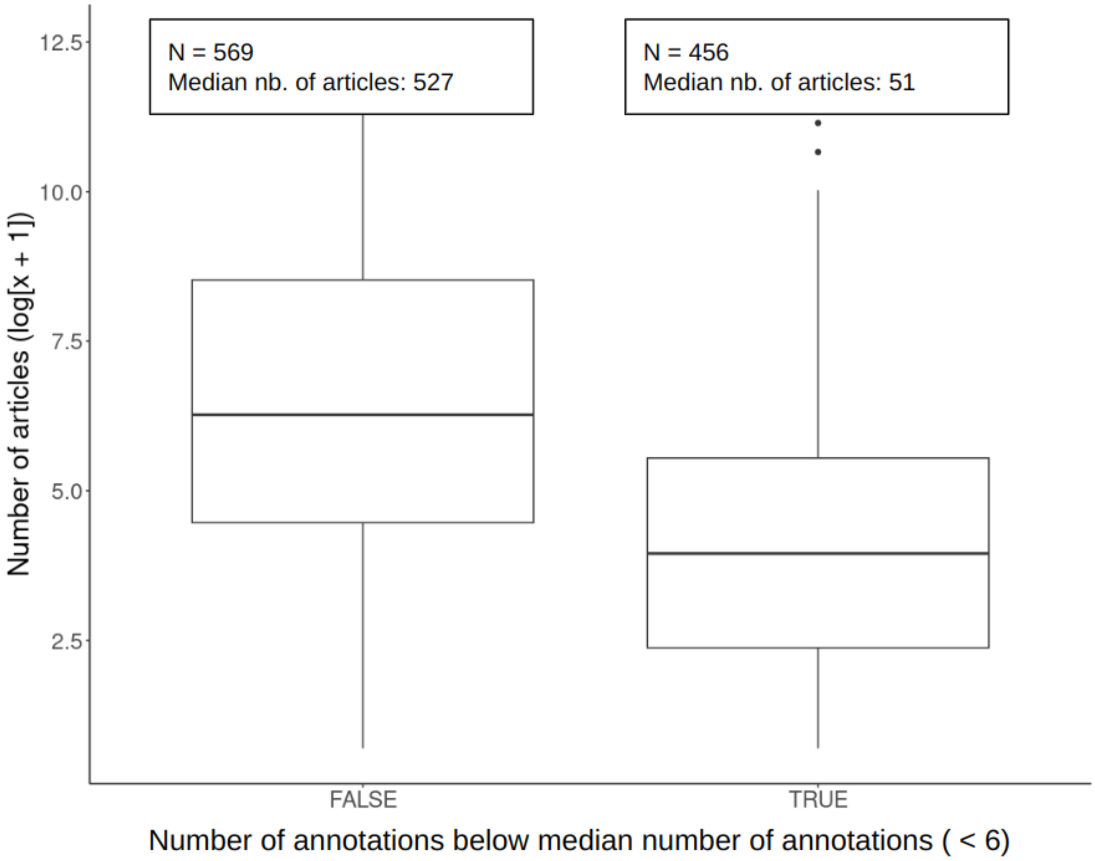

F4: Boxplot comparing the number of annotated articles among metabolic species in the network with more of fewer annotations than the median.

3.7 In the discussion section the authors simply state "many cannot be mapped to their corresponding PubChem identifier." Why? PubChem has over 100 million compounds, surely all the metabolites in the Human1 database have PubChem entries.

Some entries in metabolic models correspond to abstract entities rather than metabolites, such as “biomass” or “lipid pool”, which can explain a lack of PubChem reference. It is possible that other entries corresponding to metabolites could be found in the PubChem database, but the ambiguity and variability of chemical entity naming made such retrieval difficult and their mapping is still on hold, waiting for the ongoing collaborative refinement of Human1 and target databases to fix the issue.

3.8 Figure 2B has a typo in the caption. 16.5% should be 18.5% based on what is shown in the figure.

We thank the reviewers for noticing this typo. It has been corrected in the newer version.

3.9 Did the authors intend to say there are 1336 articles with PubChem identifiers in the figure 2 caption?

We are very grateful to this reviewer for spotting this error in the caption. Indeed, we meant 1336 compounds.

3.10 Figure 3 shows all 3 methods tested produced better AUC than Baseline-Freq showing the utility of metabolic neighborhoods however the graph gives this reader the impression that Baseline-DN and both α methods give very similar results. Perhaps a second panel of figure 3 could more articulately illustrate the difference in the methods as related to the neighbourhood parameter.

Figure 3 provides an overview of the evaluation of the built prior, a crucial component of our proposed approach. We assess the extent to which the literature in the metabolic neighbourhood of a compound contains relevant information about its biomedical context and can effectively guide predictions for rarely mentioned compounds. Baseline-DN serves as a strong baseline that shares similar assumptions with the approach when α = 0, but lacks the Bayesian component that incorporates an initial prior assuming independence (see "Mixing neighbouring literature to build a prior").
We established a direct connection between our approach and Baseline-DN by highlighting that when all direct neighbours have similar numbers of annotated articles and are not overlooked (with negligible shrinkage), the method with α = 0 behaves similarly to Baseline-DN. Then, close performances are expected. Furthermore, while the constructed prior is the sole source of information for predictions on metabolites without any annotated articles, predictions for metabolites with at least one annotated article rely on the posterior distribution. We assess this aspect by simulating overlooked metabolites in Supplementary S4.4, demonstrating the advantages of the Bayesian component in handling misleading observations.

We acknowledge that these results were not sufficiently emphasized in the main text, and we have reworked this paragraph to address the suggestions, also considering the comments raised by reviewer 2.

3.11 Figure 4 and 5 it is not clear if there are any differences in the Contributor Odds based on the color scaling almost all sections appear the same shade of red.

Figures 4 and 5 exhibit instances where the neighbourhood strongly suggests a relationship between the targeted metabolite and the disease. In these cases, the individual *logOdds* values for all contributors are high (>~ 50), resulting in almost the same colour based on the applied scale. The range of possible *logOdds* values is large ([< -100, > 100]) and we acknowledge that small differences are difficult to appreciate. However, considering the logarithmic scale used for *logOdds*, the primary purpose of this colour scale is to quickly identify negative, neutral, or positive contributions and the transitions between these states, such as in Supplementary Figures S3, S5, and S13. Additionally, we have provided the complete list of *logOdds* values in the supplementary tables referenced in the corresponding

figures' legend. All other examples of figures can be browsed here: https://forum-static-files.semantic-metabolomics.fr

3.12 How are Specie IDs assigned? It is not an identifier I have used. Can InChiKey, SMILES, CID, HMBD IDs, be readily converted to Specie IDs? If so, how?

Species IDs are manually or automatically assigned during reconstruction, following conventions that vary between models. Nonetheless, each species is manually curated to be annotated with external identifiers such as CID or CHEBI to ensure interoperability. Databases such as MetaNetX gather all cross-references between such external databases and identifier systems such as InChIKey or SMILES, and thus enable conversions. In practice, one can directly access a compound from a metabolic network in FORUM using its ID in query, or alternatively access it by querying a compound with an annotation that match directly or indirectly a given identifier under any system present in the MetaNetX dataset.

3.13 Is there a mechanism in place for limiting the scope of the query? For instance, if I am studying *denovo* purine synthesis and quantifying metabolites from the pentose phosphate pathway is there a way to exclude contributions of glucose-6-phosphate as it would surely skew my results towards glycolysis intermediates.

We appreciate the reviewer's insightful question. Indeed, it is possible for highly influential compounds to dominate the suggestions and overshadow the contributions of other contributors (as discussed in the Limitation section, using ethanol as an example). In such cases, it may be desirable to adjust the weight vectors to either suppress certain compounds. One can directly modify the weight matrix that contains the $\mathbf{w}_{i,k}$ values for all possible pairs of metabolites. Then, the influence neighbourhood of each metabolite is entirely customizable and is not limited by what may be imposed by the structure of the network. These weight matrix matrices are stored in a cache directory created by the method in the working directory, allowing for direct edits of the weight values.

3.14 Can the authors elaborate on why Human1 was chosen over larger metabolic pathway libraries (KEGG, SMPDB, Biocyc, Reactome)?

One aspect of such choice is the licensing of the data, some, despite being freely accessible, forbid bulk download or restrict their usage to a scope that is not compatible with the proposed work under FAIR principles.
Another aspect is related to the curation of links. As this work relies on guilt by association principle, the quality of the relationship used is of utmost importance to avoid propagation of irrelevant information. Human1/HumanGEM is not a data repository but a functional metabolic model. As such, simulations guarantee agreement between the data and expected behaviors and allows for another degree of refinement, often correcting small inconsistencies that could be found in larger generic databases.
