# Supplementary material for: Suggesting disease associations for overlooked metabolites using literature from metabolic neighbors
Source: Gigascience. 2023 Sep 15;12:giad065. doi: 10.1093/gigascience/giad065 (PMC10502579; doi:10.1093/gigascience/giad065)
Supplement: giad065_Response_to_Reviewer_Comments_Original_Submission [file giad065_response_to_reviewer_comments_original_submission.pdf]

Author's Response To Reviewer Comments

Response to Reviewers

Reviewer #1:
Manuscript Number: GIGA-D-23-00014, entitled, " Suggesting disease associations for overlooked metabolites using literature from metabolic neighbours", and submitted to the journal: GigaScience, applied 'guilt by association' principle to literature information for "understudied metabolites" by using a Bayesian framework. It is an interesting manuscript, an active area of research and would have an interest in the metabolomics research community. However, this reviewer would like to help improve the manuscript and scope of the work with the following suggestions:

1.1 A list/ DB of all such "overlooked metabolites" and their chemical class distribution/ ChemRICH sort of enrichment would help the readers capture the correct information.

The authors thank the reviewer for bringing this suggestion. The complete list of overlooked metabolites (2113 species) have been added on the GitHub repository and in the FTP server. Since overlooked metabolites can have limited annotations in standard chemical ontologies such as Chebi or MeSH, we decided to use ClassyFire. ClassyFire provides an automatic hierarchical classification of molecules based on structural descriptors such as inchiKey identifiers. We managed to obtain an InchiKey for 1180 (approximately 56%) out of the total 2113 metabolites considered as overlooked in the metabolic network using their annotation in MetaNetX. Subsequently, we analyzed the distribution of the superclass to which these metabolites are classified by ClassyFire. SuperClasses are generic categories of compounds that we can use to get an estimation of the composition of chemical families in the set of overlooked metabolites for this metabolic network. From this sample, it can be estimated that the majority of metabolites considered as overlooked in this metabolic network are actually "Lipids and lipid-like molecules" (e.g: Fatty Acyls, Sphingolipids, etc.), a class with a strong compositional complexity. However, this observation is based on a limited sampling of metabolites within a specific metabolic network. As a result, this subset is unlikely to be representative and while it could give some insights, we argue that it could lead to misleading interpretations and decided to not add this directly in the article.

A figure of the distribution of the chemical superclass obtained with ClassyFire can be found in the attached document.

1.2 How did/ would the tool perform with "very well known metabolites" for example say, phenylalanine or proline or citric acid ?

We thank the reviewer for this interesting remark. The behaviour of the method for “very well known metabolites”, as opposed to overlooked metabolites, is quite straightforward in the Bayesian settings. The impact of the prior on the predictions will vanish as the literature of the targeted compound increases. The LogOdds estimator then tends to infinity, while Log2FC tends to its exact value when estimated only from the literature of the compound. For instance, among the 278,277 articles discussing the glucose in FORUM, 24.839 co-mentioned the Diabetes type 2 MeSH descriptor. The prior and posterior distributions obtained for this relationship are presented below. The posterior distribution is solely driven by the literature of the glucose which, being much larger than that of its contributors, completely erases the information brought by the prior. The distributions of the contributors in the posterior mixture are therefore centred around the co-mention frequency of the glucose and Diabetes type 2 (≈ 0.089). Thus, although the proposed approach can be applied to these well-known metabolites, the predictions are insensitive to the built prior which is nevertheless at the core of this method. In this case, the relationships would be as well evaluated with a classic over-representation analysis.

In addition to the aforementioned extreme example, a similar phenomenon can be observed through an example proposed by the reviewer: Phenylalanine (specie id M_m02724c) and the MeSH descriptor

Phenylketonurias or PKU (D010661). PKU represents a group of disorders caused by a deficiency in the production of phenylalanine hydroxylase, and for which the dosage of phenylalanine is the standard diagnostic method. Again, the posterior distribution eliminates any information from the prior and is centred around 0.0107, which is the expected probability that an article mentioning phenylalanine also mentions the disease. Indeed, out of the 28.507 articles mentioning Phenylalanine, 3.045 are annotated with the MeSH term PKU.

Figures of the Prior and posterior distributions of this two examples can be found in the attached document.

1.3 How does one check for "literature / reporting biases" for the highly reported vs lowly reported metabolites in the manuscripts ?

From our understanding, hoping we interpret correctly reviewer comment, this check would be related to the retrieval of metabolites' mentioning articles. We hope that the following information can answer your question:
There are several ways one can access the literature of metabolites described in this manuscript. First, all the data are publicly available in the git repository https://github.com/eMetaboHUB/Forum-LiteraturePropagation where
uncompress_species_pmids_Human1_1.7.csv contains the number of annotated articles for each of the 2704 species in the pruned version of Human1 metabolic network. If one desires to recover the list of PubMed identifiers behind these frequency values, the FORUM KG (https://forum-webapp.semantic-metabolomics.fr) is the most direct way of recovering the original set of articles mentioning a metabolite. However, as this extraction requires querying the SPARQL endpoint, which we acknowledge is difficult for non-familiar users, we would recommend accessing it individually for each compound from their PubChem page or directly on PubMed.

1.4 Does this approach distinguish for targeted vs untargeted metabolomics paper based hits ?

We thank the reviewer for this interesting remark. Although we could increase the confidence of hits from targeted analyses compared to untargeted using some weighting policies (or using Metabolomics Standard Initiative classification for metabolite identification), the main challenge would lie in accurately extracting this information. In fact, articles related to metabolomics analyses are not yet indexed in PubMed with a precise MeSH term to distinguish the two types of approaches. Determining this from the title or abstract would also require building a classification model for which training data are not available. More generally, proposing a different weighting for the contribution of each article according to different factors (type of analysis, date, etc.), so that they are not all considered equivalently, is indeed an interesting perspective for future works.

1.5 "Overlooked metabolites" need to be defined well, upfront for clarity.

We are grateful to the reviewer for pointing out this lack of clarity. We propose to modify the end of the first paragraph of the Method section: "In this study we define a set of overlooked compounds as compounds with less than 100 retrieved mentioning article, which correspond to orders of magnitude below 4,799, the mean number of retrieved articles per compound (when any), and is close to the median number of articles, 172. It is worth mentioning that such threshold serves solely as a prioritization criterion, since the method applicability is not restricted to a given range of mentioning corpus sizes (although its relevance is less obvious when a sufficient corpus is already available)."

1.6 large fraction of metabolites are rarely or never mentioned in the literature: What is a good estimate from the authors? A numerical value would be informative here.

While our results from the metabolic network clearly suggest that a large fraction of metabolites are

overlooked, we argue that this information, although reflecting a reality in the field, cannot be used to propose a reliable estimate. This estimator would be biased by various factors and in the first place, the lack of external identifiers (e.g. CID) that connect to the literature. Additionally, the purpose of the metabolic network is not to provide an exhaustive map of the metabolism and some parts (e.g lipid metabolism are often reduced to generic classes). Nonetheless, our estimate based on the whole PubChem database seems more reliable and we decide to put the emphasis on it in the abstract to provide a numerical value. We therefore reworked the abstract by adding the following sentence: “However, we show that the vast majority of compounds (> 99\%) in the PubChem database lack annotated literature. This dearth of available information can have a direct impact on the interpretation of metabolic signatures, which is often restricted to a subset of significant metabolites}.”

1.7 Too many terms used does not help: overlooked metabolites vs. understudied metabolites and so on. Please use a singular term for consistency.

We thank the reviewer for helping us improve the readability of the manuscript. We replaced every mention of “understudied” with “overlooked”.

1.8 Method and data description section is too wordy, need to be shortened and need to use mathematical expressions whenever applicable.

We appreciate the feedback regarding the "Method and data description" section of our article and we acknowledge that this section may be too wordy and lacking in mathematical expressions. We made some improvements and tried as much as possible to reduce the size of this section.
We made this choice given the potential readership of the work. We anticipate that some readers wishing to use the provided associations to interpret their results, may not have a strong mathematical background. Therefore, while the use of mathematical expressions would shorten the section, it could also be a barrier to its understanding and discourage some readers. We have strived to make our methodology as accessible as possible by providing two descriptions, which we believe will complement each other. Our primary focus in the "Method and Data description" section is to provide an intuitive and concise overview of the main steps of our approach, avoiding the use of mathematical expressions.
Simple expressions have been added to this section according to the various reviewers' comments in order to remove potential ambiguities. In addition, a complete description with all the mathematical details is provided at the end of the manuscript in the method section for the interested readers.

Reviewer #2:
Overall Notes
This work is innovative and will provide an important contribution to the computational metabolomics field. The experiments and methodology are well-designed and executed, and the software is also well-documented. That being said, the structure and writing of the manuscript needs to be reworked. There are several areas of the text where descriptions are unclear, detailed below. Some of the text is also out of order, e.g. weights are shown in a figure before they are defined, and TPR and FPR are reported without describing the dataset. Finally, there are several Supplementary experiments that are never mentioned in the main text. At least a brief description of these should be given in the main text and then the Supplementary referenced.

2.1 Abstract
Some of the language used here is difficult to read or unclear. In particular:
2.1.1 I believe you mean to say that signatures… "have a strong added value", not "are a strong-added

value".

We corrected this in the manuscript.

2.1.2 "we extend the 'guilt by association' principle to literature information by using a Bayesian framework". This is vague. Instead, briefly explain how you use a Bayesian framework to determine guilt by association.

We reworked the abstract and specifically added the following sentence to briefly illustrate the intuition behind the prior and the Bayesian framework in the context of the guilt by association principle: “The underlying assumption is that the literature associated with the metabolic neighbours of a compound can provide valuable insights, or an a priori, into its biomedical context.”

2.1.3 "1,047 overlooked metabolites". Do you mean metabolites not in the literature?

Not exactly, we meant metabolites which are rarely mentioned in articles (< 100 annotated articles), so they almost never mentioned in the literature. As this notion of “overlooked” metabolites is key in this article, it has also been clarified in section “Method and data description” according to the Reviewer 1 comments.

2.1.4 Your method uses knowledge about metabolic interactions/reactions to generate the graph, but this is not mentioned at all in the abstract. The abstract should explain that this knowledge is being used and describe how it is complementary to the literature.

Following the previous comments and the addition of the underlying hypothesis in the abstract, we also decided to add the following sentence to emphasize the role of the metabolic network in defining the structure of the graph used to propagate information: “The metabolic neighbourhood of a compound can be defined from a metabolic network and correspond to metabolites to which it is connected through biochemical reactions.”

2.2 Background
2.2.1 it is irrelevant to mention exponential growth. This detracts from the main point, which is the imbalanced knowledge distribution.

We removed this part of the sentence from the manuscript.

2.2.2 "This topic has received much attention for genes and proteins…" Can you provide some citations?

The references related to this statement were provided in the next sentence (“Consequently, [...] gene annotations in databases”). According to this reviewer comment, we decided to move them upstream.

2.2.3 “has an impact on the quantity and quality of gene annotations in databases" - in what way? Can you be more specific?

We wanted to highlight that the skewed distribution of the number of bibliographic references across genes is also reflected in the distribution of functional annotations in databases, such as Gene Ontology. See for instance between TP53 and ANKRD52. As it doesn’t bring much more details for the rest of the article and could distract the reader, we decided to remove this sentence.

2.2.4 The first sentence of the second paragraph can be removed.

This sentence has been removed according to the reviewer's suggestion.

2.2.5 You discuss the issue of inaccurate identification as being related to the number of articles mentioning a compound. I feel that these are two separate issues. The first is related to identification, and the second is related to discussion of identified metabolites in the literature. The section regarding identification should be removed.

This section has been removed according to the reviewer's suggestion.

2.2.6 "Guilt by association principle", not hypothesis.

This has been corrected.

2.2.7 "The method returns several predictors to evaluate whether a significant proportion of the articles mentioning a metabolite would also mention a disease." Do you mean to say that the predictors predict whether or not the metabolite is related to the disease?

Indeed, by indicating whether a significant proportion of the articles mentioning a metabolite would also mention a disease, these predictors are meant to highlight a potential relation. We acknowledge that this could be expressed more explicitly in the background section, leaving this interpretation for the methodology section. We reworked this sentence accordingly.

2.2.8 You mention that you used FORUM Knowledge Graph to obtain your metabolite-disease associations. What about the metabolic neighborhoods? You should explain where these were obtained.

The metabolic neighbourhoods are defined from the Human 1 (v1.7) metabolic network, which was also pruned from spurious connections using an atom-mapping procedure. While we keep the details apart from the main text, we reworked the following sentence: “Metabolic neighbourhoods were defined from the Human1 metabolic network and co-mention data between metabolites and diseases were extracted from the FORUM Knowledge Graph (KG)”.
The details of the pre-processing step on the metabolic network and its implication of the results are detailed in Supplementary materials (S1.1, S4.5) and referenced in Method and Data Description.

2.3 Method and Data Description
2.3.1 How do you define "rarely mentioned"? Is there a cutoff criteria used?

We thank the reviewer for this interesting remark, which has also been highlighted by the other reviewers. We believe that the modifications applied to the first paragraph of the method section should clarify what we meant by "overlooked" or "rarely" mentioned metabolites, both conceptually and practically.

2.3.2 Does "amount of literature" mean number of articles?

Yes, we reformulated the formulation "amount of literature" everywhere in the article to bring clarity, as suggested by this reviewer.

2.3.3 How is "far distant" defined? It seems that you mean to say that one metabolite's influence on another decreases as the number of reactions separating them increases. Is this correct?
We thank the reviewer for pointing out this lack of clarity. Indeed, we make the assumption that the influence of a metabolite on another decreases as the number of reactions separating them increases. In

order to consider all the potential paths connecting two metabolites in the network, we use the stationary probabilities from random walks starting from the former and reaching the latter as a measure of this distance.
However, when we referred to “far distant” metabolites in the sentence: “We impose that a metabolite can't influence its own prior or the prior of far distant metabolites.”, we inaccurately referred to metabolites whose probability of being reached during the random walk are below a predefined threshold. This concerns, for instance, metabolites that belong to different regions of the metabolic network. This constraint prevents influential metabolites (tryptophan, glucose, etc.) from sharing the articles mentioning them with metabolites that are unlikely to be involved in the regulation of common metabolic pathways.

Since this detail is explained thoroughly with mathematical expressions in section "Estimating the contributions of metabolic neighbours" in Method for interested readers and is not crucial for the understanding of the approach as a whole, we have chosen to exclude it from the method summary. The Figure 1 has also been updated accordingly.

2.3.4 Show Figure 1 as soon as it is mentioned. This goes for the other figures as well.

Indeed, the initial position of the figure was not ideal to follow the corresponding method in the main text, so we moved it one page closer.

2.3.5 You should explain more about the shrinkage procedure here. It isn't clear what you mean.

We are thankful to the reviewer for helping us to make this paper more clearer, particularly for the shrinkage step which is a key element in the presented approach. While the idea of shrinkage is also used for penalized regression, in this manuscript we refer to its applications in Bayesian settings. In this framework, the posterior mean distribution is shrunk towards the prior mean (μ), resulting in a more reliable estimator than the maximum likelihood estimator (MLE) for low sample sizes. This is illustrated in equations 5a and 5b when we show the posterior distribution of pi. The parameterization of the prior beta distribution involves determining μ, assuming that metabolites and diseases are independent concepts in the literature, and setting the sample size (v) as a hyperparameter to control the strength of the prior.
We decided to modify the corresponding paragraph in the method summary section according to the reviewer’s comments : “This results in a small sample size available to estimate the probability that an article mentioning f also mention the disease, which may lead to unreliable and spurious contributions. To address this, a shrinkage procedure is applied to all contributors, assuming that a priori, mentioning a metabolite in an article does not affect the probability of mentioning a particular disease. In Bayesian settings, a shrinkage estimator integrates information from the prior to readjusted raw estimates, reducing the effect of sampling variations (further details in section Mixing neighbouring literature to build a prior in Methods).“
We also redirect the interested reader to the Method section for the mathematical details.

2.3.6 In Figure 1C, there appears to be a stack of papers in a pink box in both the numerator and the denominator. What does this mean?

We thank the reviewer for pointing out this unclear illustration. This pink box represents the number of papers shared by b that reached the target compound a. This quantity is noted tb,a. In Figure 1.C, we illustrated the simple computation of the weight of b in the prior of a (noted wb,a) as the fraction of articles that reached A (tb,a + tc,a + te,a + tf,a) that was send by b (tb,a). To avoid any other ambiguities in this illustration, we explicitly annotate all the paper box with their corresponding value (e.g; tc,a or wb,a) both in 1.B and 1.C.

2.3.7 "Then, we build the prior distribution for A, by mixing the probability distributions of each contributor (see Figure 1.E) according to their weights estimated in the previous step (Figure 1.C)". This is the first time you mention weights. You need to describe what the weights correspond to and how they are

calculated first.

We apologize for adding this ambiguity. We reworked this section, both at the first mention of the weights and in the highlighted sentence. We also added a more explicit mention of the weights associated with the contributors using simple mathematical expressions: “We refer to b, c, e and f as the contributors to the prior of a. Each contributor has a weight w in the prior of a (e.g wb,a) proportional to its contribution.” We also add a reminder in the following sentence: “Then the prior distribution of a is built as a mixture of the probability distributions of individual contributors (b, c, e and f) as illustrated in Figure 1.E. Recall that the weight of each contributor in the mixture is (w.,a), as estimated in the previous step (see Figure 1.C).”

2.3.8 "Then, we build the prior distribution for A, by mixing the probability distributions of each contributor (see Figure 1.E) according to their weights estimated in the previous step (Figure 1.C)". This sentence is unclear. Please revise.

We reformulate this sentence according to the previous comment. Please, see the previous answer.

2.3.9 "Finally, several diagnostic values such as Entropy allow to assess the composition of the built prior (See Supplementary S1.3)". Either name all of the diagnostic values here or move Entropy to the supplementary and out of the main text.

As suggested by the reviewer, we removed the mention of Entropy in this sentence.

2.3.10 "Entropy evaluates the good balance of contributions in the prior. The more metabolites contribute to the mixture and the more their weights are uniformly distributed, the higher the entropy." This is not a clear explanation. Please explain mathematically what entropy is and what it represents here.

According to the last two comments of this reviewer, we reworked this paragraph. We decided to focus more on the applications and purposes of these diagnostic indicators rather than their formal definitions, which we decided to keep for the supplementary materials for the interested readers. We therefore replace the sentence relative to Entropy to a broader description of the purposes of the diagnostic indicators: “Finally, given its primary role in driving predictions, assessing the composition of the constructed prior is crucial. Essentially, the more contributors to the prior, close to the target compound, with balanced weights, the better it captures the neighbourhood literature and increases the confidence in predictions. To aid in this evaluation, a set of diagnostic indicators is presented in Supplementary S1.3‟.

However, as Entropy is the only diagnostic indicator used in the main text (for filtering the predictions), we also added a more formal definition directly where it is mentioned in section “Suggesting relations with diseases for overlooked metabolites”. See comment 2.6.3.

2.4 Analyses: Unbalanced distribution of the literature related to chemical compounds
2.4.1 At the end of the first paragraph, it should be "is cumulatively less than the literature associated with glucose…"

This has been added to the manuscript.

2.4.2 Can metabolites without a CID be found in the metabolic network? If not, then you should not discuss those metabolites with an unannotated CID.

With the exception of the pruning process carried out during the construction of the carbon skeleton graph and described in S1.1, no metabolites were excluded because of a lack of annotations. All metabolites without an annotated CID are conserved in the metabolic network.

2.5 Analyses: Evaluation of the prior computation
2.5.1 "and is set to ɑ = 0 for the direct neighbourhood and ɑ = 0.4 for a larger one". Are these the only two values you consider? If so, why? How large is "larger"? This should also go in the methods section.

We thank the reviewer for pointing out this lack of clarity. An extensive analysis was actually performed on the impact of the both parameters ɑ and ν on the performances and the composition of the prior in S4.3 Damping factor ɑ and theoretical sample size ν: benchmark. We evaluated values for ɑ in the set: [0, 0.1, 0.2, 0.3, 0.4, 0.5, 0.6, 0.7, 0.8, 0.9, 0.99] and for ν in the set [1, 10, 100, 1000, 10000, 100000 , 1000000]. From the performed analyses, we found that ɑ=0.4 and ν=1000 appears to be reasonable setting, both in terms of composition of the built prior and balance between sensibility and precision. Finally, we also argued that there are no global optimal settings and recommend 0 ɑ 0.7 and 1 ν 10000. We reworked this paragraph to point to these details in the supplementary materials when referring to the chosen values of ɑ: “We therefore focused on two specific settings: ɑ=0, where solely the direct neighbours contribute to the prior, and ɑ=0.4, where contributions between direct or indirect neighbours are relatively balanced. The impact of the parameter ɑ on the construction of the prior and the Precision-Recall tradeoff was extensively evaluated in Supplementary Material S4.3.”

2.5.2 "All tested approaches outperform Baseline-Freq, showing the benefit of examining the neighbouring literature." This experiment needs to be explained in the main text. How did you define TPR and FPR?

Indeed, the evaluation results with the ROC curves were not explicitly presented in the main text and we decided to rework this paragraph to add “The evaluation results on the validation dataset for all described approaches are presented in Figure 3.”. We also add a description of TPR and FPR in the legend of Figure 3: “A true positive represents an association between a compound and a MeSH term which is both retrieved from the compound's mentioning corpus using Fisher Exact Test, and using methods in which no knowledge of such corpus is available. A false positive is only retrieved from the latter.”

2.6 Analyses: Suggesting relations with diseases for overlooked metabolites
2.6.1 "However, by re-evaluating these predictions using a right-tailed Fisher exact Test (BH correction and selecting those with q.value <= 0.05), we found that ≈ 50% of them (925) would not have been found significant". Can you please explain this experiment further?

We thank the reviewer for pointing out this lack of clarity. With this analysis we wanted to emphasize the proportion of associations that would not have been highlighted by a standard approach, thus without considering the neighbourhood information. The right-tailed fisher exact test is a standard and robust approach for over-representation analysis of associations in the literature that we already applied at large scale in the first version of FORUM. We therefore used this same workflow to test all associations between metabolites and diseases based on their co-mention frequency from the literature available in the metabolic network. We reformulate this paragraph and directly refer to the analysis done in the FORUM article: “However, by re-evaluating them using the same workflow as in FORUM [FORUM 2021] (a standard over-representation analysis (ORA) using right-tailed Fisher exact Test, BH correction and threshold on q.value <= 0.05), we found that ~50% (925) of these associations would not have been highlighted.”

2.6.2 "These relations are still weakly supported, nevertheless, our method showed that they are consistent with the neighbourhood." What does this mean?

We apologize for this lack of clarity. Despite these relationships being supported by only a few articles, the proposed approach showed that these are consistent with the literature of metabolic neighbours. These limited mentions could therefore be significant and deserve consideration. By using a standard ORA for comparison purposes, we also wanted to emphasize that the current volume of articles supporting a relation in the literature may simply not be sufficient (quantitatively) to effectively highlight it with this type of approach. We reworked the sentence as follows: “While only a few articles support these relationships and half of them were discarded by a standard ORA, the method showed their consistency

with the literature of metabolic neighbours”.

2.6.3 Why do you want high entropy in Table 1? This isn't clear if the reader doesn't know what entropy refers to here.

As we removed the definition of Entropy in the method summary (see comment 2.3.9 and 2.3.10), we reworked this paragraph and added both an introduction oriented on the application of this indicator and a definition that we hope clearer. We replaced the paragraph “We also retained predictions based on well-balanced contributions from the neighbourhood by filtering on the diagnostic indicator Entropy > 1 (See details in Method and Supplementary S1.3).”, by: “Predictions for which the prior was biased toward one dominant contributor and thus failed to capture the neighbourhood literature, were excluded by filtering on the diagnostic indicator Entropy > 1. Entropy is the Shanon entropy computed on the contributors weights in the prior: the more contributors with balanced weights, the higher the entropy. (See details in Method and Supplementary S1.3).” We again direct the reader to the Supplementary materials.

2.7 Limitations

2.7.1 "Although we kept it in our analysis for sake of exhaustively…" It is not clear what this means.

We apologize for the lack of clarity. We wanted to emphasize that despite influential compounds like ethanol could provide out-of-context relations, we did not apply a filter to try to exclude them. We reworked this sentence: “To avoid arbitrary filtering, we left to the
user the choice to keep associations with such compounds after review”.

2.7.2 Methods Your equations are not numbered correctly. Your equation (1) should be equation (2). It looks like you have 14 equations total, but only one is numbered.

All the equations have been numbered in the new version of the manuscript.

2.7.3 Settings
2.7.3.1 How did you choose the cutoff in equation (1)?

As the probability to be reached by a random walk depends on the shortest distances within a network, we define a threshold that scales with the size of the network. We set the threshold to 1/(n - 1), which is the probability that a metabolite would be randomly chosen among all potentially reachable metabolites. It is important to note that this threshold is a default value and can be changed when calling the script to compute the associations (option -q: The tolerance threshold).

2.7.3.2 "These aspects are illustrated in Figure 1.B: B…" This entire paragraph should be moved up to the Method and Data Description section.

The paragraph has been reworked and an equivalent description is provided in section “Methods and Data description”. Nevertheless, we believe that even in this more technical description of the method, providing a link with the illustration in Figure 1.B may help to capture the behaviour of the method.

2.8 Mixing neighbouring literature to build a prior
2.8.1 Explain what the Beta distribution parameters mean in the context of this study in detail and why you chose a Beta distribution.

Being defined in the interval [0,1], the Beta distribution is a suitable model for modelling proportion, which is precisely what we want to estimate: the proportion (or probability) of articles mentioning a metabolite,

that also mention a disease. Secondly, the beta distribution is the conjugate prior of the Binomial distribution, modelling the number of observed successes in a sequence of n trials, which is also the type of data that we have: among ni articles mentioning a metabolite, yi (successes) mention a disease. From this, the Beta-binomial model appears as a suitable framework for the purpose of this work.

When building the prior, the essential assumption is that a priori a metabolite and a disease are independent concepts in the literature. For all metabolites in the network, we start by modelling their prior probability of mentioning the disease with a Beta distribution parameterized under this hypothesis: the average probability equals the overall probability P of mentioning the disease in an article. The Beta distribution is therefore parameterized by mean μ and sample size ν, which determine the values of the two shape parameters α and β. μ being fixed to P, ν is actually the only hyperparameter setting the initial prior. Also, since μ is set to P, this default prior would not suggest a relation using LogOdds or Log2FC. Additionally, ν is related to the amount of evidences in the literature needed to change this prior belief. Thus, one should not directly interpret the values of α and β, as the real fixed parameters are μ and ν. More explanations have been added to the method section regarding the Beta distribution and the implication of the parameters.

2.9 Updating prior and selecting novel associations
2.9.1 "In turn, Log2FC is much more sensitive to outlier contributors than LogOdds". Please provide a citation for this.

This is a common problem with outliers when an estimator is based on a mean, we reworded this sentence to highlight this and provided a reference for this statement in the manuscript.

2.10 References
2.10.1 Check the formatting for #27. It is overlapping the text in the right-hand column.

This has been fixed.

2.10.2 If you're going to discuss the Pareto Principle (28), try to find a review article that describes this principle rather than referencing a textbook.

This reference comes from a collection of commissioned introductory review articles and is highly cited; we believe it is appropriate in this context, and sufficient to grasp the concept needed to understand this section.

2.11 Supplementary Material
2.11.1 S1.3 Diagnostic Values

Here, it is clear what you mean by Entropy and why you want the value to be high rather than low. This should go in the main text. However, it is concerning that the meaning of the entropy cutoff changes with respect to the number of contributors. Consider using a weighted metric that has the same meaning regardless of the number of contributors.

We apologize for any confusion caused by the provided numerical examples. The meaning of Entropy remains the same regardless of the number of contributors. By considering a weighted metric that is normalized by the number of contributors, the reviewer may be referring to normalized entropy, also known as Efficiency, which is the observed Entropy divided by the maximal entropy (log2(N)).
However, imposing a fixed proportion of maximum entropy regardless of the number of contributors is not reasonable. For example, reaching 50% of the maximal entropy is easier when there are 5 contributors than when there are 50. Therefore, our choice aims to maintain a balanced distribution of contributors, becoming more flexible as the number of contributors increases. To address this, we set the threshold for Entropy at 1. This means that when there are only 2 contributors, the maximum entropy is required, and

as the number of contributors increases, we progressively relax this constraint on a logarithmic scale. For example, with 5 contributors 50% of the maximum entropy is required, for 10 contributors 30%, for 30 contributors 20%, for 100 contributors 15%, etc. We reformulated the corresponding paragraph in the supplementary materials. Finally, the selected threshold is a recommendation for the specific analysis we conducted where we aimed for stringency and users are free to set their own threshold according to their needs.

2.11.2 S2. Supplementary Tables
Why are your LogOdds values infinite? If this is an issue with taking the log of 0, then you should set a cutoff such that the values do not go to infinity.

The posterior error that an article mentioning the metabolite k, would mention the disease more frequently than expected is noted CDF and corresponds to P(pk < P). As CDF tends to 0 for strong relationships, logOdds will logically tend to infinity. This is a float approximation issue that is also commonly encountered when dealing with p-values. In the same way, it seems inefficient to distinguish highly significant relationships based on their logOdds, and we rely instead on the Log2FC as an effect size to rank these relationships. Defining an arbitrary and precise cutoff for LogOdds values also seems difficult and would depend on the user's appreciation of "highly significant" relations. Thus, we argue that replacing infinite values for LogOdds using an arbitrary and constant cutoff would not benefit the ranking that is already performed with Log2FC in these cases.

2.11.3 S3. Supplementary Figures
2.11.3.1 Figure S3 is low-resolution and difficult to read. Please include a higher-resolution version of this figure.

The figure has been updated in high-resolution.

2.11.3.2 The figures don't seem to match up with their references in the main text.
Mismatches between figures and references in the main text have been checked and corrected if needed.

2.11.3.3 All supplementary figures should be here (including S1 and all figures after S3).

We acknowledge the reviewer's suggestion to consolidate all the supplementary figures into a single section for better organization. However, almost all the supplementary figures (exception to Figures S2, S3, S4, S5) illustrate complementary analyses to evaluate the performances and behaviour of the method. Then, we believe that it would be beneficial to keep the majority of the supplementary figures within the flow of the different analyses. We recognize that this may come at the cost of better segmentation, but we feel it is necessary to facilitate the reader's reading and comprehension.

2.11.3.4 It is not clear what Figure S4C refers to in the main text. (now Supplementary Figure 5.C)

We apologize for this lack of clarity. The supplementary figure 5.C refers to the profile of the contributors when only 2 articles out of 33 would have mentioned the disease, which would have been sufficient to suggest this relationship with the proposed approach. We reformulate with the following sentences: "It is noteworthy that even fewer co-mentions would have already shifted the balance of contributors in favour of dopamine and highlighted this relationship. The figure S5.C shows the contributor profiles is the case where only 2 articles had mentioned the disease, which would have been sufficient to highlight the relationship." We also added more details in the legend of the Figure.

2.11.4 S4.1 Damping factor α and theoretical sample size ν: benchmark. The validation dataset needs to be described before the results are presented. At this point, the reader has no idea what the validation set

is.

Indeed, we thank the reviewer and moved this section on top of the supplementary materials.

2.11.5 S4.3 Evaluation using simulated overlooked metabolites
2.11.5.1 These results should be highlighted in the main text.

The sentence highlighting these results in the main text have been reworked: “To evaluate the performances of predictions based on the posterior distribution and the behaviour of the method on challenging cases, a supplementary analysis was conducted using simulated overlooked metabolites in Supplementary S4.4”. While the purpose of these analyses is to provide a more comprehensive evaluation of the proposed approach, we consider them to be secondary. Consequently, we prefer to redirect the interested readers to these sections for further information without elaborating on these observations in the body of the article.

2.11.5.2 "Focusing on overlooked metabolites, the most challenging scenarios are those where positive examples apparently show no co-mentions, and conversely, when co-mentions (e.g. anecdotal) wrongly support negative examples." Please highlight how you determined positive examples, negative examples, and co-mentions here.

We thank the reviewer for pointing out this lack of clarity. The purpose of this analysis is to evaluate the performances of the approach on what we call “Hard cases”, which correspond to a subset of the simulated data in S4.4. Similarly to S4.1, positive examples are pairs of metabolites and disease-related MeSH extracted from the FORUM KG (q-value $\leq 1e-6$ with BH correction and no weakness), while negative examples are created by random combinations. For the purpose of simulating overlooked metabolites' data, the number of co-mentions (number of articles mentioning the both and supporting the relation) were randomly generated from a binomial distribution. We reworked the section S4.4 to clarify these potential ambiguities.

2.11.6 S4.4 Impact of the carbon skeleton graph on the predictions This should also be discussed in the main text.

We reworked the related paragraph in the main text to better highlight these results. Like the analysis on neglected metabolites, we consider this one as secondary and do not wish to detail the results in the main text.

Reviewer #3:
The authors present a tool (FORUM Literature Propagation) which is designed to help users query disease information relevant to a given metabolite by also querying a metabolic neighbors. This is accomplished by using a predefined network of metabolism (Human1) and querying PubChem for compound details and PubMed for articles containing disease and metabolite information. The authors have created a tool that is useful in finding potential associations of disease to metabolite, even when no articles have been published related to the specific metabolite in question This appears to be a useful tool for hypothesis generation, but should be used with caution as the results are inferred associations that may be skewed by regulatory mechanisms, the presence of highly studied metabolites, and highly 'promiscuous' metabolites which interact in a number of different pathways.

This review will focus primarily on the usability of the tool and the communication of that within the text.

Summarily I find this to be a well written manuscript that does a good job of outlining the problem/need and appears to offer a solution. I do have some suggestions for clarifying the manuscript:

3.1 In the first paragraph of Method and Data Description the authors define a 'metabolic neighborhood' as "compound consists of the metabolites that can be reached through a sequence of biochemical reactions." Authors go on to reference the tools used to build and constrain the model. It would be additive to add some brief description to what was done prior, in addition to the more through explanation in supplemental information.

We thank the reviewer for helping us improve the clarity of the manuscript. We decided to add the following sentence in the corresponding section to better describe what is done with the atom-mapping procedure: “This results in a compound graph, built by linking two compounds when they share at least one carbon and have a substrate-product relationship in at least one reaction.”.

3.2 Continuing the above point this manuscript would be aided by a workflow diagram clearly illustrating the order of operations including key elements such as: user input, local database searching (Human1?), and PubMed/PubChem searching, result aggregation.

We thank the reviewer for this idea. Following this suggestion we added such a diagram. However, since the workflow encompasses various components, such as the extraction of FORUM associations and the conversion of the metabolic network into RDF, which are not the primary focus of our article, we have chosen to include the workflow diagram in the supplementary materials.

3.3 Figure 1 aids the reader to visualize FORUMs literature query process. However, it is a very dense figure that is difficult to extrapolate meaning from without carefully reading the Method and Data Description section. Ideally, this figure would be able to be understood by looking at the figure and its caption (current caption only details Blocks A and B). + Having blocks A-F and metabolites named A-F is also confusing, consider changing metabolites to numbers or Greek letters

We are thankful to the reviewer for pointing this out. We changed the figure annotation in order to make it more self-explaining. We decided to keep the capital letters for Figures’ sub captions and renames the metabolites in lowercase. Some other details were also added to the figure according to different reviewers’ comments.

3.4 What database is being used to define the metabolic network (pathways) and what identifiers are used to search those pathways for metabolic neighbors? Is this the pruned Human1 metabolic network and CIDs? More clarity here, would also be addressed by adding the workflow diagram suggested previously.

The metabolic network comes from a conversion of the Human1 SBML into RDF, and its content can be accessed from its own species identifiers or any referenced external identifier (CID, Chebi…) using the closeMatch property in SPARQL query. We added the workflow diagram to make this clearer, and, in addition to the data structure schema in the on-line documentation, we plan on adding pre-built example queries on the endpoint web page in the next release to make the search process more comprehensible.

3.5 Are the total number of metabolites available to use in this tool the 2704 mentioned in the Analysis section? Can this curated library be downloaded?

2704 is the total number of metabolites in the pruned version of the Human 1 v1.7 metabolic network. Among these metabolites, those with less than 100 annotated articles were considered as overlooked (2113 metabolites) and selected for analysis. Recall that this initial selection only serves as a prioritization and the method can be applied on the complete dataset. The library can be downloaded in a tabular file and in RDF format from the FTP server. Its content can be queried using the listed endpoint, from which the list of mentioned compounds can be retrieved in tabular format.

3.6 It appears to be a major limitation of this tool that over half of the 2704 metabolites do not have annotated PubChem CIDs, limiting the effectiveness of the tool in searching disease relevance.

Indeed, despite efficient cross-reference retrieval initiatives such as MetaNetX, many metabolites do not have annotated CID and thus can’t be linked to any scientific literature. It is worth noting that the proposed method purposely alleviates such shortcoming by providing plausible associations for such compounds.
However, while some of those non-referenced compounds might not have any mentioning articles (or too few to confidently derive associations), some could have brought useful information and improved the associations regarding the former.
We could not find any means to know how non-referenced compounds are distributed among those two groups. However, we believe that most compounds without CID would have yielded few or no articles, since we see a correlation between the number of mentioning articles and the prevalence of curated entries of such compounds in many databases, estimated by the number of retrieved cross-reference annotations.

A Boxplot comparing the number of annotated articles among metabolic species in the network with more of fewer annotations than the median can be found in the attached document.

3.7 In the discussion section the authors simply state "many cannot be mapped to their corresponding PubChem identifier." Why? PubChem has over 100 million compounds, surely all the metabolites in the Human1 database have PubChem entries.

Some entries in metabolic models correspond to abstract entities rather than metabolites, such as “biomass” or “lipid pool”, which can explain a lack of PubChem reference. It is possible that other entries corresponding to metabolites could be found in the PubChem database, but the ambiguity and variability of chemical entity naming made such retrieval difficult and their mapping is still on hold, waiting for the ongoing collaborative refinement of Human1 and target databases to fix the issue.

3.8 Figure 2B has a typo in the caption. 16.5% should be 18.5% based on what is shown in the figure.

We thank the reviewers for noticing this typo. It has been corrected in the newer version.

3.9 Did the authors intend to say there are 1336 articles with PubChem identifiers in the figure 2 caption?

We are very grateful to this reviewer for spotting this error in the caption. Indeed, we meant 1336 compounds.

3.10 Figure 3 shows all 3 methods tested produced better AUC than Baseline-Freq showing the utility of metabolic neighborhoods however the graph gives this reader the impression that Baseline-DN and both α methods give very similar results. Perhaps a second panel of figure 3 could more articulately illustrate the difference in the methods as related to the neighbourhood parameter.
Figure 3 provides an overview of the evaluation of the built prior, a crucial component of our proposed approach. We assess the extent to which the literature in the metabolic neighbourhood of a compound contains relevant information about its biomedical context and can effectively guide predictions for rarely mentioned compounds. Baseline-DN serves as a strong baseline that shares similar assumptions with the approach when $\alpha = 0$, but lacks the Bayesian component that incorporates an initial prior assuming independence (see "Mixing neighbouring literature to build a prior").
We established a direct connection between our approach and Baseline-DN by highlighting that when all direct neighbours have similar numbers of annotated articles and are not overlooked (with negligible shrinkage), the method with $\alpha = 0$ behaves similarly to Baseline-DN. Then, close performances are expected. Furthermore, while the constructed prior is the sole source of information for predictions on

metabolites without any annotated articles, predictions for metabolites with at least one annotated article rely on the posterior distribution. We assess this aspect by simulating overlooked metabolites in Supplementary S4.4, demonstrating the advantages of the Bayesian component in handling misleading observations.
We acknowledge that these results were not sufficiently emphasized in the main text, and we have reworked this paragraph to address the suggestions, also considering the comments raised by reviewer 2.

3.11 Figure 4 and 5 it is not clear if there are any differences in the Contributor Odds based on the color scaling almost all sections appear the same shade of red.
Figures 4 and 5 exhibit instances where the neighbourhood strongly suggests a relationship between the targeted metabolite and the disease. In these cases, the individual logOdds values for all contributors are high (>~ 50), resulting in almost the same colour based on the applied scale. The range of possible logOdds values is large ([< -100, > 100]) and we acknowledge that small differences are difficult to appreciate. However, considering the logarithmic scale used for logOdds, the primary purpose of this colour scale is to quickly identify negative, neutral, or positive contributions and the transitions between these states, such as in Supplementary Figures S3, S5, and S13. Additionally, we have provided the complete list of logOdds values in the supplementary tables referenced in the corresponding figures' legend. All other examples of figures can be browsed here: https://forum-static-files.semantic-metabolomics.fr

3.12 How are Specie IDs assigned? It is not an identifier I have used. Can InChiKey, SMILES, CID, HMBD IDs, be readily converted to Specie IDs? If so, how?

Species IDs are manually or automatically assigned during reconstruction, following conventions that vary between models. Nonetheless, each species is manually curated to be annotated with external identifiers such as CID or CHEBI to ensure interoperability. Databases such as MetaNetX gather all cross-references between such external databases and identifier systems such as InChIKey or SMILES, and thus enable conversions. In practice, one can directly access a compound from a metabolic network in FORUM using its ID in query, or alternatively access it by querying a compound with an annotation that match directly or indirectly a given identifier under any system present in the MetaNetX dataset.

3.13 Is there a mechanism in place for limiting the scope of the query? For instance, if I am studying denovo purine synthesis and quantifying metabolites from the pentose phosphate pathway is there a way to exclude contributions of glucose-6-phosphate as it would surely skew my results towards glycolysis intermediates.

We appreciate the reviewer's insightful question. Indeed, it is possible for highly influential compounds to dominate the suggestions and overshadow the contributions of other contributors (as discussed in the Limitation section, using ethanol as an example). In such cases, it may be desirable to adjust the weight vectors to either suppress certain compounds. One can directly modify the weight matrix that contains the $w_{i,k}$ values for all possible pairs of metabolites. Then, the influence neighbourhood of each metabolite is entirely customizable and is not limited by what may be imposed by the structure of the network. These weight matrix matrices are stored in a cache directory created by the method in the working directory, allowing for direct edits of the weight values.

3.14 Can the authors elaborate on why Human1 was chosen over larger metabolic pathway libraries (KEGG, SMPDB, Biocyc, Reactome)?

One aspect of such choice is the licensing of the data, some, despite being freely accessible, forbid bulk download or restrict their usage to a scope that is not compatible with the proposed work under FAIR principles.
Another aspect is related to the curation of links. As this work relies on guilt by association principle, the quality of the relationship used is of utmost importance to avoid propagation of irrelevant information. Human1/HumanGEM is not a data repository but a functional metabolic model. As such, simulations

guarantee agreement between the data and expected behaviors and allows for another degree of refinement, often correcting small inconsistencies that could be found in larger generic databases.
