# Supplementary material for: Suggesting disease associations for overlooked metabolites using literature from metabolic neighbors
Source: Gigascience. 2023 Sep 15;12:giad065. doi: 10.1093/gigascience/giad065 (PMC10502579; doi:10.1093/gigascience/giad065)
Supplement: giad065_Response_to_Reviewer_Comments_Revision_1 [file giad065_response_to_reviewer_comments_revision_1.pdf]

Author's Response To Reviewer Comments

Thank you for considering our manuscript for publication. We are grateful for this opportunity, and we hope the minor revisions meet the journal's requirements.
We thank the reviewers for their valuable feedback, which undoubtedly strengthened this work.
