# Supplementary material for: Suggesting disease associations for overlooked metabolites using literature from metabolic neighbors
Source: Gigascience. 2023 Sep 15;12:giad065. doi: 10.1093/gigascience/giad065 (PMC10502579; doi:10.1093/gigascience/giad065)
Supplement: giad065_Reviewer_1_Report_Original_Submission — Biswapriya Misra -- 2/25/2023 Reviewed [file giad065_reviewer_1_report_original_submission.pdf]

Reviewer Report

Title: Suggesting disease associations for overlooked metabolites using literature from metabolic neighbours

Version: Original Submission **Date:** 2/25/2023

Reviewer name: Biswapriya Misra

Reviewer Comments to Author:

Manuscript Number: GIGA-D-23-00014, entitled, " Suggesting disease associations for overlooked metabolites using literature from metabolic neighbours", and submitted to the journal: GigaScience, applied 'guilt by association' principle to literature information for "understudied metabolites" by using a Bayesian framework. It is an interesting manuscript, an active area of research and would have an interest in the metabolomics research community.
However, this reviewer would like to help improve the manuscript and scope of the work with the following suggestions:
1. A list/ DB of all such "overlooked metabolites" and their chemical class distribution/ ChemRICH sort of enrichment would help the readers capture the correct information.
2. How did/ would the tool perform with "very well known metabolites" for example say, phenylalanine or proline or citric acid ?
3. How does one check for "literature/ reporting biases" for the highly reported vs lowly reported metabolites in the manuscripts?
4. Does this approach distinguish for targeted vs untargeted metabolomics paper based hits ?
5. "Overlooked metabolites" need to be defined well, upfront for clarity.
6. large fraction of metabolites are rarely or never mentioned in the literature: What is a good estimate from the authors? A numerical value would be informative here.
7. Too many terms used does not help: overlooked metabolites vs. understudied metabolites and so on. Please use a singular term for consistency.
8. Method and data description section is too wordy, need to be shortened and need to use mathematical expressions whenever applicable.

Methods

Are the methods appropriate to the aims of the study, are they well described, and are necessary controls included? Choose an item.

Conclusions

Are the conclusions adequately supported by the data shown? Choose an item.

Reporting Standards

Does the manuscript adhere to the journal's guidelines on minimum standards of reporting? Choose an item.

Choose an item.

Statistics

Are you able to assess all statistics in the manuscript, including the appropriateness of statistical tests used? Choose an item.

Quality of Written English

Please indicate the quality of language in the manuscript: Choose an item.

Declaration of Competing Interests

Please complete a declaration of competing interests, considering the following questions:

- Have you in the past five years received reimbursements, fees, funding, or salary from an organisation that may in any way gain or lose financially from the publication of this manuscript, either now or in the future?
- Do you hold any stocks or shares in an organisation that may in any way gain or lose financially from the publication of this manuscript, either now or in the future?
- Do you hold or are you currently applying for any patents relating to the content of the manuscript?
- Have you received reimbursements, fees, funding, or salary from an organization that holds or has applied for patents relating to the content of the manuscript?
- Do you have any other financial competing interests?
- Do you have any non-financial competing interests in relation to this paper?

If you can answer no to all of the above, write 'I declare that I have no competing interests' below. If your reply is yes to any, please give details below.

I declare that I have no competing interests

I agree to the open peer review policy of the journal. I understand that my name will be included on my report to the authors and, if the manuscript is accepted for publication, my named report including any attachments I upload will be posted on the website along with the authors' responses. I agree for my report to be made available under an Open Access Creative Commons CC-BY license (http://creativecommons.org/licenses/by/4.0/). I understand that any comments which I do not wish to be included in my named report can be included as confidential comments to the editors, which will not be published.

Choose an item.

To further support our reviewers, we have joined with Publons, where you can gain additional credit to further highlight your hard work (see: https://publons.com/journal/530/gigascience). On publication of this paper, your review will be automatically added to Publons, you can then choose whether or not to claim your Publons credit. I understand this statement.

Yes Choose an item.
