# Supplementary material for: Suggesting disease associations for overlooked metabolites using literature from metabolic neighbors
Source: Gigascience. 2023 Sep 15;12:giad065. doi: 10.1093/gigascience/giad065 (PMC10502579; doi:10.1093/gigascience/giad065)
Supplement: giad065_Reviewer_2_Report_Original_Submission — Tara Eicher, M.S. -- 3/3/2023 Reviewed [file giad065_reviewer_2_report_original_submission.pdf]

Reviewer Report

Title: Suggesting disease associations for overlooked metabolites using literature from metabolic neighbours

Version: Original Submission **Date:** 3/3/2023

Reviewer name: Tara Eicher, M.S.

Reviewer Comments to Author:

Overall Notes
This work is innovative and will provide an important contribution to the computational metabolomics field. The experiments and methodology are well-designed and executed, and the software is also well-documented.
That being said, the structure and writing of the manuscript needs to be reworked. There are several areas of the text where descriptions are unclear, detailed below. Some of the text is also out of order, e.g. weights are shown in a figure before they are defined, and TPR and FPR are reported without describing the dataset. Finally, there are several Supplementary experiments that are never mentioned in the main text. At least a brief description of these should be given in the main text and then the Supplementary referenced.
Abstract
Some of the language used here is difficult to read or unclear. In particular:
* I believe you mean to say that signatures… "have a strong added value", not "are a strong-added value".
* "we extend the 'guilt by association' principle to literature information by using a Bayesian framework". This is vague. Instead, briefly explain how you use a Bayesian framework to determine guilt by association.
* "1,047 overlooked metabolites". Do you mean metabolites not in the literature?
Your method uses knowledge about metabolic interactions/reactions to generate the graph, but this is not mentioned at all in the abstract. The abstract should explain that this knowledge is being used and describe how it is complementary to the literature.
Background
* It is irrelevant to mention exponential growth. This detracts from the main point, which is the imbalanced knowledge distribution.
* "This topic has received much attention for genes and proteins…" Can you provide some citations?
* "has an impact on the quantity and quality of gene annotations in databases" - in what way? Can you be more specific?
* The first sentence of the second paragraph can be removed.
* You discuss the issue of inaccurate identification as being related to the number of articles mentioning a compound. I feel that these are two separate issues. The first is related to identification, and the second is related to discussion of identified metabolites in the literature. The section regarding identification should be removed.

* "Guilt by association principle", not hypothesis.
* "The method returns several predictors to evaluate whether a significant proportion of the articles mentioning a metabolite would also mention a disease." Do you mean to say that the predictors predict whether or not the metabolite is related to the disease?
* You mention that you used FORUM Knowledge Graph to obtain your metabolite-disease associations. What about the metabolic neighborhoods? You should explain where these were obtained.

Method and Data Description

* How do you define "rarely mentioned"? Is there a cutoff criteria used?
* Does "amount of literature" mean number of articles?
* How is "far distant" defined? It seems that you mean to say that one metabolite's influence on another decreases as the number of reactions separating them increases. Is this correct?
* Show Figure 1 as soon as it is mentioned. This goes for the other figures as well.
* You should explain more about the shrinkage procedure here. It isn't clear what you mean.
* In Figure 1C, there appears to be a stack of papers in a pink box in both the numerator and the denominator. What does this mean?
* "Then, we build the prior distribution for A, by mixing the probability distributions of each contributor (see Figure 1.E) according to their weights estimated in the previous step (Figure 1.C)". This is the first time you mention weights. You need to describe what the weights correspond to and how they are calculated first.
* "Then, we build the prior distribution for A, by mixing the probability distributions of each contributor (see Figure 1.E) according to their weights estimated in the previous step (Figure 1.C)". This sentence is unclear. Please revise.
* "Finally, several diagnostic values such as Entropy allow to assess the composition of the built prior (See Supplementary S1.3)". Either name all of the diagnostic values here or move Entropy to the supplementary and out of the main text.
* "Entropy evaluates the good balance of contributions in the prior. The more metabolites contribute to the mixture and the more their weights are uniformly distributed, the higher the entropy." This is not a clear explanation. Please explain mathematically what entropy is and what it represents here.

Analyses

Unbalanced distribution of the literature related to chemical compounds

* At the end of the first paragraph, it should be "is cumulatively less than the literature associated with glucose…"
* Can metabolites without a CID be found in the metabolic network? If not, then you should not discuss those metabolites with an unannotated CID.

Evaluation of the prior computation

* "and is set to Î± = 0 for the direct neighbourhood and Î± = 0.4 for a larger one". Are these the only two values you consider? If so, why? How large is "larger"? This should also go in the methods section.
* "All tested approaches outperform Baseline-Freq, showing the benefit of examining the neighbouring literature." This experiment needs to be explained in the main text. How did you define TPR and FPR?

Suggesting relations with diseases for overlooked metabolites

* "However, by re-evaluating these predictions using a right-tailed Fisher exact Test (BH correction and selecting those with q.value <= 0.05), we found that â‰ˆ 50% of them (925) would not have been found

significant". Can you please explain this experiment further?
* "These relations are still weakly supported, nevertheless, our method showed that they are consistent with the neighbourhood." What does this mean?
* Why do you want high entropy in Table 1? This isn't clear if the reader doesn't know what entropy refers to here.

Limitations

"Although we kept it in our analysis for sake of exhaustively…" It is not clear what this means.

Methods

Your equations are not numbered correctly. Your equation (1) should be equation (2). It looks like you have 14 equations total, but only one is numbered.

Settings

* How did you choose the cutoff in equation (1)?
* "These aspects are illustrated in Figure 1.B: B…" This entire paragraph should be moved up to the Method and Data Description section.

Mixing neighbouring literature to build a prior

Explain what the Beta distribution parameters mean in the context of this study in detail and why you chose a Beta distribution.

Updating prior and selecting novel associations

"In turn, Log2FC is much more sensitive to outlier contributors than LogOdds". Please provide a citation for this.

References

* Check the formatting for #27. It is overlapping the text in the right-hand column.
* If you're going to discuss the Pareto Principle (28), try to find a review article that describes this principle rather than referencing a textbook.

Supplementary Material

S1.3 Diagnostic Values

Here, it is clear what you mean by Entropy and why you want the value to be high rather than low. This should go in the main text. However, it is concerning that the meaning of the entropy cutoff changes with respect to the number of contributors. Consider using a weighted metric that has the same meaning regardless of the number of contributors.

S2. Supplementary Tables

Why are your LogOdds values infinite? If this is an issue with taking the log of 0, then you should set a cutoff such that the values do not go to infinity.

S3. Supplementary Figures

* Figure S3 is low-resolution and difficult to read. Please include a higher-resolution version of this figure.
* The figures don't seem to match up with their references in the main text.
* All supplementary figures should be here (including S1 and all figures after S3).
* It is not clear what Figure S4C refers to in the main text.

S4. Supplementary Materials

S4.1 Damping factor Î± and theoretical sample size Î½: benchmark

The validation dataset needs to be described before the results are presented. At this point, the reader

has no idea what the validation set is.
S4.3 Evaluation using simulated overlooked metabolites
* These results should be highlighted in the main text.
* "Focusing on overlooked metabolites, the most challenging scenarios are those where positive examples apparently show no co-mentions, and conversely, when co-mentions (e.g. anecdotal) wrongly support negative examples." Please highlight how you determined positive examples, negative examples, and co-mentions here.
S4.4 Impact of the carbon skeleton graph on the predictions
This should also be discussed in the main text.

Methods

Are the methods appropriate to the aims of the study, are they well described, and are necessary controls included? Choose an item.

Conclusions

Are the conclusions adequately supported by the data shown? Choose an item.

Reporting Standards

Does the manuscript adhere to the journal's guidelines on [minimum standards of reporting?]() Choose an item.

Choose an item.

Statistics

Are you able to assess all statistics in the manuscript, including the appropriateness of statistical tests used? Choose an item.

Quality of Written English

Please indicate the quality of language in the manuscript: Choose an item.

Declaration of Competing Interests

Please complete a declaration of competing interests, considering the following questions:

- Have you in the past five years received reimbursements, fees, funding, or salary from an organisation that may in any way gain or lose financially from the publication of this manuscript, either now or in the future?
- Do you hold any stocks or shares in an organisation that may in any way gain or lose financially from the publication of this manuscript, either now or in the future?

- Do you hold or are you currently applying for any patents relating to the content of the manuscript?
- Have you received reimbursements, fees, funding, or salary from an organization that holds or has applied for patents relating to the content of the manuscript?
- Do you have any other financial competing interests?
- Do you have any non-financial competing interests in relation to this paper?

If you can answer no to all of the above, write 'I declare that I have no competing interests' below. If your reply is yes to any, please give details below.

I declare that I have no competing interests

I agree to the open peer review policy of the journal. I understand that my name will be included on my report to the authors and, if the manuscript is accepted for publication, my named report including any attachments I upload will be posted on the website along with the authors' responses. I agree for my report to be made available under an Open Access Creative Commons CC-BY license (http://creativecommons.org/licenses/by/4.0/). I understand that any comments which I do not wish to be included in my named report can be included as confidential comments to the editors, which will not be published.

Choose an item.

To further support our reviewers, we have joined with Publons, where you can gain additional credit to further highlight your hard work (see: https://publons.com/journal/530/gigascience). On publication of this paper, your review will be automatically added to Publons, you can then choose whether or not to claim your Publons credit. I understand this statement.

Yes Choose an item.
