# Supplementary material for: Suggesting disease associations for overlooked metabolites using literature from metabolic neighbors
Source: Gigascience. 2023 Sep 15;12:giad065. doi: 10.1093/gigascience/giad065 (PMC10502579; doi:10.1093/gigascience/giad065)
Supplement: giad065_Reviewer_3_Report_Original_Submission — Brian DeFelice -- 3/15/2023 Reviewed [file giad065_reviewer_3_report_original_submission.pdf]

Reviewer Report

Title: Suggesting disease associations for overlooked metabolites using literature from metabolic neighbours

Version: Original Submission **Date:** 3/15/2023

Reviewer name: Brian DeFelice

Reviewer Comments to Author:

The authors present a tool (FORUM Literature Propagation) which is designed to help users query disease information relevant to a given metabolite by also querying a metabolic neighbors. This is accomplished by using a predefined network of metabolism (Human1) and querying PubChem for compound details and PubMed for articles containing disease and metabolite information. The authors have created a tool that is useful in finding potential associations of disease to metabolite, even when no articles have been published related to the specific metabolite in question This appears to be a useful tool for hypothesis generation, but should be used with caution as the results are inferred associations that may be skewed by regulatory mechanisms, the presence of highly studied metabolites, and highly 'promiscuous' metabolites which interact in a number of different pathways.
This review will focus primarily on the usability of the tool and the communication of that within the text. Summarily I find this to be a well written manuscript that does a good job of outlining the problem/need and appears to offer a solution. I do have some suggestions for clarifying the manuscript:
* In the first paragraph of Method and Data Description the authors define a 'metabolic neighborhood' as "compound consists of the metabolites that can be reached through a sequence of biochemical reactions." Authors go on to reference the tools used to build and constrain the model. It would be additive to add some brief description to what was done prior in addition to the more through explanation in supplemental information.
* Continuing the above point this manuscript would be aided by a workflow diagram clearly illustrating the order of operations including key elements such as: user input, local database searching (Human1?), and PubMed/PubChem searching, result aggregation.
* Figure 1 aids the reader to visualize FORUMs literature query process. However, it is a very dense figure that is difficult to extrapolate meaning from without carefully reading the Method and Data Description section. Ideally, this figure would be able to be understood by looking at the figure and its caption (current caption only details Blocks A and B).
o Having blocks A-F and metabolites named A-F is also confusing, consider changing metabolites to numbers or Greek letters
* What database is being used to define the metabolic network (pathways) and what identifiers are used to search those pathways for metabolic neighbors? Is this the pruned Human1 metabolic network and CIDs? More clarity here, would also be addressed by adding the workflow diagram suggested previously.
* Are the total number of metabolites available to use in this tool the 2704 mentioned in the Analysis section? Can this curated library be downloaded?

* It appears to be a major limitation of this tool that over half of the 2704 metabolites do not have annotated PubChem CIDs, limiting the effectiveness of the tool in searching disease relevance.
o In the discussion section the authors simply state "many cannot be mapped to their corresponding PubChem identifier." Why? PubChem has over 100 million compounds, surely all the metabolites in the Human1 database have PubChem entries.
* Figure 2B has a typo in the caption. 16.5% should be 18.5% based on what is shown in the figure.
o Did the authors intend to say there are 1336 articles with PubChem identifiers in the figure 2 caption?
* Figure 3 shows all 3 methods tested produced better AUC than Baseline-Freq showing the utility of metabolic neighborhoods however the graph gives this reader the impression that Baseline-DN and both Î± methods give very similar results. Perhaps a second panel of figure 3 could more articulately illustrate the difference in the methods as related to the neighborhood parameter.
* Figure 4 and 5 it is not clear if there are any differences in the Contributor Odds based on the color scaling almost all sections appear the same shade of red.
* How are Specie IDs assigned? It is not an identifier I have used. Can InChiKey, SMILES, CID, HMBD IDs, be readily converted to Specie IDs? If so, how?
* Is there a mechanism in place for limiting the scope of the query? For instance, if I am studying denovo purine synthesis and quantifying metabolites from the pentose phosphate pathway is there a way to exclude contributions of glucose-6-phosphate as it would surely skew my results towards glycolysis intermediates.
* Can the authors elaborate on why Human1 was chosen over larger metabolic pathway libraries (KEGG, SMPDB, Biocyc, Reactome)?

Methods

Are the methods appropriate to the aims of the study, are they well described, and are necessary controls included? Choose an item.

Conclusions

Are the conclusions adequately supported by the data shown? Choose an item.

Reporting Standards

Does the manuscript adhere to the journal's guidelines on [minimum standards of reporting?]() Choose an item.

Choose an item.

Statistics

Are you able to assess all statistics in the manuscript, including the appropriateness of statistical tests used? Choose an item.

Quality of Written English

Please indicate the quality of language in the manuscript: Choose an item.

Declaration of Competing Interests

Please complete a declaration of competing interests, considering the following questions:

- Have you in the past five years received reimbursements, fees, funding, or salary from an organisation that may in any way gain or lose financially from the publication of this manuscript, either now or in the future?
- Do you hold any stocks or shares in an organisation that may in any way gain or lose financially from the publication of this manuscript, either now or in the future?
- Do you hold or are you currently applying for any patents relating to the content of the manuscript?
- Have you received reimbursements, fees, funding, or salary from an organization that holds or has applied for patents relating to the content of the manuscript?
- Do you have any other financial competing interests?
- Do you have any non-financial competing interests in relation to this paper?

If you can answer no to all of the above, write 'I declare that I have no competing interests' below. If your reply is yes to any, please give details below.

I declare that I have no competing interests

I agree to the open peer review policy of the journal. I understand that my name will be included on my report to the authors and, if the manuscript is accepted for publication, my named report including any attachments I upload will be posted on the website along with the authors' responses. I agree for my report to be made available under an Open Access Creative Commons CC-BY license (http://creativecommons.org/licenses/by/4.0/). I understand that any comments which I do not wish to be included in my named report can be included as confidential comments to the editors, which will not be published.

Choose an item.

To further support our reviewers, we have joined with Publons, where you can gain additional credit to further highlight your hard work (see: https://publons.com/journal/530/gigascience). On publication of this paper, your review will be automatically added to Publons, you can then choose whether or not to claim your Publons credit. I understand this statement.

Yes Choose an item.
